# Single-dose azithromycin for child growth in Burkina Faso: a randomized controlled trial

**DOI:** 10.1186/s12887-021-02601-7

**Published:** 2021-03-17

**Authors:** Ali Sié, Boubacar Coulibaly, Clarisse Dah, Mamadou Bountogo, Mamadou Ouattara, Guillaume Compaoré, Jessica M. Brogdon, William W. Godwin, Elodie Lebas, Thuy Doan, Benjamin F. Arnold, Travis C. Porco, Thomas M. Lietman, Catherine E. Oldenburg

**Affiliations:** 1 Centre de Recherche en Santé de Nouna, Nouna, Burkina Faso; 2 Francis I Proctor Foundation, University of California, 513 Parnassus Ave, Box 0412, San Francisco, CA 94143 USA; 3 Department of Ophthalmology, University of California, San Francisco, USA; 4 Department of Epidemiology & Biostatistics, University of California, San Francisco, USA

**Keywords:** Azithromycin, Undernutrition, Child growth, Randomized controlled trial

## Abstract

**Background:**

In lower resource settings, previous randomized controlled trials have demonstrated evidence of increased weight gain following antibiotic administration in children with acute illness. We conducted an individually randomized trial to assess whether single dose azithromycin treatment causes weight gain in a general population sample of children in Burkina Faso.

**Methods:**

Children aged 8 days to 59 months were enrolled in November 2019 and followed through June 2020 in Nouna Town, Burkina Faso. Participants were randomly assigned to a single oral dose of azithromycin (20 mg/kg) or matching placebo. Anthropometric measurements were collected at baseline and 14 days and 6 months after enrollment. The primary anthropometric outcome was weight gain velocity in g/kg/day from baseline to 14 days and 6 months in separate linear regression models.

**Results:**

Of 450 enrolled children, 230 were randomly assigned to azithromycin and 220 to placebo. Median age was 26 months (IQR 16 to 38 months) and 51% were female. At 14 days, children in the azithromycin arm gained a mean difference of 0.9 g/kg/day (95% CI 0.2 to 1.6 g/kg/day, *P* = 0.01) more than children in the placebo arm. There was no difference in weight gain velocity in children receiving azithromycin compared to placebo at 6 months (mean difference 0.04 g/kg/day, 95% CI − 0.05 to 0.13 g/kg/day, *P* = 0.46). There were no significant differences in other anthropometric outcomes.

**Conclusions:**

Transient increases in weight gain were observed after oral azithromycin treatment, which may provide short-term benefits.

**Clinical trials registration:**

ClinicalTrials.gov NCT03676751. Registered 19/09/2018.

**Supplementary Information:**

The online version contains supplementary material available at 10.1186/s12887-021-02601-7.

## Background

Biannual oral mass azithromycin distribution to children aged 1–59 months has been shown to reduce all-cause child mortality in some settings in sub-Saharan Africa [1–4]. The primary mechanism is likely via reduction in infectious burden [5]. Undernutrition is an underlying cause in as many as 50% of child deaths [6]. Undernutrition may predispose children to infection, and infection may prevent weight gain during the period of recovery [7]. Children experiencing undernutrition have elevated risk of mortality from infection compared to those with infection who are not undernourished [8]. Antibiotics have growth-promoting effects in children with recognized clinical illness such as HIV, and have been shown to reduce mortality in some settings in children with severe acute malnutrition [9, 10]. Antibiotics may lead to increased weight gain via reduction in clinical and sub-clinical infections, through reducing inflammation, or through alteration of gut microbiome [7, 11]. Azithromycin may affect growth and nutrition outcomes via such pathways, contributing to overall observed reductions in mortality.

Studies of mass distribution of azithromycin for trachoma control have evaluated the effect of azithromycin distributed to entire communities on anthropometric measurements in children [12–15]. These studies have not shown evidence that mass azithromycin distribution or increased frequency of mass azithromycin distribution significantly affects indicators of nutritional status including stunting, underweight, and wasting. These studies randomized entire communities to different azithromycin distribution strategies or to no azithromycin distribution. They were therefore limited by the number of clusters randomized and by lack of longitudinal measurements.

We recently conducted an individually randomized trial designed to assess potential mechanisms for any effect of azithromycin on childhood mortality, including alterations in the pediatric microbiome and infections. Here, we report linear and ponderal growth in children receiving a single oral dose of azithromycin compared to placebo with longitudinal measurements. We hypothesized that children receiving azithromycin would have improved growth compared to those receiving placebo.

## Methods

### Study design

This study was a 1:1 individually randomized placebo-controlled trial comparing single dose azithromycin (20 mg/kg) to matching placebo. This study was registered at clinicaltrials.gov before recruitment of the first participant (ClinicalTrials.gov NCT03676751, registered 19/09/2018).

### Study setting

This study was conducted in the town of Nouna, Burkina Faso in northwestern Burkina Faso. Nouna is the capital of the province of Kossi and has approximately 20,000 residents. Children were enrolled in November 2019 during the annual harvest season and the primary endpoint occurred in June 2020, at the beginning of the rainy season. All primary endpoint visits occurred during the pre-specified visit window, which allowed visits to occur up to 6 weeks after the scheduled time point (6 months after enrollment).

### Ethics

This study was reviewed and approved by the Institutional Review Board at the University of California, San Francisco and the Comité National d’Ethique pour la Recherche (National Ethics Committee of Burkina Faso) in Ouagadougou, Burkina Faso. Written informed consent was obtained from the guardian of each child prior to participation.

### Eligibility and recruitment

Study staff visited households with children under 5 years of age known to be resident and informed caregivers about the study. Interested caregivers were asked to bring their children to the Nouna District Hospital for eligibility assessment and enrollment. Eligibility criteria included: 1) between 8 days and 59 months of age; 2) living in the town of Nouna and not planning to move during the 6-month study period; 3) ability to feed orally (and thus receive the oral study medication); and 4) no known allergies to macrolides. Caregivers were given a small reimbursement for transportation costs for each study visit (approximately USD$1.85 per visit).

### Study procedures

All study procedures occurred at the Nouna District Hospital by trained study staff and healthcare workers. Participants were evaluated at baseline, 14 days after enrollment, and 6 months after enrollment. At baseline, caregivers completed a short baseline questionnaire that included mother’s age and educational status and the child’s breastfeeding status (Supplemental Material).

### Randomization and masking

Children were randomized in a 1:1 fashion to azithromycin or matching placebo. The allocation sequence was generated through unrestricted randomization by the study biostatisticians with no blocking or stratification. The randomization sequence was implemented by assigning a letter to azithromycin and placebo and assigning each child a study identification number that was linked to the treatment letter in the study’s mobile application. Allocation concealment was achieved as study staff did not have access to the randomization list and were not aware of which treatment letter was associated with a study identification number until the child had been enrolled in the study and completed their baseline assessments. The placebo preparation was identical to azithromycin in composition for all ingredients except the active ingredient, and they were identical in taste, smell, and appearance. Study staff and investigators were not aware of which treatment letter was associated with azithromycin or placebo.

### Interventions

Azithromycin and matching placebo were prepared as pediatric oral suspension. A single oral dose of 20 mg/kg azithromycin or equivalent volume of placebo was offered to each enrolled child. Weight measurements were taking to facilitate dosing in children under 12 months of age, and a height stick approximation was used for those 12 months of age and over, as is done in trachoma control programs [16, 17]. All study treatments were directly observed and study staff indicated in the mobile application if the child did not receive their randomized treatment assignment.

### Anthropometric measurements

Anthropometric measurements were collected at baseline, 14 days after enrollment, and 6 months after enrollment. Standing height was measured for children who could stand and recumbent length for those who could not (Shorrboard, Weight and Measure LLC, Maryland, USA). Height measurements were recorded in triplicate and the median measurement was used for analysis. Children were weighed standing if able or in the arms of a caregiver after removing heavy garments and jewelry (Seca 874 flat floor scale, Seca GMBH & Co, Hamburg, Germany). MUAC measurements were taken at the midpoint between the tip of the elbow and tip of the shoulder of the left arm using a standard MUAC tape. Weight gain velocity was calculated in grams per kilogram per day (g/kg/day) from the baseline weight measurement. Height gain was calculated in mm/day since baseline. Weight-for-height (WHZ), weight-for-age (WAZ), and height-for-age (HAZ) Z-scores were calculated based on 2006 WHO standards [18].

### Sample size considerations

The sample size for the trial was based on the primary outcome, which was Shannon’s diversity index of the gut microbiome. We estimated that a sample size of 225 children per arm (450 total) would provide at least 80% power for a detectable effect size of 1.02 standard deviations of Shannon’s diversity with no loss to follow-up. We estimated the detectable difference for anthropometric outcomes given this fixed design. Assuming a 2-sided alpha of 5%, and assuming the empirical standard deviations observed in the trial for anthropometry outcomes, we estimated that 225 children per arm would have at 80% power to detect a difference of 0.8 g/kg/d in weight velocity at 14 days (SD = 3.1) and a difference of 0.1 g/kg/d at 6 months (SD = 0.5). For comparisons of weight and height, we adjusted observed standard deviations to account for correlation with baseline (*r =* 0.97) [19]. We estimated the trial had 80% power to detect differences in weight of 0.19 kg (adjusted SD = 0.72) and differences in height of 0.74 cm (adjusted SD = 2.8).

### Statistical methods

The pre-specified primary analysis for anthropometric outcomes was weight gain velocity expressed in g/kg/day analyzed at the 14-day and 6-month time point separately (Supplementary Material). Weight gain velocity was analyzed using a linear regression with a term for the randomized treatment assignment as the sole predictor, without adjustment for baseline weight as baseline weight is included in the outcome calculation. Secondary outcomes included HAZ, WHZ, WAZ, MUAC, and height gain (expressed in mm/day) at the 14-day and 6-month time points separately. Each outcome was analyzed using a linear regression model with terms for the randomized treatment assignment and the baseline value of the outcome. We also analyzed stunting, wasting, and underweight using a cutoff of − 2 SD for HAZ, WHZ, and WAZ respectively using a logistic regression model with a term for randomized treatment assignment. As all outcomes were pre-specified, no adjustment for multiple comparisons was made. No subgroup analyses were conducted due to the relatively small sample size. All analyses were conducted in R (The R Foundation for Statistical Computing, Vienna, Austria).

## Results

**Fig. 1:**
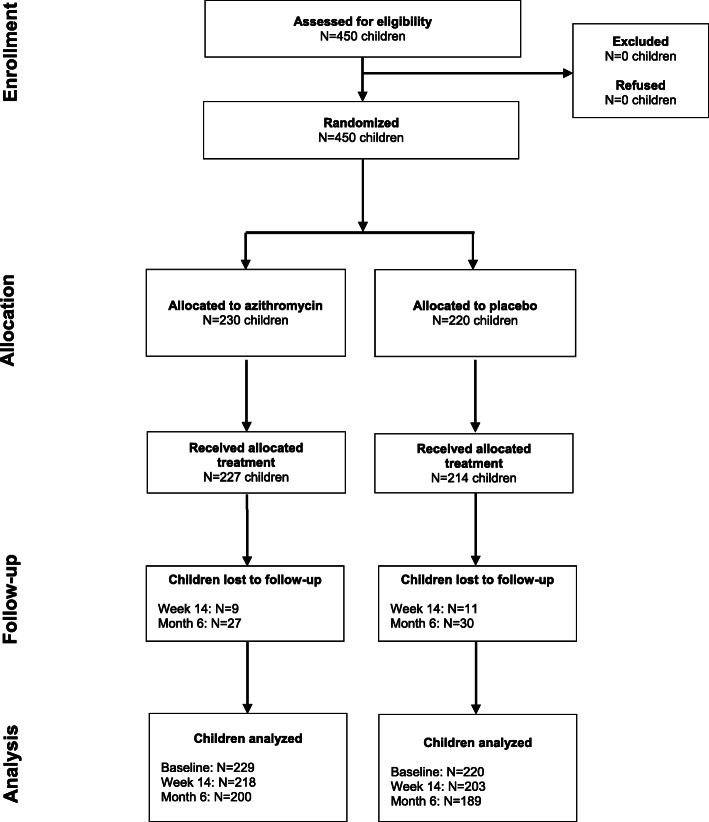
CONSORT flow diagram of study participants

Of 450 children who were eligible and enrolled in the study, 230 were randomized to azithromycin and 220 to placebo (Fig. 1). At baseline, one child had invalid anthropometric measurements and was excluded from analyses. Participants were a median of 26 months of age (interquartile range, IQR, 16 to 38), 51% were female, and 38% were breastfeeding (Table 1). Among participants randomized to azithromycin or placebo, 227 (98.7%) and 214 (97.3%) of participants took the study medication, respectively. At baseline, 18 and 9% of children randomized to azithromycin and placebo, respectively, were classified as underweight based on WAZ, 17 and 13% were stunted based on HAZ, 11 and 6% were wasted based on WHZ, and 5 and 3% were wasted based on MUAC (Table 1). Approximately 6 and 13% of children were lost to follow-up at 14 days and 6 months, respectively. Demographics and anthropometric indices were similar among children who were and were not lost to follow-up (Supplemental Table 1).

**Table 1:** Baseline characteristics by study arm

Characteristic	Azithromycin (***N*** = 230)	Placebo (***N*** = 220)
Age, months (median, IQR)	26 (15 to 39)	26 (17 to 38)
Female sex	53.1%	48.4%
Child is breastfeeding	40.4%	35.6%
Mother’s age (median, IQR)	26.5 (23 to 32)	27 (23 to 33)
Mother’s education		
None	52.2%	55.3%
Primary	26.5%	25.6%
Secondary or higher	21.3%	19.2%
Mid-upper arm circumference, cm (median, IQR)	14 (13 to 15)	14.3 (13.5 to 15)
Weight, kg (median, IQR)	10.9 (8.8 to 13.6)	11.4 (9.4 to 13.4)
Height, cm (median, IQR)	84.7 (74.8 to 95.0)	85.8 (77.3 to 93.5)
Weight-for-height Z-score (mean, SD)	−0.6 (1.2)	−0.4 (1.1)
Height-for-age Z-score (mean, SD)	−1.1 (1.2)	−0.9 (1.3)
Weight-for-age Z-score (mean, SD)	−1.0 (1.1)	−0.7 (1.1)
Wasted (WHZ < -2), N (%)	10.5%	5.9%
Stunted (HAZ < -2), N (%)	16.7%	13.2%
Underweight (WAZ < -2), N (%)	18.0%	9.1%
Mid-upper arm circumference < 12.5 cm	4.8%	3.2%
Malaria RDT Positive^a^	6.1%	3.6%

^a^Rapid diagnostic test (RDT) for malaria was performed in children with tympanic temperature ≥ 37.5 °C

Adverse events at 14 days have been previously reported and were most often gastrointestinal in nature [20]. Caregivers of children randomized to receive azithromycin more often reported vomiting than those receiving placebo (7.2% compared to 1.9%, *P* = 0.01), but there was no significant evidence of a difference in other adverse events including diarrhea (8.1% azithromycin versus 6.2% placebo, *P* = 0.44) and fever (16.7% azithromycin versus 16.7% placebo, *P* > 0.99).

At 14 days, children randomized to azithromycin gained weight significantly faster than those randomized to placebo (mean difference 0.9 g/kg/day, 95% CI 0.2 to 1.6 g/kg/day, *P* = 0.01). In a linear regression model adjusting for baseline weight, at 14 days children in the azithromycin arm were on average 122 g heavier than those in the placebo arm (95% CI 9 to 236 g, *P* = 0.035). There was no significant difference in height gain in children receiving azithromycin compared to placebo at Day 14 (mean difference 0.3 mm/day, 95% CI − 0.07 to 0.7 mm/day, *P* = 0.11). There was no difference in WAZ, WHZ, HAZ, or MUAC between children randomized to azithromycin or placebo at day 14 (Tables 2 and 3). Similarly, there were no significant differences in the odds of underweight, wasting (defined by WHZ or MUAC), or stunting at Day 14 (Tables 2 and 3).

**Table 2:** Weight outcomes at 14 days and 6 months

	*14 Days*				*6 Months*			
	Azithromycin Mean (SD)	Placebo Mean (SD)	Mean Difference or Odds Ratio^a^ (95% CI)	*P*-value	Azithromycin Mean (SD)	Placebo Mean (SD)	Mean Difference or Odds Ratio^a^ (95% CI)	*P*-value
Growth velocity, g/kg/day	2.1 (3.2)	1.2 (4.0)	0.9 (0.3 to 1.5)	0.01	0.6 (0.4)	0.5 (0.6)	0.04 (−0.05 to 0.1)	0.46
Weight, kg	11.5 (3.0)	11.8 (2.9)	0.12 (0.01 to 0.24)	0.035	12.5 (3.0)	12.6 (2.8)	0.07 (− 0.07 to 0.22)	0.32
Mid-upper arm circumference, cm	14.3 (1.2)	14.5 (1.1)	−0.008 (− 0.1 to 0.1)	0.91	14.3 (1.2)	14.5 (1.0)	−0.1 (− 0.25 to 0.03)	0.12
Weight-for-height Z-score	−0.5 (1.0)	− 0.30 (1.1)	− 0.001 (− 0.1 to 0.1)	0.98	−0.8 (1.1)	− 0.6 (1.0)	−0.04 (− 0.2 to 0.11)	0.63
Weight-for-age Z-score	−0.8 (1.0)	− 0.7 (1.1)	0.08 (− 0.01 to 0.02)	0.07	−0.9 (1.0)	− 0.8 (1.0)	0.04 (− 0.07 to 0.14)	0.51
MUAC< 12.5 cm	7.8%	3.4%	1.9 (0.5 to 6.7)	0.32	3.0%	0.5%	4.9 (0.5 to 44.2)	0.16
Wasted (WHZ < -2)	6.0%	5.4%	0.7 (0.3 to 1.9)	0.49	13.4%	11.1%	0.5 (0.2 to 1.3)	0.16
Underweight (WAZ < -2)	13.8%	10.3%	0.4 (0.3 to 1.9)	0.08	9.5%	8.4%	0.9 (0.4 to 2.0)	0.78

^a^All models included a term for the randomized treatment assignment, and all models except for the g/kg/day outcome were adjusted for the baseline measurement of the outcome

**Table 3:** Height outcomes at 14 days and 6 months

	*14 Days*				*6 Months*			
	Azithromycin Mean (SD)	Placebo Mean (SD)	Mean Difference or Odds Ratio^a^ (95% CI)	*P*-value	Azithromycin Mean (SD)	Placebo Mean (SD)	Mean Difference or Odds Ratio^a^ (95% CI)	*P*-value
Height change, mm/day	0.8 (1.7)	0.5 (2.3)	0.3 (−0.07 to 0.7)	0.11	0.4 (0.2)	0.3 (0.2)	0.02 (−0.02 to 0.06)	0.57
Height, cm	86.0 (12.0)	86.6 (11.5)	0.3 (−0.1 to 0.7)	0.15	91.6 (11.5)	91.3 (10.6)	0.3 (−0.3 to 0.8)	0.32
Height-for-age Z-score	−0.9 (1.1)	−0.8 (1.2)	0.08 (− 0.04 to 0.2)	0.21	− 0.8 (1.1)	−0.7 (1.1)	0.05 (− 0.1 to 0.2)	0.55
Stunted (HAZ < -2)	15.1%	13.2%	0.7 (0.4 to 1.6)	0.46	11.9%	10.0%	1.1 (0.5 to 2.5)	0.76

^a^All models included a term for the randomized treatment assignment, and all models except for the g/kg/day outcome were adjusted for the baseline measurement of the outcome

At 6 months, there was no difference in weight gain velocity in children randomized to azithromycin versus placebo (mean difference 0.04 g/kg/day, 95% CI − 0.05 to 0.13 g/kg/day, *P* = 0.46) or absolute difference in weight (mean difference 7 g, 95% CI − 7 to 22 g, *P* = 0.32). Rate of change in height was also similar between the two groups at 6 months (mean difference 0.02 mm/day, 95% CI − 0.02 to 0.06 mm/day, *P* = 0.57). There were no differences in WAZ, WHZ, HAZ, or MUAC or in underweight, wasting, or stunting at 6 months (Tables 2 and 3).

## Discussion

We found significant evidence of a short-term increase in weight gain velocity in children who received a single oral dose of azithromycin compared to placebo, but no difference in longer-term weight gain. Possible mechanisms for short-term increases in weight gain following azithromycin have been hypothesized to include reducing clinical or subclinical infection or via alteration of the microbiome [10]. Mass azithromycin distribution has been shown to reduce mortality primarily in a 3-month period following distribution, but the precise mechanism is unknown [21]. Short-term increases in weight gain may confer some benefit against infection in children that prevents mortality, or a reduction in infection may both increase weight gain and reduce mortality, or the two may be independent. We were unable to assess specific mechanisms for the effect of azithromycin on weight gain in the present analysis. Future studies collecting additional information on infection following azithromycin distribution may allow for further elucidation of the mechanism.

Observational studies in high resource settings have shown an association between antibiotic consumption and increased child growth, leading to concerns about antibiotic consumption and childhood obesity [22, 23]. Potential biases including unmeasured confounding limit the ability to draw causal conclusions from these studies [24]. Randomized controlled trial evidence has generally shown increased growth among children with known acute or chronic illness following antibiotic administration in lower resource settings [10]. Given complex relationships between infection and malnutrition, children with concurrent infection that is treated by antibiotic administration may experience increased growth catch-up as they recover from their illness. A study of amoxicillin compared to placebo for nutritional recovery among children with severe acute malnutrition in Niger found short-term effects of amoxicillin on weight gain velocity but no longer-term benefit in growth outcomes in amoxicillin-treated compared to placebo-treated children [25]. Results from the present study suggest that a similar relationship between antibiotic use and child growth may exist for children without known clinical illness, and the magnitude of the increase in weight gain velocity at 14 days was similar to that in children with severe acute malnutrition receiving amoxicillin compared to placebo at 14 days (mean difference amoxicillin versus placebo 1.2 g/kg/day at 2 weeks) [26]. Antibiotics may be growth-promoting in children for short periods of time after administration.

We found no evidence of an effect of a single dose of azithromycin on height outcomes at either time point. HAZ is often considered an indicator of longer-term nutritional status, which may be influenced by repeated bouts of infection with enteric or other pathogens [27, 28]. Interrupting the undernutrition-infection cycle via antibiotic administration may theoretically improve height-based anthropometric outcomes via treatment of infection. In the present study, a single dose of azithromycin was insufficient to affect longer-term nutritional outcomes. Given that children in this study setting experience a high burden of infection, it is possible that infection re-establishes itself too quickly for a single dose of azithromycin to have any effect on a child’s height. Stunting may also be influenced by perinatal or in utero exposures that can cause children to be born small for gestational age and affect postnatal growth patterns. Antibiotic administration during childhood would not be expected to affect stunting through these pathways, which may in part explain the null effect for height outcomes.

These results must be considered in the context of several limitations. We enrolled a wide age range in the present study (1 to 59 months). Children gain weight at markedly different rates during early childhood [29]. Including a wide age range should not bias estimates due to the randomized and masked nature of the trial, but it likely introduces heterogeneity in outcomes. Given the limited sample size of the trial, we did not have statistical power to conduct age-based subgroup analyses. Such analyses may be useful for better understanding heterogeneity in the effect of azithromycin on growth during different periods of early childhood. Children in the azithromycin arm had a higher prevalence of underweight at baseline compared to those in the placebo arm, indicated some moderate chance imbalances in weight at baseline. All models except for weight gain velocity included the baseline measurement as a covariate, and weight gain velocity is a difference in difference measure, thus taking into account baseline. Small sample sizes precluded subgroup analyses by baseline nutritional status, but it is possible that children with lower nutritional status at baseline are more likely to benefit from azithromycin compared to well-nourished children [30]. Future studies with larger sample sizes could evaluate any benefit of azithromycin in this subgroup. We did not collect information on children who did not participate in the study, and thus are unable to comment on how representative this sample is of children in the recruitment area. Loss to follow-up was less than 15% at each time point, but differences in children who were and were not lost to follow-up could attenuate effect sizes between arms. The 29 children lost by day 14 had similar anthropometry to those who remained, though children who were lost to follow-up by 6 months were slightly more stunted, underweight, and wasted at enrollment than those who were not lost to follow-up. If underweight and stunted children benefitted more from azithromycin treatment, this could attenuate the 6-month effect estimates towards the null. Antibiotic consumption is fairly common in the study population, with children under 5 years of age receiving approximately 2 antibiotic courses per child-year [31]. Over time, the two groups may appear more similar to one another if they are receiving similar amounts of antibiotics between the two groups, which may mask any longer-term differences in weight gain. Finally, this study was conducted in a peri-urban Burkina Faso where childhood undernutrition and infection are common. Burkina Faso experiences strong seasonal patterns in food supplies and malaria incidence. Recruitment and follow-up occurred primarily during the dry season, corresponding to the low malaria transmission season and with less food insecurity. Results may differ in the high food insecurity period if baseline nutritional status is worse and undernourished children have greater improvements in growth with azithromycin compared to well-nourished children.

## Conclusion

Short-term increases in weight as documented in this study may be protective against adverse health outcomes and partially explain any benefit of mass azithromycin distribution for childhood survival. The lack of longer-term difference in weight outcomes suggests that recent concern that antibiotic use contributes to excess weight gain in children is unsubstantiated, although the cumulative effect of multiple courses of antibiotics were not evaluated in this study. Antibiotics have previously shown to be growth-promoting in lower resource settings in children with known acute or chronic illness, and these results indicate that a similar relationship may exist between antibiotic use and weight gain in community-based samples of children.

## Supplementary Information

**Additional file 1.**

**Additional file 2: Supplemental Table S1.** Baseline demographic and anthropometric characteristics among children lost and not lost to follow-up at 14 days and 6 months. (PDF 2577 kb)

## Data Availability

All data underlying this report will be made available via ClinEpiDB (www.clinepidb.org). Data will be available upon approval of a data access request made to the corresponding author or via ClinEpiDB.

## Abbreviations

IQR: Interquartile range
WHZ: Weight-for-height Z-score
HAZ: Height-for-age Z-score
WAZ: Weight-for-age Z-score
MUAC: Mid-upper arm circumference
g/kg/day: Grams per kilogram per day
SD: Standard deviation
CI: Confidence interval
