# Supplementary material for: Single-dose azithromycin for child growth in Burkina Faso: a randomized controlled trial
Source: BMC Pediatr. 2021 Mar 17;21:130. doi: 10.1186/s12887-021-02601-7 (PMC7967941; doi:10.1186/s12887-021-02601-7)
Supplement: Supplementary file 1 — Additional file 1. [file 12887_2021_2601_MOESM1_ESM.pdf]

Single-dose azithromycin for child growth in Burkina Faso: a randomized controlled trial

Ali Sié, MD PhD[1], Boubacar Coulibaly, PhD[1], Clarisse Dah, MD[1], Mamadou Bountogo, MD[1], Mamadou Ouattara, MD[1], Guillaume Compaoré, MD[1], Jessica M. Brogdon, MPH[2], William W. Godwin, MPH[2], Elodie Lebas, RN[2], Thuy Doan, MD PhD[2,3], Benjamin F. Arnold, PhD[2,3], Travis C. Porco, PhD[2,3,4], Thomas M. Lietman, MD[2,3,4], Catherine E. Oldenburg, ScD[2,3,4] for the GAMIN Study Group

Supplemental Material

CONSORT 2010 checklist of information to include when reporting a randomised trial*

Section/Topic	Item No	Checklist item	Reported on page No
**Title and abstract**			
	1a	Identification as a randomised trial in the title	1
	1b	Structured summary of trial design, methods, results, and conclusions (for specific guidance see CONSORT for abstracts)	3-4
**Introduction**			
Background and objectives	2a	Scientific background and explanation of rationale	5
	2b	Specific objectives or hypotheses	5
**Methods**			
Trial design	3a	Description of trial design (such as parallel, factorial) including allocation ratio	6
	3b	Important changes to methods after trial commencement (such as eligibility criteria), with reasons	n/a
Participants	4a	Eligibility criteria for participants	6
	4b	Settings and locations where the data were collected	6
Interventions	5	The interventions for each group with sufficient details to allow replication, including how and when they were actually administered	7
Outcomes	6a	Completely defined pre-specified primary and secondary outcome measures, including how and when they were assessed	7-8
	6b	Any changes to trial outcomes after the trial commenced, with reasons	n/a
Sample size	7a	How sample size was determined	8
	7b	When applicable, explanation of any interim analyses and stopping guidelines	n/a
Randomisation:			
Sequence generation	8a	Method used to generate the random allocation sequence	7
	8b	Type of randomisation; details of any restriction (such as blocking and block size)	7
Allocation concealment mechanism	9	Mechanism used to implement the random allocation sequence (such as sequentially numbered containers), describing any steps taken to conceal the sequence until interventions were assigned	7
Implementation	10	Who generated the random allocation sequence, who enrolled participants, and who assigned participants to interventions	7
Blinding	11a	If done, who was blinded after assignment to interventions (for example, participants, care providers, those	7

		assessing outcomes) and how	
	11b	If relevant, description of the similarity of interventions	7
Statistical methods	12a	Statistical methods used to compare groups for primary and secondary outcomes	8-9
	12b	Methods for additional analyses, such as subgroup analyses and adjusted analyses	8-9
**Results**			
Participant flow (a diagram is strongly recommended)	13a	For each group, the numbers of participants who were randomly assigned, received intended treatment, and were analysed for the primary outcome	9; Figure 1
	13b	For each group, losses and exclusions after randomisation, together with reasons	9; Figure 1
Recruitment	14a	Dates defining the periods of recruitment and follow-up	6
	14b	Why the trial ended or was stopped	n/a
Baseline data	15	A table showing baseline demographic and clinical characteristics for each group	Table 1
Numbers analysed	16	For each group, number of participants (denominator) included in each analysis and whether the analysis was by original assigned groups	Figure 1
Outcomes and estimation	17a	For each primary and secondary outcome, results for each group, and the estimated effect size and its precision (such as 95% confidence interval)	10
	17b	For binary outcomes, presentation of both absolute and relative effect sizes is recommended	10; Tables 2, 3
Ancillary analyses	18	Results of any other analyses performed, including subgroup analyses and adjusted analyses, distinguishing pre-specified from exploratory	10; Tables 2, 3
Harms	19	All important harms or unintended effects in each group (for specific guidance see CONSORT for harms)	9
**Discussion**			
Limitations	20	Trial limitations, addressing sources of potential bias, imprecision, and, if relevant, multiplicity of analyses	12
Generalisability	21	Generalisability (external validity, applicability) of the trial findings	12
Interpretation	22	Interpretation consistent with results, balancing benefits and harms, and considering other relevant evidence	10-12
**Other information**			
Registration	23	Registration number and name of trial registry	4
Protocol	24	Where the full trial protocol can be accessed, if available	n/a
Funding	25	Sources of funding and other support (such as supply of drugs), role of funders	13

*We strongly recommend reading this statement in conjunction with the CONSORT 2010 Explanation and Elaboration for important clarifications on all the items. If relevant, we also recommend reading CONSORT extensions for cluster randomised trials, non-inferiority and equivalence trials, non-pharmacological treatments, herbal interventions, and pragmatic trials. Additional extensions are forthcoming: for those and for up to date references relevant to this checklist, see www.consort-statement.org.

Gut and Azithromycin Mechanisms in Infants and Neonates in Burkina Faso (GAMIN)

Manual of Operations and Procedures
Version 2.9 July 21, 2020

Centre de Recherche en Santé de Nouna
University of Heidelberg
Francis I. Proctor Foundation, University of California, San Francisco

Catherine Oldenburg, ScD MPH
Ali Sié, MD PhD
Thomas Lietman, MD
Mamadou Bountogo, MD
Boubacar Coulibaly, PhD
Cheikh Bagagnan, MS
Alphonse Zakane, MS
Mamadou Ouattara, MD
Thierry Ouedraogo Andiyam
Fla Koueta, MD
Idrissa Kouanda
Mariam Seynou
Clarisse Dah, MD
Constantin Sow
Till Bärnighausen, MD ScD
Thuy D Doan, MD, PhD
Jeremy Keenan, MD MPH
Travis Porco, PhD MPH
Benjamin Arnold, PhD MPH
Kieran O'Brien, MPH
Elodie Lebas, RN
Jessica Brogdon, MPH
Catherine Cook, MPH
Ariana Austin, MS
Ying Lin, MSPH
William Godwin, MPH
Fanice Nyatigo, BSPH

Table of Contents

1 Chapter 1: Overview 6
1.1 Executive Summary 6
1.2 Objectives 6
1.3 Study Partners 7
1.4 Study Site 7
2 Chapter 2: Context 7
2.1 Background 7
3 Chapter 3: Study Design 8
3.1 Recruitment and Eligibility 9
3.2 Sample Size 10
3.3 Baseline Measurement 10
3.4 Randomization 11
3.5 Outcomes 11
3.6 Participant Timeline 12
4 Chapter 4: Procedures 14
4.1 Treatment 16
4.2 Field Worker Safety Assessment 16
4.3 GAMIN QOD: Rectal Swab Home Visits 16
4.4 Anthropometry Assessment 16
4.5 Whole Blood 16
4.6 Blood Smears 17
4.7 Swabs 17
4.8 Stool 17
4.9 Vital Status Assessment 17
4.10 Adverse Events 17
4.11 Summary of Sample Collections 18
5 GAMIN II Mini Study 19
5.1 Design 19
5.2 Data Capture 19

6 Chapter 6: Study Medication 19
6.1 Study Medication Description 19
6.2 Dosage Information 20
6.3 Medication Procurement 20
6.4 Medication Quality Control 20
6.5 Directly Observed Treatment 20
6.6 Adverse Events 20
6.7 Background on Infantile Hypertrophic Pyloric Stenosis in Neonates 21
6.8 Screening for IHPS 22
7 Chapter 7: Swab Specimen Collection 23
7.1 Nasopharyngeal Swabs 23
7.2 Rectal Swab Collection for Culturing/Microbiological Testing 25
8 Chapter 8: Fresh Frozen Stool Specimen Collection 26
8.1 Materials for FFS Collection 26
8.2 Collection Procedure 27
8.3 6.3 Temporary Storage and Transport Procedures 27
8.4 Processing 27
8.5 Collection and Transport 27
9 Chapter 9: Blood Specimen Collection 28
9.1 Thin and Thick Smears 28
9.1.1 Materials for Fingerstick 28
9.1.2 Fingerstick Procedure 28
9.1.3 Smears Processing and Transport 29
9.1.4 Thick and Thin Blood Smears Materials 30
9.2 Measuring Body Temperature 30
9.3 Venipuncture Blood Draw 31
9.3.1 Blood Draw Protocol 33
10 Chapter 10: Anthropometry 38
10.1 Measuring Length and Height 38
10.2 Procedure 38
10.3 Measure Length 39
10.4 Measure Standing Height 41

10.4.1 Dismantling the ShorrBoard 42
10.5 Measuring Weight 43
10.5.1 Scale Calibration 44
10.6 Measuring Mid-Upper Arm Circumference (MUAC) 44
11 Chapter 11: Vital Status 45
12 Chapter 12: Data Collection, Management, and Security 45
12.1 Data Collection 45
12.2 Data Management and Security 45
12.3 Data Quality and Monitoring 45
13 Chapter 13: Protection of Human Subjects 45
13.1 Institutional Review Board Approval 45
13.2 Informed Consent 46
13.3 Risks and Benefits of Study Procedures 46
13.4 11.3.1 Procedures 46
14 Chapter 14: Data Safety and Monitoring Committee 47
14.1 Data and Safety Monitoring Committee Charter 47
14.2 Primary Responsibilities of the DSMC 47
14.3 DSMC Membership 47
14.4 Conflicts of Interest 47
14.5 Timing and Purpose of the DSMC Meetings 48
14.6 Procedures to Ensure Confidentiality and Proper Communication 48
14.7 Statistical Monitoring Guidelines 49
15 Chapter 15: Study Team Roles and Responsibilities 50
16 References 51

ABBREVIATIONS

CRSN: Centre de Recherche en Santé de Nouna
CSPS: Centre de Santé et de Promotion Sociale
DSMC: Data Safety and Monitoring Committee
FFS: Fresh Frozen Stool
IRB: Institutional Review Board
MUAC: mid-upper arm circumference
NP swabs: nasopharyngeal swabs
PCR: polymerase chain reaction
UCSF: University of California San Francisco
WHO: World Health Organization

1 <u>Chapter 1: Overview</u>

1.1 Executive Summary

Globally, childhood mortality has shown a promising downward trend in recent years, however, many sub-Saharan countries still have relatively high child mortality rates. In previous studies within Niger, Tanzania, and Malawi, mass azithromycin treatment to children aged 1-59 months old effectively reduced all-cause childhood mortality[1]. A similar study will be conducted in Burkina Faso to replicate the results of mass azithromycin treatment.

We propose an individually randomized placebo-controlled trial alongside the MORDOR II Burkina Faso trial to evaluate the effect of a single dose of azithromycin (20 mg/kg) on potential mediators of the effect of azithromycin on all-cause mortality. Many questions surround the mechanism behind azithromycin's effect on reducing childhood mortality. Further questions exist regarding antibiotic resistance and how mass antibiotic administration can impact intestinal microflora. The goal of this study is to demonstrate the changes in the gut microbiome after antibiotic administration and to measure the growth of children after receiving a single dose of azithromycin. Additionally we will measure resistance markers, inflammatory markers, and IgA-bound bacteria throughout the study. We hypothesize that a single dose of azithromycin will lead to a significant increase in child growth and that the gut microbiome will be significantly different in children who received azithromycin compared to those who received placebo.

1.2 Objectives

1. **To determine the effect of a single dose of azithromycin for children aged 8 days-59 months on longitudinal changes in the intestinal microbiome over a 6-month period.** We hypothesize that a single dose of azithromycin will result in a significant difference in the intestinal microbiome within the treatment group compared to the placebo group after a 6-month period within children ages 8 days-59 months.

2. **To determine the effect of a single dose of azithromycin for children aged 8 days-59 months on child growth over a 6-month period.** We hypothesize that a single dose of azithromycin will increase child growth over a 6-month period in children aged 8 days-59 months.

3. **To determine the effect of a single dose of azithromycin for children aged 8 days to 59 months on the presence of macrolide genetic resistance determinants within the first two weeks post-treatment**. The investigators hypothesize that a single dose of azithromycin will increase the presence of macrolide resistance determinants over a 2-week period in children aged 8 days to 59 months.

1.3 Study Partners

CRSN and UCSF are equal partners in the production of this protocol. The University of Heidelbug is also a partner on this project. This study was designed together and will be implemented by both parties. The Bill and Melinda Gates Foundation is providing funding for this study.

1.4 Study Site

This study will be conducted in the northwestern region of Burkina Faso in the town of Nouna. Nouna is approximately 300 kilometers from the capital city of Ouagadougou. Nouna town has a population of approximately 28,000 which includes about 4,600 pre-school aged children. There is currently a hospital that serves all 7 sectors of Nouna town. We will enroll up to 1500 children presenting to the hospital.

2 Chapter 2: Context

2.1 Background

Our previous MORDOR I research demonstrated a significant reduction in all-cause child mortality after biannual mass azithromycin distribution. In three sub-Saharan Africa countries, (including Niger, Tanzania, and Malawi) mass azithromycin treatment over 2 years resulted in a 14% reduction in child mortality. Moreover, 1 in 5-6 deaths were shown to be averted within Niger alone[1]. Similar findings were demonstrated in a previous study for trachoma control in Ethiopia with mass azithromycin distribution. This study in rural Ethiopia noted a nearly 50% decrease in all-cause childhood mortality[5]. However, neither of these studies evaluated the longitudinal impact azithromycin has on the gut microbiome. The MORDOR II trial in Burkina Faso will further evaluate the efficacy of biannual azithromycin treatment. The under-5 child mortality rate in Burkina Faso is approximately 110 per 1,000 live births. Major causes of child mortality in this area are infectious mostly due to malaria, diarrhea, and upper respiratory tract infections[3]. In addition, malnutrition contributes to a high burden of child mortality and morbidity within this region as well[4]. By treating underlying conditions, the use of routine antibiotic treatment could reduce diverse health outcomes leading to morbidity and mortality. We propose to conduct this study alongside the MORDOR II trial in the town of Nouna where a majority of childhood deaths are attributable to infectious causes and malnutrition.

The World Health Organization is considering adopting the presumptive use of azithromycin and other antibiotics as a recommendation to reduce childhood mortality in areas with a high infectious disease burden[2]. Many questions remain unanswered surrounding the use of mass antibiotic treatment in areas with high child morbidity and mortality. This study will add to the current knowledge of mass azithromycin distribution from our previous MORDOR I research. We propose to evaluate how azithromycin will impact childhood growth and to assess the changes that occur in the intestinal microbiome following a single dose of azithromycin treatment. The goal is to contribute more scientific literature that could assist future guidelines regarding antibiotic use.

The role of antibiotics on child growth is unclear. Recent studies indicate that antibiotic use could impact child growth, but a previous study in Niger failed to find a statistically significant correlation between antibiotic treatment with azithromycin and stunting, underweight, or MUAC of pre-school aged children. Longitudinal studies have been recommended to further investigate the role of antibiotics on child growth[6]. Meanwhile some studies suggest antibiotics may create modifications in the gut microbiota impacting nutrient absorption and weight gain[7].We propose to measure child growth through anthropometric measurements longitudinally over a 6-month period to see if azithromycin treatment impacts child development. We hypothesize that children receiving a dose of azithromycin will have more growth and development in terms of height, weight, and mid-upper arm circumference compared to children who receive placebo.

We propose a longitudinal study designed to improve our knowledge about the changes in the intestinal microbiome following the course of a single dose of antibiotic in a setting with high childhood mortality and morbidity. More specifically, we propose to follow 450 children for a 6-month time period that are between the ages of 8 days old and 59 months old. Children in this age bracket are at the highest risk for mortality from infectious causes, and furthermore, they are at the highest risk for malnutrition. This group of children would receive the greatest benefit from this intervention. The causal changes in the microbiome are vastly understudied in regards to changes in the gut microbiome following a course of antibiotics. We hypothesize that children receiving a dose of azithromycin will have a higher prevalence of anti-pneumococcal bacterial resistance in nasopharyngeal samples, decreased bacterial diversity, and a higher likelihood of identification of bacterial resistance genes in stool and nasopharyngeal samples.

3 Chapter 3: Study Design

We propose to prospectively follow 450 children treated with azithromycin or placebo for 6 months in the town of Nouna. Our goal is to evaluate intestinal microbiome changes and child growth longitudinally. We will individually randomize eligible children to a single dose of azithromycin or placebo at the Nouna town hospital. At days 0, 14, and 180 we will collect a

series of samples to measure both outcomes. Anthropometry and vital status will be collected at each follow up visit. In addition, we will collect a blood smear at each visit, record body core temperature, collect a nasopharyngeal swab, a rectal swab, fresh frozen stool, and perform whole blood venipuncture. We will also randomly select 25 children per arm (n=50) to receive a rectal swab at days 0, 2, 4, 6,8,10, 12, 14, and 180 days after treatment. As per the Bill and Melinda Gates Foundation guidelines, all biological samples collected will be stored and made available to other investigators at the finish of the study.

GAMIN:

Azithromycin	Placebo
n=725	n=725

Supplementary rectal swab group:

Azithromycin	Placebo
n=25	n=25

3.1 Recruitment and Eligibility

3.1.1 Recruitment

Prior to enrollment in the study, mobilization will be conducted in up to four secteurs of Nouna Town (administrative districts). The mobilization will be done house-to-house within the designated secteurs. Caregivers with children under 59 months will be invited to participate. All study activities will take place at a local primary hospital. Community mobilizers will inform caregivers where to go for enrollment in the trial. Enrollment will occur at the Nouna hospital.

At the hospital, a dedicated study team member will enroll the child. The study team member will be an existing member of the enrollment site staff who will be trained for the study or an external study team member who will be placed at the site for the duration of the study. Trained staff will collect the biological specimens. The team member will verify consent from the parent or guardian and review the eligibility of the child. Following enrollment, each child will be provided with a study card and unique identification number to be entered in the tablet at each follow-up visit.

3.1.2 Eligibility

Eligible children for this study are those who are at least 8 days old but younger than 59 months old. Child must live within Nouna town and report to the local Nouna town hospital during the study period.

Inclusion Criteria

- Between 8 days and 59 months old
- Primary residence within catchment area of study site
- Available for full 6 month study
- No allergy to macrolides/azalides
- Appropriate written informed consent from at least one parent or guardian
- Able to feed orally
- No prior enrollment in the NAITRE study

Exclusion Criteria

- <8 days old or >59 months
- Primary residence outside catchment area of study site
- Not available for full 6 month study
- Known allergy to macrolides/azalides
- No written informed consent from at least one parent or guardian
- Unable to feed orally
- Prior enrollment in the NAITRE study

3.2 Sample Size

We will enroll up to 1500 children. In the main trial, the two study arms will have approximately 725/arm. In the sub-study, 25 children per arm will be randomized. This sample size is based on the primary outcome, Simpson's alpha diversity in the intestinal microbiome. We will base some statistical assumptions on preliminary data from the Nouna HDSS. For instance, we assume a 15% loss to follow-up, a baseline effective number of 9, and a standard deviation of 0.7.

3.3 Baseline Measurement

After enrollment and informed consent but prior to randomization, baseline samples will be collected. Children will undergo anthropometry (height, weight, mid-upper arm circumference), whole blood venipuncture, a finger prick for malaria blood smears, 2 NP swabs, a rectal swab and they will provide a fresh frozen stool. In addition, study staff will administer a baseline questionnaire inquiring about the child's age and sex as well as the mother's maternal age, education, number of children, and breastfeeding.

3.4 Randomization

A list of potentially eligible children will be asked a series of questions to determine their eligibility. After completion of the baseline assessment, children will be randomized to receive a single dose of directly observed oral azithromycin or placebo.

UCSF investigators will generate the randomization sequence. Children will be randomized in a 1:1 fashion to a single dose of azithromycin or placebo.

This study will be a double blind study to both the participants and the staff (both Nouna and UCSF).

After consent is obtained, the hospital worker will enter the child's information into a computer tablet or smart phone. The application "Survey Solutions" will be installed on the computer. The application will ask the hospital worker if consent has been obtained and information to determine the child's eligibility status. Once the child is determined fully eligible for the study, the application will tell the health worker which treatment letter to provide the child with. For example, the screen on the tablet may say "treat with AA" or "treat with BB". The hospital will be equipped with bottles of azithromycin donated from Pfizer that will be pre-labeled with treatment letters such as AA or BB. The application will be pre-programmed with the randomization scheme, but study staff and participants will remain blinded.

3.5 Outcomes

Primary Outcome

- **Changes in intestinal microbiome over a 6-month period (Objective 1).** Microbiome diversity will be measured at all follow-up time points.
    - **Whole Blood Venipuncture.** Whole blood venipuncture will be collected at all follow-ups. We will measure cell-free DNA and inflammatory markers such as C-reactive protein.
    - **Nasopharyngeal Swabs.** NP swabs will be collected at all follow-up time points. We will collect data on phenotypic resistance of *S. pneumoniae*, evaluate the microbiome using next-generation sequencing, and monitor genotypic resistance among various organisms.
    - **Fresh Frozen Stool**. FFS will be collected at all follow-ups. We will be looking for IgA-bound bacteria from the small intestine and evaluating inflammatory markers such as C-reactive protein.
    - **Rectal Swabs**. We will collect rectal swabs at all time points. We will monitor genotypic resistance of various organisms with targeted PCR and evaluate the microbiome using next-generation sequencing.

- **Presence of macrolide genetic resistant determinants (Objective 3).** Macrolide resistance will be measured from a sample of 50 kids within the first 2 weeks post-treatment.
    - **Rectal Swabs.** We will collect rectal swabs from 50 children at days 0, 2, 4, 6, 8, 10,12, 14 and 180 post-treatment. Macrolide resistance will be analyzed using DNA sequencing.

Secondary Outcomes

- **Child growth over a 6-month period (Objective 2).** Anthropometry measurements will be collected at all follow-up time points. Weight gain will be defined as grams per kilogram per month (g/kg/month).
    - **MUAC.** Mid-upper arm circumference will be measured at all follow-ups
    - **WAZ.** Weight will be measured at all follow-ups and weight-for-age z-scores will be calculated
    - **WHZ.** Weight and height will be used to calculate weight for height z-scores
    - **HAZ.** Height or length will be measured at all follow-ups and height-for-age z-scores will be calculated.
- **Mortality by 6-months.** Vital status will be assessed at all follow-up visits. Mortality will be defined as death within the study period. Date of death will be collected.
- **Adverse events.** Adverse events will be reported at all follow-ups.
- **Malaria.** Thin and thick smears will be collected at all follow-up time points to determine malaria status. Additionally, we will measure each child's core body temperature.
- **PERMANOVA L-1, L-2**. Norm distance on bacterial reads (intestinal) for 50 children within the first 2 weeks post-treatment.
- **Campylobacter**. Changes in normalized reads for Campylobacter species will be evaluated using DNA-seq from rectal swabs of 50 kids collected within the first 2 weeks after treatment.
- **Resistome**. Chao1 total resistance gene determinant richness using DNA-sequencing from rectal swabs of 50 kids collected within 2 weeks after treatment.

3.6 Participant Timeline

Figure 1. Participant Timeline and Flow

Children Enrolled in the Study

Eligibility Criteria:

- Between 8 days and 59 months old
- Primary residence within catchment area of study site
- Available for full 6 month study
- No allergy to macrolides/azalides
- Appropriate written informed consent from at least one parent or guardian

Baseline assessment:

- Baseline questionnaire (feeding practices, household survey, maternal survey)
- Anthropometry (height, weight, MUAC)
- Malaria (thin and thick smears) and temperature
- Whole Blood Venipuncture
- NP Swab
- Fresh Frozen Stool
- Rectal Swab

Randomized

Randomized to single dose azithromycin | Randomized to placebo

50 randomly selected children (25 per arm) will have an additional rectal swab collected every other day post-treatment. This will be done as a home visit.

Day 0, 2, 4, 6, 8 , 10, 12, 14, and 180 : Rectal swab

Children <28 days old at time of treatment

Day 7, 14, 21: IHPS and Adverse Event Follow-up

All Participants

Day 14:

- Anthropometry (height, weight, MUAC)
- Adverse Events
- Vital Status
- Malaria (thin and thick smears) and temperature
- Whole Blood Venipuncture
- NP Swab
- Fresh Frozen Stool
- Rectal Swab

Day 180 (Includes window of 120-210 days):

- Anthropometry (height, weight, MUAC)
- Vital Status
- Malaria (thin and thick smears) and temperature
- Whole Blood Venipuncture
- NP Swab
- Fresh Frozen Stool
- Rectal Swab

4 Chapter 4: Procedures

Table 1 provides an overview of all study procedures. The eligibility assessment, enrollment, and randomization are explained in Chapter 3. See the appendix for all study forms described in the table.

Table 1: Overview of Procedures

Day (since treatment)	Activity	Study Form
Day 0, 2,4,6,8,10,12, 14, 180	• Treatment<br>• Rectal swab for 50 randomly selected children	• Form 1: Eligibility<br>• Form 2: Baseline<br>• Form 6: Treatment<br>• Form 8: Rectal Swab Cohort
0 (Baseline)	• Eligibility Assessment<br>• Enrollment<br>• Randomization<br>• Anthropometry<br>• Whole Blood<br>• Blood Smear<br>• Temperature<br>• NP swab<br>• Rectal Swab<br>• Fresh Frozen Stool<br>• Treatment	• Form 1: Eligibility<br>• Form 2: Baseline<br>• Form 4: Exams<br>• Form 6: Treatment
Day 7	• IHPS follow-up on neonates	• Form 7: IHPS
Day 14	• Anthropometry<br>• Whole Blood<br>• Blood Smear<br>• Temperature<br>• NP swab<br>• Rectal Swab<br>• Fresh Frozen Stool<br>• IHPS follow-up on neonates<br>• Vital Status<br>• Adverse Events survey	• Form 3: Follow-up Registration<br>• Form 4: Exams<br>• Form 7: Vital Status,AE, IHPS
Day 21	IHPS follow-up on neonates	• Form 7: IHPS
Day 180 *(primary outcome)*<br><br>**Includes window of 120-210 days*	• Anthropometry<br>• Whole Blood<br>• Blood Smear<br>• Temperature<br>• NP swab<br>• Rectal Swab<br>• Fresh Frozen Stool<br>• Vital Status	• Form 3: Follow-up Registration<br>• Form 4: Exams<br>• Form 7: Vital Status,AE, IHPS

4.1 Treatment

Details on the study medication are provided in Chapter 5. No child will be treated before 8 days of life or after 59 months. Once the child is treated, a team member will record if the treatment was given or if the child did not receive treatment. If the child was not treated, the team member will provide the reason why the child was not treated.

4.2 Field Worker Safety Assessment

3 weekly home visits will be performed by a field worker for any participants younger than 28 days old at the time of treatment. The visits will be performed 7,14, and 21 days after treatment. Specifically, the team will be asking a series of questions to the parent or guardian in an attempt to screen for infantile pyloric stenosis. See Chapter 6 for more information on IHPS. The field worker will ask if the child has vomited since treatment, if the vomit was projectile, and if the child is gaining or losing weight. Those who answer affirmatively will be referred to the study pediatrician for closer examination and follow-up.

All caregivers will be given a pamphlet in the local language explaining IHPS in detail. Contact information and instructions on what to do if the child presents symptoms will also be provided.

4.3 GAMIN QOD: Rectal Swab Home Visits

50 children enrolled in the study will be randomly selected to provide additional rectal swabs at days 0, 2, 4, 6, 8, 10, 12, 14, and 180 days after treatment. We will select 25 children per study arm and conduct a home-visit for these given days.

4.4 Anthropometry Assessment

We will record every participant's weight, length/height, and mid-upper arm circumference at baseline, day 14, and day 180 after treatment. Weight will be measured using the SECA scale. Length/height will be measured using a Shorrboard to the nearest cm. See chapter 9 for the anthropometric measurements protocol.

4.5 Whole Blood

Every participant will undergo a blood drawl procedure at baseline, day 14, and day 180 after treatment. Venipuncture will be done by a trained professional using a sterile butterfly-winged needle. See chapter 8 for the venipuncture protocol.

4.6 Blood Smears

Thick and thin blood smears will be collected for every enrolled child at baseline, day 14, and day 180 after treatment. The child's finger will be pricked with a lancet by a trained professional. We will use the smears to determine the child's malaria status. Additionally, we will record the child's core body temperature. If the child has a fever, we will administer a rapid diagnostic test. We will use CareStart^{TM} Malaria Pf (HRP2) Ag RDT or OnSite^{TM} Malaria Pf/Pan Ag Rapid Test depending on availabilityl If the child is positive for malaria, we will give the child a referral to the health center. See chapter 8 for more on the blood protocol.

4.7 Swabs

Nasopharyngeal and rectal swabs will be collected from each participant at baseline, day 14, and day 180 after treatment. Flocked pediatric swabs will be used for the NP collection. See chapter 6 for more information on the swab protocol.

4.8 Stool

We will collect fresh frozen stool from participants at each visit. Infants will be given a diaper to produce the sample while older children will be provided with miniature potties. The potties will have plastic bags placed around the opening to catch the sample. More details about stool collection are found in chapter 8. The potties will be sanitized after each use.

At baseline, children that cannot produce the sample will be asked to stay at the hospital for up to 2 hours. At the follow-up visits, the field team will schedule appointments for the child to come back and produce the sample. Alternatively, the field team will go to the child's home to obtain the sample. The sample will then be placed in RTIC coolers and immediately transported back to the laboratory freezer.

4.9 Vital Status Assessment

A vital status assessment will be conducted at each follow-up visit (days 14 and 180). Field workers will record on the tablet if the child is alive and present, alive but not present, alive but refuses to continue, died, moved, or unknown. Vital status will also be conducted during the neonatal follow-up visits at days 7, 14, and 21 after treatment.

4.10 Adverse Events

At the 14-day follow-up visit, field workers will conduct a survey of adverse events, including if the child had any of the following symptoms:

- Fever
- Diarrhea
- Vomiting
- Abdominal pain
- Skin rash
- Constipation

The field worker will also assess if the caregiver had sought care for the child since the last time they spoke to the study team, and if so, what the reason for the health care visit and if the child was hospitalized.

4.11 Summary of Sample Collections

Summary of laboratory and other assessments in Nouna longitudinal study

**Organism/Outcome**	**Test**	**Sample**	**Time Point (Days)**			**Processing Site**
			**0**	**14**	**180**	
Malaria	Thick and Thin Blood Smears	Finger stick	✓	✓	✓	Nouna
Phenotypic resistance: *S. pneumoniae*	Kirby Bauer (or Etest)	NP swabs	✓	✓	✓	Nouna
Genotypic resistance: *E. coli*, *S. pneumoniae*, *S. aureus*, *Campylobacter* spp*Klebsiella* spp	Targeted PCR: *erm*, *mef*, *mph*	Rectal and NP swabs	✓	✓	✓	UCSF
Microbiome	Next-generation sequencing	Rectal and NP swabs	✓	✓	✓	UCSF
Inflammatory markers	C-reactive protein	Whole blood and frozen stool	✓	✓	✓	Nouna and/or Gates labs
IgA-bound bacteria from small intestine	BugFACS	Whole blood and frozen stool	✓	✓	✓	Gates labs
Nutritional status	Anthropometric measurements	Weight, height, MUAC	✓	✓	✓	Nouna
Vital status	Mortality, hospitalization	Caregiver questionnaire		✓	✓	Nouna

5 GAMIN II Mini Study

The study team will conduct a mini "GAMIN II" study in late summer/early fall of 2020. The GAMIN II study will enroll approximately 450 children meeting the same eligibility criteria as the original GAMIN study. Participants from the original GAMIN study will be ineligible to participate in the GAMIN II mini study.

5.1 Design

The GAMIN II study will involve 3 study visits at baseline, 14 days, and 6 months.

At baseline, children 8 days-59 months old will complete a rapid diagnostic test for malaria, have their temperature taken, and produce a fresh frozen stool sample. The children will also receive a single oral dose of azithromycin or placebo (with a 1:1 randomization).

At the day 14 visit children will be asked again to complete an RDT, take their temperature, and provide a fresh frozen stool sample. Chapters 8 and 9 describe the procedures in detail.

At the 6 month visit, children will only be asked to collect a fresh frozen stool sample.

The child's vital status will be collected at each visit.

5.2 Data Capture

The GAMIN II study will utilize CommCare for electronic data capture. Each child will randomly be assigned an identification number. The identification number will contain the letter "L" and 6 digits.

6 Chapter 6: Study Medication

Enrolled participants will be offered weight-based, directly observed, oral suspension azithromycin or placebo. Adverse events will be monitored.

6.1 Study Medication Description

Zithromax® for oral suspension is supplied in bottles containing azithromycin dehydrate powder equivalent to 1200mg per bottle and the following inactive ingredients: sucrose; tribasic anhydrous sodium phosphate; hydroxypropyl cellulose; xanthan gum; FD&C Red #40; and flavoring including spray dried artificial cherry, crème de vanilla, and banana. After constitution, a 5mL suspension contains 200mg of azithromycin.

6.2 Dosage Information

All children will receive a single dose of azithromycin or placebo. Dosage will be weight-based or height-based. Dosing will follow the WHO recommendations for treatment of active trachoma:

- Single dose of 20mg/kg in children (up to the maximum adult dose of 1g)

Children who are allergic to macrolides/azalides will be excluded from this study.

6.3 Medication Procurement

Study medication will be procured in the United States from Pfizer, Inc and shipped to the study site. All medications will be prepared as an oral suspension. Participants will not incur any costs associated with the study or the study medication.

6.4 Medication Quality Control

The study medication will be stored at the Centre de Recherche en Sante de Nouna in a temperature monitored storage room. The Nouna team and site staff will regularly check the expiration dates and all expired medication will be discarded appropriately. The study coordinator will ensure the hospital has adequate stock of study medication.

6.5 Directly Observed Treatment

Each child will take the oral suspension of azithromycin or placebo under direct observation by a hospital staff member. All doses will be electronically recorded on the study tablet.

6.6 Adverse Events

Azithromycin is generally well tolerated. The most common side effects include abdominal pain, diarrhea, and vomiting. These symptoms occur in less than one in twenty people who receive it. Additional side effects are rare, but include allergic reactions and abnormal liver function. Diarrhea due to *Clostridium difficile* has been reported in uncommon cases.

At each monitoring visit, staff members will screen all enrolled children for any adverse events. Parents or guardians of the participants will be advised to report any adverse events by phone or in person. Study staff will inquire about the child's experience of adverse events during each follow-up visit and record if the child has had any of the following symptoms:

- Fever
- Diarrhea
- Vomiting
- Skin rash
- Constipation
- Abdominal Pain

The study team will also inquire whether the parent or guardian sought care for the child since the last monitoring visit. If so, the team will find out what the reason for the health care visit was and if the child was hospitalized.

The definition of serious adverse events will include death, hospitalization, or any other life-threatening situation. Within 24 hours, serious adverse events must be reported to the Medical Monitor. The study team member conducting the follow-up visit must email Dr. Ali Sie, the Director of CRSN, who will immediately email the Medical Monitor and UCSF team. The Medical Monitor will evaluate the adverse event and determine if the event could be reasonably related to azithromycin and report the results of this determination to the investigators and the DSMC. All information on adverse events, both serious and non-serious, will be kept on data collection forms through the mobile application.

6.7 Background on Infantile Hypertrophic Pyloric Stenosis in Neonates

IHPS is a condition in which the pylorus of the stomach becomes thickened, resulting in gastric outlet obstruction.[8] In developed countries, the incidence of IHPS is approximately 2 per 1,000 infants in the general population. While surgery is generally curative, the condition is lethal in the absence of surgery. The cause of IHPS is unknown and likely complex, and both genetic[9,10] and environmental[11,12] factors are thought to contribute to its development. Male neonates are disproportionately affected by IHPS, with a 4-5:1 male to female ratio[.8,10,11] Other risk factors for IHPS include prone sleeping position, bottle/formula feeding,[12,14,15] preterm birth[11,12] cesarean section delivery[11,12] and birth order.[8,11-13] IHPS incidence appears to be decreasing over time, which some have attributed to public health interventions promoting supine sleeping position and exclusive breastfeeding.[16,17]

In addition to genetic and environmental factors, erythromycin is associated with increased risk of infantile hypertrophic pyloric stenosis (IHPS).[18] Given that azithromycin is a related compound, there is some concern that azithromycin may lead to increased risk of IHPS in neonates. Limited evidence exists of the risk of IHPS among neonates treated with azithromycin.[19] Two randomized controlled trials have assessed the use of intravenous azithromycin for prevention of bronchopulmonary dysplasia (BPD) in low birthweight infants (<1,250g) compared to placebo within 72 hours of birth.[20,21] Of 263 neonates enrolled in the two studies (N=130 receiving IV azithromycin), no cases of IHPS were reported. A third non-placebo controlled randomized trial of azithromycin prophylaxis for BPD in premature neonates additionally reported no cases of IHPS among 53 neonates receiving azithromycin.[22] An observational study of 58 infants receiving azithromycin following exposure to a healthcare worker with pertussis did not identify any cases of IHPS.[23] The largest study of azithromycin exposure in neonates is a retrospective cohort of more than one million infants in the TRICARE Management Activity military health system, which reported an overall IHPS rate of 2.3 per 1,000 (95% CI 2.2 to 2.4) among children in the first 90 days of life.[24] Of 4,875 infants prescribed azithromycin, there were 8 cases IHPS, 3 of which occurred when azithromycin was prescribed during the first 14 days of life and 5 of which during the first 15-42 days of life. Overall, there was no significant difference in IHPS rate in infants treated with azithromycin

versus IHPS rates among infants who had not received an antibiotic during the first 90 days of life were not presented by time since birth. Table 2 lists the rates of IHPS following azithromycin, erythromycin, or cephalexin prescription.

Table 3: Unadjusted rate of IHPS in infants with oral azithromycin, per 1,000

	Azithromycin	Erythromycin	Cephalexin
0-14 days[1]			2.2 (0.05-
15-42 days[1]	20.3 (4.2-59.2)	30.9 (14.1-58.7)	12.2)
43-90 days[1]	6.9 (2.2-16.1)	9.3 (3.0-21.8)	3.1 (1.0-7.2)
Overall	0 (0-0.9)	2.8 (0.6-8.1)	0 (0-1.6)
	1.6 (0.7-3.2)	8.9 (5.2-14.3)	1.4 (0.5-3.0)

There is relatively little evidence of the epidemiology of IHPS in sub-Saharan Africa. A study in Nigeria documented 57 cases of IHPS from 1978-2008 at a university teaching hospital, with only a single case from 2003-2008.[17] A study of 102 cases of IHPS over a 5-year period in Tanzania documented a 4.9% mortality rate despite surgical intervention.26 The risk of mortality was higher in infants under 2 weeks of age and those with delayed presentation to care. A retrospective study at a tertiary hospital in Ethiopia found that 12.9 per 1,000 admissions were due to IHPS, with a 3.3% mortality rate.[13]In Ouagadougou, an unpublished case series of 32 infants treated for IHPS showed a 6.3% mortality rate, which was attributed to late presentation due to the infant traveling from far outside the city.

6.8 Screening for IHPS

Field workers will conduct home visits with caregivers every week for 3 weeks following treatment of any neonate < 28 days old. Any child suspected of having IHPS will be immediately referred to the study pediatrician for evaluation. Evaluation will include ultrasonography and physical exam. Physical exam will include assessment of the pyloric olive (a thickened and elongated pylorus). Any child with a positive physical exam for a pyloric olive will be immediately transferred to the pediatric surgical unit in Ouagadougou or Bobo.

Images will be taken including the longitudinal pylorus with canal length measurement, transverse pylorus with muscle thickness measurement, and the relationship of the pylorus to the gallbladder. IHPS will be strongly suspected in infants with a permanently closed pylorus and exaggerated, retrograde gastric peristalsis. Diagnostic measurements include pyloric muscle thickness (diameter of a single muscular wall on a transverse image) >3 mm, length (longitudinal measurement) >15 mm, pyloric volume >1.5 cc, and pyloric transverse diameter >13 mm.[25] The child should be placed with their right side down, and the pylorus watched to determine if it opens. Small infants not below the pathologic limits with a permanently closed pylorus will also

be considered for further workup.[25] Any child with a pyloric muscle thickness >3 mm will be immediately transferred to the pediatric surgical unit in Ouagadougou.

All children will be assessed for electrolyte disturbance and dehydration, and rehydration and correction of electrolyte disturbance will occur prior to surgery.

Any child participated in the study that is suspected of IHPS will receive free transportation provided by the study to a tertiary hospital for clinical evaluation. If the participant is clinically diagnosed, all medical and transportation costs incurred by the child will be fully covered by the study.

7 Chapter 7: Swab Specimen Collection

7.1 Nasopharyngeal Swabs

Nasopharyngeal swabs will be collected from all study participants at 3 different time points (day 0, day 14, day 180).

The examiner will:

1. Place a pediatric flocked swab with a nylon tip through the right nostril and down the nasopharynx of each participant. Note that if the swab is not perpendicular to the frontal plane of the face, it is likely not in the inferior turbinate.

2. Once you reach the nasopharynx, rotate the swab 180° as you remove the swab from the nose.

3. Place the swab in a tube containing 1.0 mL DNA/RNA shield media by Zymo or STGG (skim milk, tryptone, glucose, and glycerin) media, cut the handle off using sterile scissors, and close the cap of the tube with the swab immersed.

4. The nasopharyngeal swab samples in STGG will initially be stored in the field at 4°C using an insulated storage bag with Fisher brand ice gel packs, and then transferred to -20°C. The nasopharyngeal swab samples in DNA/RNA shield media will be stored in ambient temperature in the field. Then transferred to a refrigerator or freezer.

5. The scissors used to cut calcium alginate swabs will be sterilized with alcohol pads or cleaned with bleach wipes between participants. When collecting specimens in DNA/RNA shield, scissors will be cleaned between participants - first with bleach wipes, and then with alcohol pads.

Do not attempt to collect the NP swab if you are not successful after three attempts.

Nasopharyngeal swabs will be stored in DNA/RNA shield media by Zymo or STGG media, and standard microbiologic techniques will be used to isolate S. pneumoniae and test for resistance to erythromycin, tetracycline, sulfa-trimethoprine, oxicillin, and clindamycin. Resistant isolates will be assessed for the most common genetic resistant determinants (ermB and mefA) using a PCR-based assay. Serotype will be assessed using a nested PCR reaction for the most common serotypes, followed by the Quellung reaction for any untyped isolates.

7.1.1 Materials for Swab Collection for Resistance Testing

Swabs
NP specimens will be collected using sterile, individually-wrapped pediatric flocked swabs with a plastic swab shaft (manufactured by Copan). Nasal sites will be swabbed with a sterile, Dacron polyester-tipped swab with a plastic shaft (manufactured by Fisherbrand).
Sample Tubes
All field samples for DNA testing will be collected into sterile 2.0ml microcentrifuge tubes, manufactured by Sarstedt®. (DNA-free tubes will be used for collection in DNA/RNA shield.)
Cooler Bags with Frozen Ice Packs
Insulated cooler bags will be used to carry samples to and from the field. In addition, frozen gel ice packs designed to thaw slowly will be used to maintain the temperature in the cooler bags during transport.

-80°C Freezer
A standard -80°C freezer located at the CRSN microbiology laboratory will be used for the storage and freezing of ice packs and samples. This freezer is kept in a locked room on the grounds of the CRSN, which is under 24-hour security guard supervision.

7.1.2 Protocol for Tubing and Handling of Samples

The tubing and handling protocol must be carefully followed in order to prevent contamination and ensure the safe transport of the samples back to the CRSN microbiology laboratory and/or to the US for processing. The person in charge of labeling, tubing, arranging, and handling the samples needs to perform this task in the most orderly and attentive manner.

1. Both hands of the tuber should be gloved at all times. The tuber's gloves only need to be changed when any potential contamination of the gloves occurs. The tuber opens the capped, hinged lid of a microcentrifuge tube, which has been labeled with the participant’s random identification number.
2. The swab is inserted by the examiner into the microcentrifuge tube held by the tuber. The swab shaft should only be inserted until the swab head is fully in the tube. The tuber will cut the swab shaft with sterile scissors.
3. The tuber should screw the cap of the microcentrifuge tube tightly, flick the tube to mix the sample with the media (for tubes with DNA/RNA shield media), and place it in the sample collection box, located in the cooler bag filled with frozen ice packs. The flap of the cooler bag should be closed between each patient. The cooler bag should be in as cool a place as possible in the field, in a shaded area out of the sun.

Upon returning from the field each day, the samples in STGG will be immediately taken to a local health center and stored in a commercial -80°C freezer, reserved solely for storage of specimens and ice packs. All samples will be in sample boxes, labeled with the village name for easy future identification.

7.2 Rectal Swab Collection for Culturing/Microbiological Testing

Rectal swabs are collected at baseline, day 14, and day 180. A small cohort of 50 randomly selected children will receive an additional rectal swab on days 0, 2, 4, 6,8,10, 12, and 180 post treatment. The swabs collected for the small cohort will be done as a home visit.

The test will require that the child's parent and examiners work together to obtain a good sample. Is it important to describe the test to the parent so that they can best assist with keeping the child still during the procedure, if necessary.

In place of stool specimens, rectal swabs can be collected in the following way:

1. Put on a clean pair of gloves.
2. Partially open the fecal swab package and remove the top section of the collection vial (this can be discarded).
3. Position the child:
   - Lie the child on his/her back, hold legs in the air (it is useful to have assistance).
   - Or have the child lay on his/her stomach across the mother/guardian's lap
4. Remove the swab from the package. Take care that the cotton tip is not touched. If it is touched, throw the swab away and begin with a new one.
5. Insert the tip of the swab into the child's anus only as far as needed to contact fecal material (1-3cm) and rotate 180 degrees. The tip should be a brownish color when removed.
6. Place swab into the preservative in the collection tube. Make sure the swab is fully submerged in the liquid preservative and then break the swab off using the pre-scored breaking point.
7. Screw the cap back on the tube and make sure that it's tightened. Wrap the area where the cap meets the tube with Parafilm to ensure that the sample will not leak, and then place the tube into the appropriate sample box.
   - If the swab cannot be broken off while the tip is fully submerged in the liquid, try twirling the swab in the liquid first (to release the contents of the sample into the preservative) before breaking it off. Avoid rubbing the sample on the tip of the swab off on the side of the tube where there is no liquid.
8. Place a random number label on the collection tube.
9. Place the tube the rectal swab container.
10. **Swab storage for Genetic analysis:** Store samples at room temperature. According to the manufacturer, the preservative in the tube will preserve DNA for 5 months at room temperature (7 days for RNA), and thereafter can be frozen (-20°C or -80°C) for long-term storage.

5.2.1 Materials for Rectal Swab Collection

Swab

An individually-wrapped Copan flocked swab with a plastic shaft will be used to collect the rectal swab and then placed into a Stool Nucleic Acid Collection and Transport Tube containing Norgen Stool Preservative or Amies Transport Medium.

Sample Tube with Media

The specimen will be in a sterile Stool Nucleic Acid Collection and Transport Tube containing Norgen Stool Preservative or Amies Transport Medium with a cap that will be tightened firmly.

5.2.2 Quality Control Measures for Specimen Collection

Negative Field Controls
Negative field control swabs for NP and stool will be taken in each community to assess for contamination: one control swab each (NP and stool/rectal) are taken before specimen collection begins in a community; and another (NP and stool/rectal) upon completion of specimen collection.

1. For each negative field control, the examiner will open a new swab as described above.
2. Wave the swab in the air, without making contact with anyone/anything.
3. Tube the swab in media, as described above.

8 Chapter 8: Fresh Frozen Stool Specimen Collection

Fresh frozen stool will be collected to evaluate IgA-bound bacteria and inflammatory markers from the small intestine. Children unable to produce a stool sample at the time of the follow-up visits will be asked to come back during a scheduled appointment. Children unable to produce a stool sample at the time of the baseline visit will be asked to stay at the health facility for 2 hours in an attempt to collect the sample prior to the treatment administration.

8.1 Materials for FFS Collection

- Stool specimen (10-20 grams or ml)
- Pre-printed PID labels (4 plus 1 extra)
- Plastic disposable transfer pipette for liquid stools
- Cotton tipped wooden stick
- Wide-mouthed plastic container suitable for collecting stools
- Wooden spatula
- Frozen ice packs
- Cold box
- Tube rack
- Disposable latex gloves
- Disposable diaper

- Sealable plastic bags
- Plastic spoon
- Pen
- Stool Field Collection Form (SFC)

8.2 Collection Procedure

Stool sample will be collected three times. Once before the treatment administration, once 14 days after the treatment administration and the last time 180 days after treatment administration.

The guardian will be asked to come to the hospital for the child to be enrolled in the study, examined, and treated. The guardian will be asked to come back for each visit to the hospital (Baseline, Day 14 and day 180). The guardian will be reminded the day before that she/he has to come to the hospital for examination. The stool sample will be collected on site, not at the child's home.

The mother / participant's primary caretaker/ participant will be provided with the labeled stool container, diaper (for infants), gloves, plastic spoon, and 2 plastic bags when she arrives at the station. The caretaker will be instructed to use the plastic spoon to collect 3-4 spoons of stool within 20 minutes of defecation and place it in the stool container, close the lid tightly, and place the container in the plastic bag.

8.3 6.3 Temporary Storage and Transport Procedures

Instruct the caretaker to hand the plastic bag with the stool to the CRSN personnel immediately after collection (maximum time: 20 minutes). Collect the stool specimen as soon as possible and document if the sample was in a cold environment on the requisition CRF. Also document if specimen is acceptable (estimated quantity, lid closed, and no leakage).

8.4 Processing

Label the original stool container with SID labels. Write the date of collection (DD/MM/YY) and time of collection (hh:mm; 24 hour time scale) on the label.

8.5 Collection and Transport

Initial collection of fecal samples should be made in a suitable sterile container after which smaller aliquots of fecal material should be transferred by the field worker (within 20 minutes of defecation) into pre-labeled, sterile 2ml cryo-safe tubes. Tube labels should minimally include the Participant's Sample ID (SID) and the date of collection, or as specified by the BEED manual of procedures.

Wearing clean, disposable latex gloves, fill each 2 ml cryo-vial approximately one-half to two-thirds full using a sterile spatula and cap tightly. Do not add any buffers, preservatives or additives to the sample. [Note that use of screw cap vials and not overfilling them minimizes the

potential for cross-contamination of samples during transport and storage].Immediately place capped vials into liquid nitrogen pre-charged ‘dry shippers’ (for transport back to the laboratory. Specimens should be transferred to dry shippers within 20 minutes of defecation. [Note: dry shippers can be reused between charges so long as they are checked each morning following the manufacturer’s instructions to ensure sufficient liquid nitrogen is present to complete the intended sampling needs for the day]. Upon return to the laboratory, empty the vials from the dry shipper into a bucket of dry ice to prevent thawing while sorting and transferring the vials to 9x9 freezer boxes for longer term storage and transport.

9 Chapter 9: Blood Specimen Collection

9.1 Thin and Thick Smears

9.1.1 *Materials for Fingerstick*

Thin and thick blood smears will be read using standard microbiological techniques at the CRSN laboratory.

Fingerprick
Gloves
Disposable lancets
Alcohol wipes
Cotton balls
Gauze
10% household bleach or 4% chlorhexidine solution to clean spills
Absorbent material for spills
Sharps container

9.1.2 Fingerstick Procedure

Inform the mother that her child’s finger will be pricked to obtain blood to test for malaria and anemia. Describe the finger prick procedure, reassure her, and answer all questions. The blood specimen should be collected as described below to minimize the discomfort of the child and to ensure sufficient blood volume collection.

A finger stick of capillary blood will be collected for thin and thick blood smears to assess for malaria. Blood will be collected by a gloved health worker using aseptic technique. Gloves will be changed between each participant. The fingerprick or heelstick site will be disinfected using a 70% isopropyl alcohol swab.

1. Prepare the disposable lancet. Use a NEW disposable lancet for each child. **Do not** re-use lancets!

2. The recorder will scan the child's QR code, and place a random number sticker on the TropBio filter paper and the (right edge of the) slide.
3. Position the child for the finger stick. Make sure that the child's right hand is warm and relaxed. Hold the child's thumb, middle, or ring finger on his/her right hand (from the top of the knuckle to the tip of the finger) between your left thumb and finger and disinfect in small outward circles with an individually packaged alcohol wipe.
4. After the alcohol dries, use the thumb to lightly press the child's thumb or finger from the top of the knuckle towards the fingertip to stimulate blood flow towards the sampling point (puncture site). For the best blood flow and least pain, prick the side of the thumb/fingertip, not the center. While applying light pressure towards the thumb/fingertip, hold the lancing device in your hand and prick the thumb/finger. If the finger prick is performed properly, a single prick should be sufficient to collect the required amount of blood.
5. Allow the blood to ooze out. Wipe away the first 2 or 3 drops of blood with gauze. If necessary, re-apply light to moderate pressure towards the thumb/fingertip (approximately 1 cm behind the site of the finger prick) until another drop of blood appears.
   Note: **Do not** squeeze forcefully. Avoid "milking" as it may dilute the blood with tissue plasma.

9.1.3 <u>Smears Processing and Transport</u>

1. Label the slide with a random number sticker.
2. For the thick blood smear:
   a. Place a drop of blood in the center (1 cm from the edge of the slide) of a clean, dust-free, and grease-free slide.
   b. Spread the drop of blood evenly with a disposable wooden applicator or with another clean slide into a circle with a diameter of 1 cm.
   c. The blood smear should be about 1cm away from the edge of the slide. The correct thickness of a thick blood smear is one through which newsprint is barely visible when the blood is still wet.
3. For the thin blood smear:
   a. Place a smaller drop of blood on the slide.
   b. Using another slide angled at 45°, create a feathered edge before reaching the other end of the slide.
4. Allow the blood smears to air dry flat. Do not heat the slides, as this will damage the parasites. Be sure to protect the slide from dust and insects. Do not refrigerate slides, as this may cause the smears to detach from the slide during the staining procedure.
5. When dry, place the thick and thin blood smears into the slide box.
6. Smears will be transported at room temperature each day to a diagnostic facility near the study area.
7. Within 24 hours of thick and thin blood smear collection, the smears will be stained with 2% Giemsa stain for 30 minutes. (The thin smear will be fixed by submerging it in 100% methanol for 30 seconds and then let it air dry for 1-2 minutes prior to the Giemsa stain.)
8. Parasite density will be measured by a masked reader using a microscope at the diagnostic facility.

Smears will be stored at room temperature.

9.1.4 Thick and Thin Blood Smears Materials

Glass slides
Slide box
Rolls of random number stickers

9.2 Measuring Body Temperature

Measuring temperature:

Place a clean lens filter (cover) over the end of the device. Press the Start button and push the «I/O» button to clear the previous reading. During the internal self-check, the display shows all segments. Then the last temperature taken will be displayed together with «MEM». Wait for the ready signal to beep and the ready symbol in the display. Fit the probe snuggly into the canal of the child's right ear, then push and release the Start button. If the probe has been securely inserted into the ear canal during the complete measuring process, a long beep will signal the end of the measuring process. The thermometer detects that an accurate temperature measurement has been taken. The result is shown on the display.

If the probe has not been constantly placed in a stable position in the ear canal, a sequence of short beeps will sound, the «ExacTemp» light will go out and the display will show an error message («POS» = position error).

For the next measurement, eject the used lens filter (push the ejector) and put on a new, clean lens filter. Clear the display by pushing the «I/O» button once. Wait for the ready signal. Fit the probe snuggly into the ear canal, then push and release the Start button

The ear thermometer turns off automatically after 60 seconds of inactivity. The device can also be turned off by pressing the «i/O» button for at least three seconds.

Collect used lens filters to be cleaned at the end of the day.

9.3 Venipuncture Blood Draw

Protection of Examiner and Study Participant

Prior to examinations at the blood station and the swab station, the examiner and tuber must be gloved. The examiner will put latex gloves on both of his/her hands prior to touching the participant and a new pair of gloves will be used for each participant in order to avoid transmitting infection between participants. Purell® Instant Hand Sanitizer will be available for hand sanitization when needed.

It is important to handle all blood specimens with care

to minimize risk of infection

Wear gloves. New gloves must be worn for each child.

Clean spills. In the event of a blood spill or splash, clean immediately with approved disinfectant (10% bleach or chlorhexidine solution) and wipe with absorbent material.

Disposal of sharps. All lancets must be disposed of properly in sharps containers.

No food. Food and drink are not allowed at the blood collection station.

Determination of the maximum allowable blood draw volume
See the chart below in order to determine the maximum allowable blood draw volume from children and infants, based on body weight.

**Body wt in kg**	**Max drawn in one blood draw**
	2.5% of total blood volume
1 kg	2.5 ml
2 kg	4.5 ml
3 kg	6 ml
4 kg	8 ml
5 kg	10 ml
6 kg	12 ml
7 kg	14 ml
8 kg	16 ml
9 kg	18 ml
10 kg	20 ml
11 thru 15 kg	22 - 30 ml
16 thru 20 kg	32 - 40 ml
21 thru 25 kg	42 - 50 ml
26 thru 30 kg	52 - 60 ml
31 thru 35 kg	62 - 70 ml
36 thru 40 kg	72 - 80 ml
41 thru 45 kg	82 - 90 ml
46 thru 50 kg	92 - 100 ml
Greater than 50 kg	100 ml

Citation for above chart: Seattle Children's Hospital Powered by Mayo Medical Laboratories. "Maximum Allowable Blood Draw from Infants."
https://seattlechildrenslab.testcatalog.org/show/1000721-1

Cell-Free Plasma extraction
In order to prepare whole blood for downstream molecular biology techniques, plasma with an absence of lysed blood cells must be prepared from the whole blood. Plasma is prepared as follows, according to the Streck tube protocols.

1- Centrifuge whole blood at 1,600 x g for 10 minutes at room temperature (20-25ºC).
2- Carefully pipette the upper plasma layer without disturbing the lower blood cell layer or pipetting up any red material. Transfer the plasma to a separate conical-bottom tube. *Discard the remaining blood cells and tube in Biohazardous Waste.
3- Centrifuge the plasma at 16,000 x g for 10 minutes.

4- Carefully pipette the upper plasma layer (translucent), avoiding the opaque lower layer, and aliquot it into labeled cryo-tubes, with 200uL in each tube.
   *Discard the remaining fluid and tube in Biohazardous Waste.
5- Store extracted plasma at ≤ -80ºC for future use in molecular biology protocols.

9.3.1 Blood Draw Protocol

Identification

Identify the child by scanning her/his study identity card.

Verify the identity of the child with the mother/guardian of the child: check the name, study identification number, sex, age and the name of the mother and father of the child.

Supplies

2 chairs
1 examination table
1 pillow
1 Cotton ball
1 Alcohol wipes
1 Wing needle 23G with tubing and holder
1 needle container
1 trash bag
1 small bandage
1 pair of gloves
1 tube to collect blood (streck brand Cell Free DNA tubes)
1 tourniquet
1 tablet
1 unique identification number
1 blue pad to put under the arm of the child

Immobilization

Ask if the mom/guardian of the child would like to help by holding the child. If the parent accepts to hold the child, provide full instructions:

- Stand on opposite side of the table
- Ask the person holding the child to:
    - Stretch an arm across the table and place the child on its back, with its head on top of the outstretch arm
    - Pull the child close, as if you were cradling with the child

- Grasp the child's elbow in the outstretched hand
- Use your other arm to reach across the child and grasp its wrist in a palm up position (reaching across the child anchors the child's shoulder, and thus prevents twisting and rocking movements)
- If the child can sit on its own:
    - Sit the child on your lap, facing the examiner
    - Hold the legs of the child between your legs
    - Extend the child's arm and grasp the child's elbow
    - With your other hand grasp the child's wrist in a palm up position

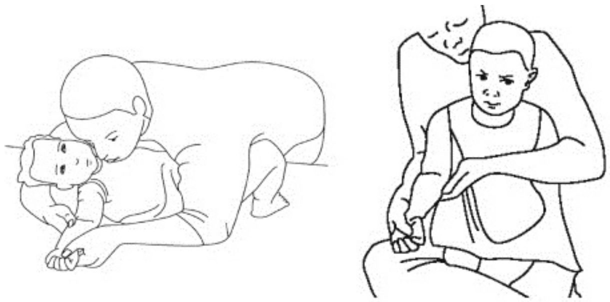
Immobilize the baby or child.

Use the pillow to make the child more comfortable or to hold is elbow. Comfort is very important for the child to feel safe.

Explaining the person who is helping you how to hold the child is very important, make sure the person understands what you need from him/her

Reassure the child, talk to the child with a calm voice, and touch the child gently

<u>Procedure</u>

1. Collect supplies and equipment
2. Wash your hands
3. Look at the arms of the child to try to find a good venipuncture site
4. Wash your hands
5. Immobilize the child
6. Put the tourniquet on the child about 2 fingers widths above the venipuncture site chosen
7. Put on your gloves
8. Attach the end of the tubing to the end of the holder
9. Remove the plastic sleeve from the needle
10. Using the alcohol swab, disinfect the venipuncture site and allow it to dry
11. Draw the skin using your tumb and stick the needle in the vein
12. Push the tube onto the holder until the needle is in it
13. Blood should begin to flow into the tube
14. Fill the tube until it is full, remove the tube
15. Release the tourniquet
16. Place a dry coton ball over the venipuncture site and withdraw the needle
17. Ask the parent/guardian to continue applying mild pressure

18. Dispose of the needle in the sharps container
19. Dispose of all contaminated supplies in the trash
20. Label the tube with a label containing a unique number
21. Put a bandage on the child’s arm and ask the mom to remove it 1h later
22. Gently and completely invert the tube 10 times in order ro mix blood with the tube preservation agents
23. Remove gloves, put them in the trash
24. Wash your hands

<u>Illustrations</u>

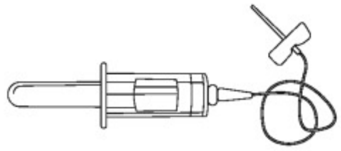

1. Use a winged steel needle, usually 23 or 25 gauge, with an extension tube (butterfly). Keep the tube and needle separate until the needle is in the vein.

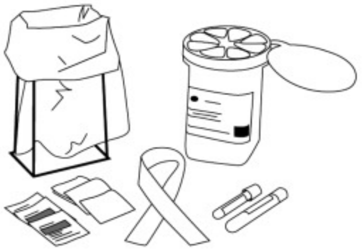

2. Collect supplies and equipment.

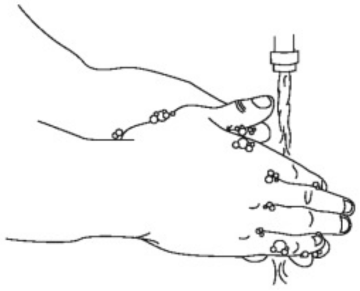

3. Perform hand hygiene (if using soap and water, dry hands with single-use towels).

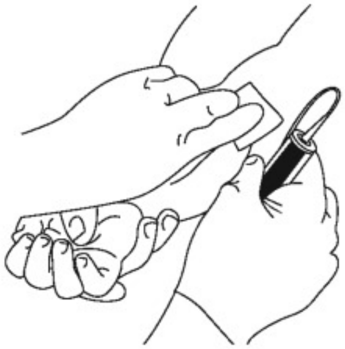

15. Place dry gauze over the venepuncture site and slowly withdraw the needle.

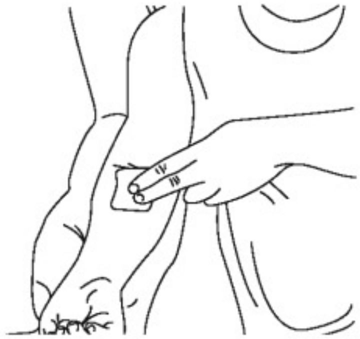

16. Ask the parent to continue applying mild pressure.

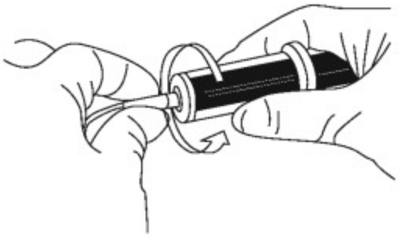

17. Remove the butterfly from the vacuum tube holder.

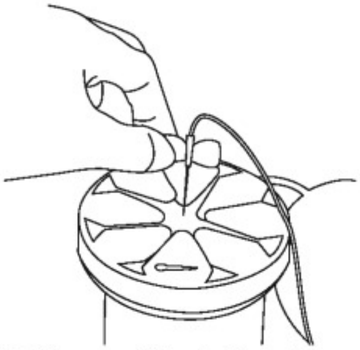

18. Dispose of the butterfly in a sharps container.

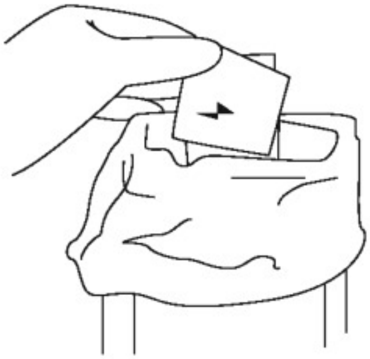

19. Properly dispose of all contaminated supplies.

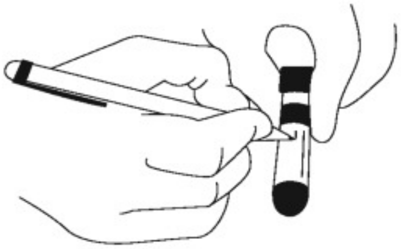

20. Label the tube with the patient identification number and date.

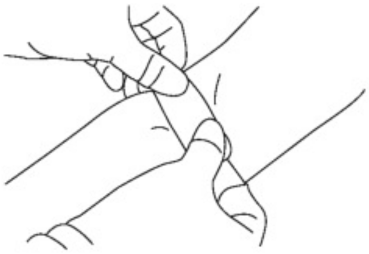

21. Put an adhesive bandage on the patient if necessary.

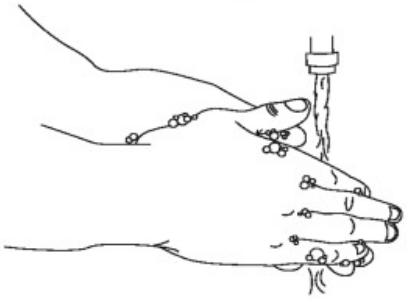

22. Remove gloves, dispose of them appropriately and perform hand hygiene (if using soap and water, dry hands with single-use towels).

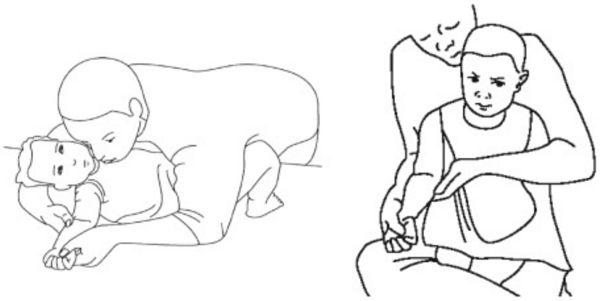

4. Immobilize the baby or child.

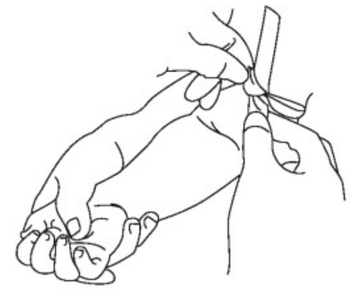

5. Put the tourniquet on the patient about two finger widths above the venepuncture site.

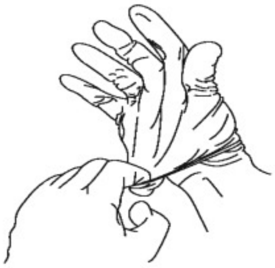

6. Put on well-fitting, non-sterile gloves.

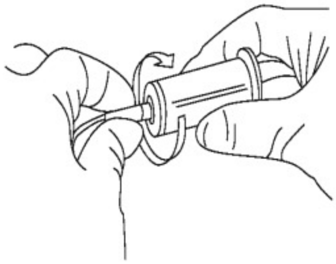

7. Attach the end of the winged infusion set to the end of the vacuum tube and insert the collection tube into the holder until the tube reaches the needle.

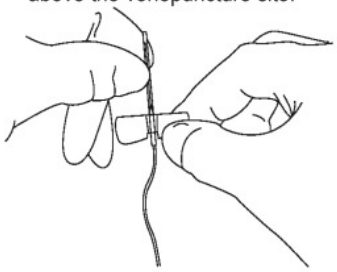

8. Remove the plastic sleeve from the end of the butterfly.

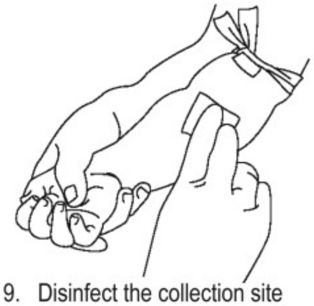

9. Disinfect the collection site and allow to dry.

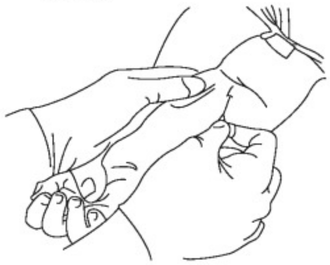

10. Use a thumb to draw the skin tight, about two finger widths below the venepuncture site.

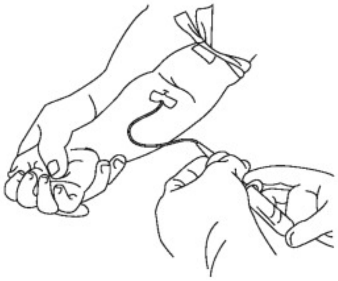

11. Push the vacuum tube completely onto the needle.

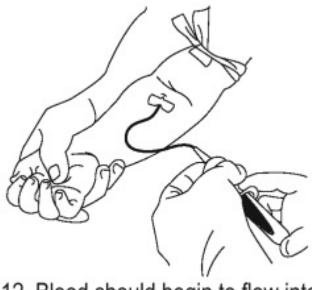

12. Blood should begin to flow into the tube.

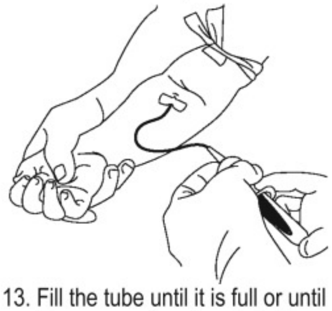

13. Fill the tube until it is full or until the vacuum is exhausted; if filling multiple tubes, carefully remove the full tube and replace with another tube, taking care not to move the needle in the vein.

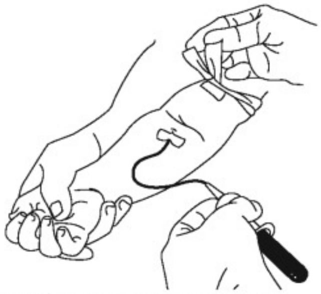

14. After the required amount of blood has been collected, release the tourniquet.

Tips and Warnings

Make sure the right child is in front of you, verify his/her identity with the mom/guardian

Take your time to choose your venipuncture site. Choose the venipuncture site while the child is still in his/her mom's arm, when s/he feels safe.

Keep the child warm, it will help dilate the vein and increase the blood flow: use swaddling blanket, let the mother hold the child/infant, warm the area of puncture with warm cloths.

Ask the person helping you holding the child if they can rhythmically tighten and release the child's wrist to ensure that there is an adequate flow of blood

Let the child suck on something (pacifier, breast) during the venipuncture if needed

Distract the attention of the child away from the puncture

Do not touch the cleaned site, if the cleaned site is contaminated, repeat the disinfection

Do not let the tourniquet in place more than 2 minutes

Do not attempt the venipuncture more than 3 times

10 Chapter 10: Anthropometry

Anthropometry will be collected for all children at baseline, 14 days, and 180 days after the completion of the treatment. We will measure length or height, weight, and MUAC.

10.1 Measuring Length and Height

A lightweight measuring board will be used to measure the participant's height to the nearest 0.1 cm.

Length or Height will be assessed with a ShorrBoard. Depending on a child's age and ability to stand, measure the child's length or height.

Length: If a child is less than 2 years old, or cannot stand alone, measure recumbent length. A child's length is measured lying down (recumbent).

Height: If a child is able to stand, we will measure standing height.

10.2 Procedure

If the child has braids or hair ornaments that will interfere with length/height measurements, remove them if possible. Check that any sandals, shoes, or socks have also been removed.

Whether measuring length or height, the mother/guardian is needed to help with measurements and to soothe and comfort the child. Explain to the mother the reasons for the measurements, and describe the steps in the procedure. Answer any questions she might have. Show her and tell her how she can help you. Explain that it is important to keep the child still and calm to obtain the best measurement.

ShorrBoard Set-Up

1) Remove ShorrBoard from bag.
2) Stand the ShorrBoard upright. You can step on the base of the board to keep it stable.
3) As you face the board, turn the non-removable bolt counterclockwise to release the extension piece. Note: the bolt remains attached to the back of the extension piece – do NOT remove it.
4) Slide the extension piece into the top end of the main board and fasten the clasp on the back of the board. Make sure the clasp is fastened properly.
5) The auto-lock sliding head/footpiece is stored in the base of the main board and can be moved up and down the length of the measuring board. It should stay in place on its own wherever you position it.
6) The measuring board must be placed against a firm surface for standing height (e.g. wall, table, tree, against a vehicle, etc.). Make sure the board is stable. If necessary, place items such as small rocks underneath the height board to stabilize it during the measurement.
7) Clean the equipment with alcohol swabs at the beginning of each day.

Note: when you set up the board each day, examine each of the pieces to check for damage.

10.3 Measure Length

Cover the length board with a chuck for hygiene and for the baby's comfort. Explain to the mother that she will need to place the baby on the length board and then help to hold the baby's head in place while the measurement is taken. Show her where to stand when placing the baby down (i.e. opposite you, on the side of the length board away from the tape). Also show her where to place the baby's head (against the fixed headboard) so that she can move quickly and surely without distressing the baby. When the mother understands your instructions and is ready to assist: Ask her to lay the child on his back with his head against the fixed headboard, compressing the hair.

Quickly position the head so that an imaginary vertical line from the ear canal to the lower border of the eye socket is perpendicular to the board. (The child's eyes should be looking straight up.) Ask the mother to move behind the headboard and hold the head in this position.

Speed is important. Standing on the side of the length board where you can see the measuring tape and move the footboard: Check that the child lies straight along the board and does not change position. Shoulders should touch the board, and the spine should not be arched. Ask the mother to inform you if the child arches the back or moves out of position. Hold down the child's legs with the one hand and move the footboard with the other. Apply gentle pressure to the knees to straighten the legs as far as they can go without causing injury or distress.

Note: it is not possible to straighten the knees of newborns to the same degree as older children. Their knees are fragile and could be easily injured, so apply only minimum pressure.

If a child is extremely agitated and both legs cannot be held in position, measure with one leg in position. While holding the knees, pull the footboard against the child's feet. Upon reading the measurement, the examiner will clearly call out the number to the recorder. Record the child's length in centimeters to the last completed 0.1 cm. (1.0 mm). Keeping the child in place, release the sliding footboard, and prepare to repeat the measurement. Re-position the child for a second and third measurement.

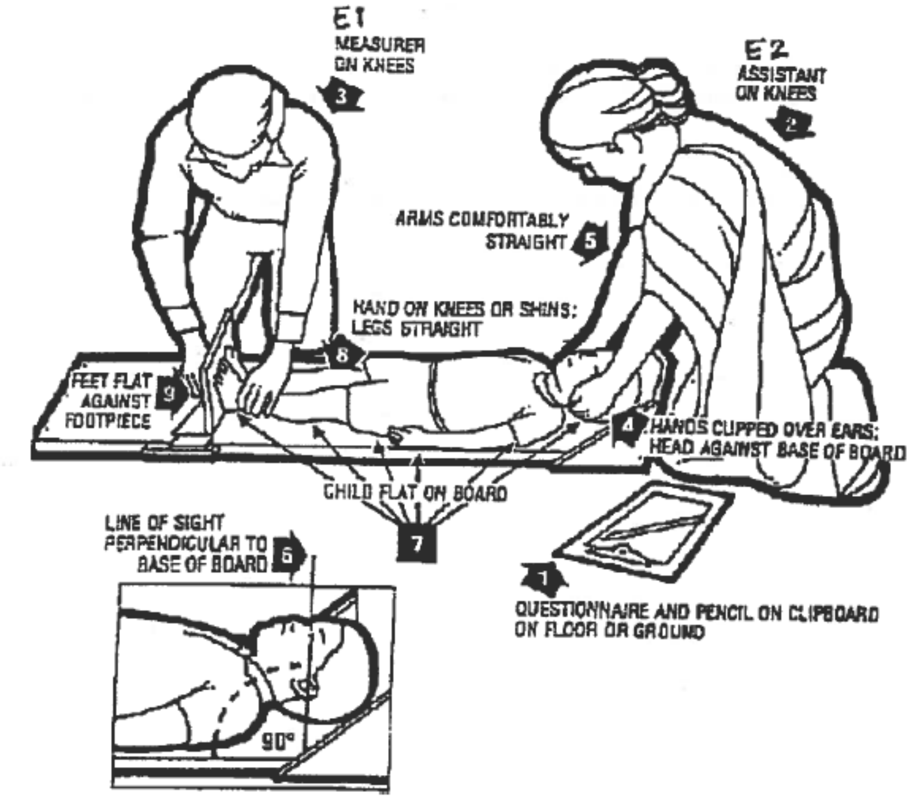

10.4 Measure Standing Height

Ensure that the height board is on level ground. Working with the mother, and kneeling in order to be at the level of the child. Help the child stand on the baseboard with the weight of the child evenly distributed on both feet. The heels of the feet are placed together with both heels touching the base of the vertical board. Place the feet pointed slightly outward at a 60 degree angle. The back of the head, shoulder blades, buttocks, calves, and heels should all touch the vertical board. Arms should hang freely by the sides of the body with the palms facing the thighs.
Note: Standing with all body parts touching the board may be difficult for some children, in which case, help the child to stand on the board with one or more contact points touching the board.
Ask the mother to hold the child's knees and ankles to help keep the legs straight and feet flat, with heels and calves touching the vertical board. Ask her to focus the child's attention, soothe the child as needed, and inform you if the child moves out of position. Position the child's head so that a horizontal line from the ear canal to the lower border of the eye socket runs parallel to the baseboard. Ask the child to inhale deeply and to stand fully erect without altering the position of the heels. If necessary, push gently on the belly to help the child stand to full height.
Still keeping the head in position, use your other hand to pull down the headboard to rest firmly on top of the head and compress the hair. Upon reading the measurement, the examiner will clearly call out the number to the recorder. Record the child's height in centimeters to the last completed 0.1 cm (1.0 mm). Keeping the child in place, release the sliding headboard, and prepare to repeat the measurement. Re-position the child for a second and third measurement.

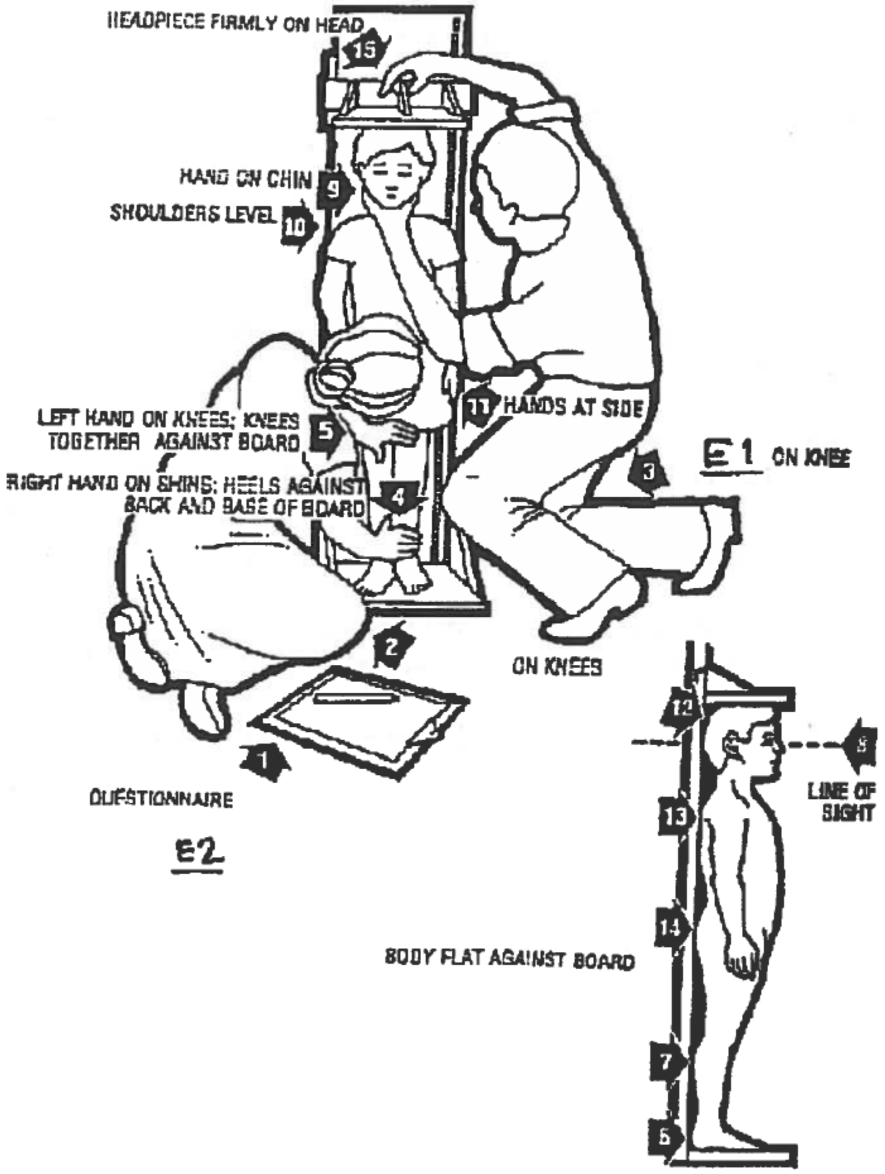

10.4.1 <u>Dismantling the ShorrBoard</u>

1) Stand the board upright: face the board and step on the base with one foot to keep it stable.
2) Slide the head/footpiece into the base of the main board.
3) Release the clasp on the back of the extension piece and remove it. Push the clasp FLAT against the extension piece.
4) To attach the extension piece to the main board, turn the front of the extension piece inward and place it against the front of the main board. Make sure that all sides of the extension piece are straight and in line with the main board.
5) Push on the bolt that is on the back of the extension piece and screw it into the main board.

6) Put the board back inside of the carrying case for storage until your next use.

10.5 Measuring Weight

The SECA 874 scale will be used to weigh infants and children to the nearest 0.01 kg. Infants and young children can also be weighed simultaneously with their parent or guardian by the unique “mother-baby” function (parent or guardian is weighed and then the infant or child is weighed while held by the parent). Explain to the mother that we want to weigh her child to see how he or she is growing. If she has a baby or a child who is unable to stand, she will hold the child on the scale. If the child is 2 years or older/can stand alone, the child will be weighed alone. Children should be wearing only light clothing, no shoes or sandals, no hair ornaments, and no jewelry. Explain that the child needs to remove outer clothing and shoes/sandals in order to obtain an accurate weight. If the baby is wearing a diaper, the diaper should be removed. If any heavy clothes remain on the child, make a note in the Notes section.
Seca 874 Scale

1) Remove scale from bag.
2) Be sure that the scale is placed on a flat, hard, even surface. All 4 legs of the scale should make contact with the ground surface, without wobbling. It may be helpful to place a piece of plywood on the ground underneath the scale.
3) When batt appears in the display, you should change the batteries. Remove the old batteries and insert 6 new batteries.

Turn the power on the scale when you are ready to begin weighing. If the child is unable to stand on the scale, you will use the 2 in 1 weighing function (called tared weighing). The 2 in 1 function enables the weight of babies and small children to be determined while an adult holds them. Identify a suitable area that is flat for horizontal placement of the scale. Press the start key with no load on the scale. The scale is ready for use when it sets to 0.00. If necessary, switch the weight display to KG: hold down the 2 in 1 key for about 3 seconds. Press the start key with no load on the scale. Wait until the display shows 0.00.
Ask the adult to remove his/her shoes and stand in the middle of the scale without the child. S/he should remove any long garments, as these can cover the display and also lead to variable measurements. After the adult’s weight appears on the display, tell him/her to remain standing on the scale. Press the 2 in 1 key to activate the function. The scale stores the weight of the adult and the display returns to zero. When 0.00 and NET appear in the display, hand the child to the adult. The scale will determine the weight of the child. Once the value is stable for about 3 seconds, the weight is measured.
Note: If an adult is very heavy (e.g. more than 100 kg) and the baby’s weight is relatively low (e.g. less than 2.5 kg), the baby’s weight may not register on the scale. In such cases, have a lighter person hold the baby on the scale.

The positioner will clearly call out the child’s weight to the recorder. Record the child’s weight to the nearest 0.01 kg. Repeat the measurement 2 more times. Note that only the baby needs to removed from the scale; the adult should remain on the scale the entire time. To turn off the 2 in 1 function, press the 2 in 1 key. The 2 in 1 function remains on until you press the 2 in 1 key again, or until the scale switches off automatically. If several children are to be weighed consecutively with the same adult holding the babies, it is important that this person’s weight does not change (e.g. due to a piece of clothing being removed/added). If the child is able to stand on the scale, you will weigh the child alone. Talk with the child about the need to stand still. Communicate with the child in a sensitive, non-frightening way. Press

the start key with no load on the scale. The scale is ready for use when it sets to 0.00. Ask the child to stand in the middle of the scale. Once on the scale, the child must stand still. The HOLD function is automatically activated for weights over 1.5 kg/3.3 lbs. The display flashes until a stable weight has been measured. The display is then frozen until the next weighing operation.
Note: If the child jumps on the scale or won't stand still, you will need to use the tared weighing procedure instead.

The positioner will clearly call out the child's weight to the recorder. Record the child's weight to the nearest 0.01 kg. Repeat the measurement 2 more times. Have the child move completely off the scale and then stand again on the scale. If no further weighing operations are performed, the scale switches off automatically after 2-3 minutes.

10.5.1 Scale Calibration

In order to monitor the calibration of the scales over time, each team will weigh a 5 kg test weight at the beginning and end of the day. This measurement will take place at the site of the scale storage so that the weights do not need to be carried to the field. In some cases, these weights will be kept in a project vehicle.

10.6 Measuring Mid-Upper Arm Circumference (MUAC)

The child's MUAC will be measured one time. MUAC measurements will be taken at the midpoint of the left arm between the tip of the shoulder and the tip of the elbow using non-stretch MUAC tapes.

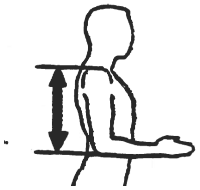

First, find the approximate midpoint of the upper arm. Have the child stand up straight with feet together, and the right arm bent 90 degrees at the elbow, palm facing up. The examiner is positioned behind the child. If it is helpful, mark the midpoint with a permanent marker. To measure mid-upper arm circumference, have the child stand up straight with the arms relaxed at the sides. The examiner will stand facing the child's right side. The measuring tape is placed around the upper arm at the marked point. Wrap the tape around the arm, pulling it to lie flat against the surface of the skin. Be careful not to pull the tape too tightly (to compress the skin).

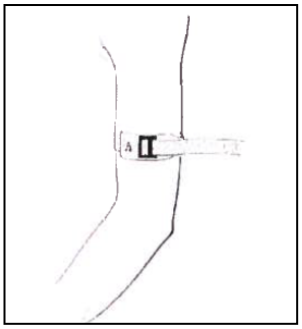

The line for reading measurements is clearly labeled on the MUAC tape (READ (cm)). Read the number aligned with the measurement line on the tape Upon measuring, the examiner will clearly call out the number to the recorder. Record to the nearest 0.1 cm (0.1 cm = 1 mm). Keeping the child in place, release the MUAC strip.

If the MUAC is <11.5cm refer the child to the nearest health center.

11 Chapter 11: Vital Status

Vital status will be recorded at all follow-up visits, including whether the child is alive, has died, or has moved. Date of death and location of move will be recorded.

12 Chapter 12: Data Collection, Management, and Security

12.1 Data Collection

Data will be collected on enrolled children at baseline (time of enrollment), and follow-up at days 14 and 180. Written informed consent will be collected on paper and the study nurse will administer the treatment letter based on the tablet app. All data will be collected electronically on mobile devices using a mobile application from Survey Solutions.

12.2 Data Management and Security

Electronic data will be uploaded daily to a secure, password-protected, cloud-based server. All devices used for data collection will be password-protected, as will the mobile application itself. Paper forms will be stored in locked cabinets accessible only by specific study team members at each enrollment site.
No identifiers will be collected electronically. Study data will only be accessible by study team members and investigators in order to protect confidentiality.

12.3 Data Quality and Monitoring

All study team members collecting data will undergo an initial training to learn how to use the mobile devices as well as best practices for data collection. Data collection will be monitored on a weekly basis by the study team. Concerns over data quality and completeness will be relayed to the local study team by email, and refresher trainings and/or additional supervision of data collection by local investigators will be planned as needed. The study team will send quarterly progress reports to the DSMC, including aggregate data on enrollment and follow-up status and adverse events.

13 Chapter 13: Protection of Human Subjects

Before the study begins, each site will obtain formal ethical approval from their respective national ethics committee. In addition, local staff will approach community leaders to describe the study and answer any questions. Study staff will proceed only if local leadership consents to participate. We will obtain written informed consent for each child's participation from their parent or guardian. If, at any time, a parent or guardian elects to withdraw a family member from the study, they will be free to do so. Children with wasting or stunting will be referred for appropriate treatment by trained study personnel to the nearest health center.

13.1 Institutional Review Board Approval

University of California, San Francisco (UCSF) Committee on Human Research

UCSF's Committee on Human Research will annually review the study protocol for ethical approval.

Comité d'Ethique du Burkina Faso
The study protocol will be reviewed and granted ethical approval by the Comité d'Ethique du Burkina Faso before any study activities begin.

13.2 Informed Consent

Study personnel fluent in relevant local languages will approach parents or guardians of eligible children. In a private setting, the study team member will explain the objectives, risks, and benefits of the study as well as detailed information about what study participation entails for the child and the parent or guardian. The study team member will clarify that participation in the study is voluntary, that participation may be stopped at any time, and that all collected data will be kept confidential and securely stored. The study team member will ensure comprehension by inviting the parent or guardian to ask questions and will provide time for the parent or guardian to consider participation. When providing consent, both the study team member and the parent or guardian will sign two copies the consent document. One copy will be given to the parent or guardian and the other will be kept for study reference.

13.3 Risks and Benefits of Study Procedures

13.4 11.3.1 Procedures

Nasopharyngeal swabs: There are minimal risks to the participant who receives nasopharyngeal swabbing. Participants may experience some temporary discomfort, but the swabbing involves minimal risk. Any adverse effects, such as nose-bleeds, will be treated immediately by the examiners. Other health care will be provided at no cost to the study participant if necessary to address a study-related adverse health event.
Stool samples: Fresh frozen stool collection is a simple procedure with very minimal risk.
Stool sample collection is a non-invasive procedure with no associated risks.
Anthropometric Measurements: There are minimal risks associated with the measuring board, scale, or MUAC tapes aside from anxiety during the measurements. Examiners will do their best to ensure that the parent/guardian of the child understands the process of assessing anthropometric measurements. The examiners will attempt to minimize discomfort for all study participants before, during, and after the measurements are taken. Children with wasting or stunting will be referred for appropriate treatment at the nearest health center.
Venipuncture: The standard risks of drawing blood from a vein include discomfort at the site of the puncture, bruising, swelling around the site, and rarely infection. Trained staff will administer the venipuncture and attempt to mitigate any associated risks.
Rectal Swabs: There are minimal risks associated with rectal swab collection. Some discomfort may occur.
Fingerstick (Blood smears): A lancing device will be used to obtain a small sample of blood. There are minimal risks associated with this procedure.
Temperature: Discomfort may be associated with taking the child's core body temperature.

14 Chapter 14: Data Safety and Monitoring Committee

14.1 Data and Safety Monitoring Committee Charter

This Charter is for the Data Safety and Monitoring Committee (DSMC) for the longitudinal MORDOR II Burkina Faso component.

The Charter will define the primary responsibilities of the DSMC, its relationship with other trial components, its membership, and the purpose and timing of its meetings. The Charter will also provide the procedures for ensuring confidentiality and communication, statistical monitoring guidelines to be implemented by the DSMC, and an outline of the content of the Open and Closed Reports that will be provided to the DSMC.

14.2 Primary Responsibilities of the DSMC

The DSMC will be responsible for safeguarding the interests of trial participants, assessing the safety and efficacy of the interventions during the trial, and monitoring the overall conduct of the trial. The DSMC will provide recommendations about stopping or continuing the trial. To contribute to the integrity of the trial, the DSMC may also formulate recommendations relating to the selection/recruitment/retention of participants, to protocol-specified regimens, and the procedures for data management and quality control.

The DSMC will be advisory to the trial leadership group, hereafter referred to as the Steering Committee (SC). The SC will be responsible for promptly reviewing the DSMC recommendations and determining, whether to continue or terminate the trial, and to determine whether amendments to the protocol are required. If needed, the DSMC may seek the advice of a content expert outside of the committee.

14.3 DSMC Membership

The DSMC is an independent multidisciplinary group consisting of epidemiologists, biostatisticians, bioethicists, and clinicians that collectively has experience in pediatrics, the management of infectious diseases, and in the conduct and monitoring of randomized clinical trials including subsaharan Africa.

14.4 Conflicts of Interest

The DSMC membership has been restricted to individuals free of apparent conflicts of interest. The source of these conflicts may be financial, scientific, or regulatory. Thus, neither study investigators nor individuals employed by the sponsor, nor individuals who might have regulatory responsibilities for the trial products, are members of the DSMC.

The DSMC members will disclose to fellow members any consulting agreements or financial interests they have with the sponsor of the trial, with the contract research organizations (CRO) , or with other sponsors having products that are being evaluated or that are competitive with those in the trial. The DSMC will be responsible for deciding whether these consulting agreements or financial interests materially impact their objectivity.

The DSMC members will be responsible for advising fellow members of any changes in any of the membership requirements that occur during the course of the trial. It may be appropriate for DSMC members who develop significant conflicts of interest resign from the DSMC.

DSMC membership is to be for the full duration of the trial. If any members leave the DSMC, the SC, in consultation with the DSMC, will promptly appoint a replacement.

14.5 Timing and Purpose of the DSMC Meetings

Organizational Meeting
The initial meeting of the DSMC will be an Organizational Meeting. This is during the final stages of protocol development and the purpose is to provide advisory review of scientific and ethical issues relating to study design to discuss the standard operating procedures and to discuss the format and content of the Open and Closed Reports that will be used to present trial results.

The Organizational Meeting will be attended by all DSMC members, lead trial investigators, and the trial biostatistician. The DSMC will be given the drafts of the trial protocol, the Statistical Analysis Plan, the DSMC Charter, and the current version of the case report forms. At subsequent meetings, committee members will receive Open and Closed Data Reports.

14.6 Procedures to Ensure Confidentiality and Proper Communication

To enhance the integrity and credibility of the trial, procedures will be implemented to ensure the DSMC has access to all emerging information from the trial regarding comparative results of efficacy and safety, aggregated by treatment arm.

Closed Sessions
Sessions involving only DSMC members and, where appropriate, those unmasked trial investigators (on the Data Coordinating Committee) who generate the Closed Reports (called Closed Sessions) will be held to allow discussion of confidential data from the trial, including information about the relative efficacy and safety of interventions.

At a final Closed Session, the DSMC will develop a consensus on its list of recommendations, including that relating to whether the trial should continue.

Open Session
In order for the DSMC to have access to information provided, by study investigators, or members of regulatory authorities, a joint session between these individuals and DSMC members will be held between the Closed Sessions.

Open and Closed Reports
For each DSMC meeting, Open and Closed Reports will be provided. Open Reports, will include data on recruitment and baseline characteristics, pooled data on eligibility violations, and completeness of follow-up and compliance. The study statistician (TCP) will prepare these Open Reports.

Closed reports, available only to those attending the Closed Sessions of the meeting, will include analyses of primary and secondary efficacy endpoints, including subgroup and adjusted analyses, AEs and symptom severity, , and Open Report analyses that are displayed by intervention group. These Closed Reports will be prepared by the study biostatistician.

The Open and Closed Reports should provide information that is accurate, with follow-up that is complete to within two months of the date of the DSMC meeting. The Reports should be provided to DSMC members approximately three days prior to the date of the meeting.

Minutes of the DSMC Meeting
The research team will prepare minutes for the open portion of the meeting, including the DSMC's recommendations.

Recommendations to the Steering Committee (SC)
At each meeting of the DSMC during the trial, the committee will make a recommendation to the Steering Committee to continue or terminate. This recommendation will be based primarily on safety and efficacy considerations and will be guided by statistical monitoring guidelines defined in this Charter.

Recommendations to amend the protocol or conduct of the study made by the DSMC will be considered and accepted or rejected by the SC. The SC will be responsible for deciding whether to continue or to stop the trial based on the DSMC recommendations.

The DSMC will be notified of all changes to the protocol or to study conduct. The DSMC concurrence will be sought on all substantive recommendations or changes to the protocol or study conduct prior to implementation.

The SC may communicate information in the Open Report to the sponsor and may inform them of the DSMC recommended alterations to study conduct or early trial termination in instances in which the SC has reached a final decision agreeing with the recommendation. The SC will maintain confidentiality of all information it receives other than that contained in the Open Reports until after the trial is completed or until a decision for early termination has been made.

14.7 Statistical Monitoring Guidelines
The SC will propose statistical rules for a futility stopping rule (requested by the sponsor) and an efficacy stopping rule at the first DSMC meeting. A decision will be made whether the efficacy stopping rule is appropriate for the study.

15 Chapter 15: Study Team Roles and Responsibilities

- Investigators (UCSF): UCSF will be responsible for study design and implementation, data management and monitoring, data analysis, and dissemination of results. The UCSF investigators will design and implement trainings for study procedures and maintain frequent communication with local Nouna study staff
- Investigators (Nouna): The Nouna investigators will contribute to the study design, implementation, data management and monitoring, data analysis, and dissemination of the results. The Nouna team will oversee all local study activities, including training and regular supervision and monitoring of all local study staff.
- Medical Monitor: The Medical Monitor will provide clinical oversight for the enrolled children. Study team members will report serious adverse events to the Medical Monitor within 24 hours of occurrence. The Medical Monitor will determine if the event is likely to be related to azithromycin and provide clinical guidance for the adverse event as needed.
- Data and Safety Monitoring Committee: The Data and Safety Monitoring Committee will provide independent oversight of data quality and patient safety during the course of the study.
- Study Team Members (Nouna): Study team members in Nouna will be responsible for implementing all study procedures including recruitment, consent, enrollment, randomization, drug administration, and collection of all study data.
    - Hospital Staff: Hospital staff will be trained to determine the randomization allocation of each enrolled child by utilizing the study tablets and administer azithromycin if indicated
    - Outcome Assessor: One outcome assessor will be trained to manage consent, enrollment, and collection of all study data, including outcome assessments

16 References

1 Keenan JD, Bailey RL, West SK, et al. Mass azithromycin distribution for reducing childhood mortality in sub-Saharan Africa. N Engl J Med 2018; In press.

2 World Health Organization. (2018, May 29). Presumptive use of azithromycin. Retrieved from http://www.who.int/maternal_child_adolescent/guidelines/development/provision-of-azithromycin-to-infants/en/

3 Wang H, Bhutta ZA, Coates MM, et al. Global, regional, national, and selected subnational levels of stillbirths, neonatal, infant, and under-5 mortality, 1980–2015: a systematic analysis for the Global Burden of Disease Study 2015. The Lancet 2016; 388: 1725–74.

4 Beiersmann C, Bountogo M, Tiendrébeogo J, et al. Malnutrition in young children of rural Burkina Faso: comparison of survey data from 1999 with 2009. Tropical Medicine & International Health 2012; 17: 715–21.

5 Porco TC, Gebre T, Ayele B, et al. Effect of Mass Distribution of Azithromycin for Trachoma Control on Overall Mortality in Ethiopian Children: A Randomized Trial. JAMA 2009; 302: 962–8.

6 Amza A, Yu SN, Kadri B, Nassirou B, Stoller NE, Zhou Z, et al. (2014) Does Mass Azithromycin Distribution Impact Child Growth and Nutrition in Niger? A Cluster-Randomized Trial. PLoS Negl Trop Dis 8(9): e3128. https://doi.org/10.1371/journal.pntd.0003128

7 Angelakis E, Merhej V, Raoult D, 2013. Related actions of probiotics and antibiotics on gut microbiota and weight modification. Lancet Infect Dis 13: 889–899.</

8 Ranells JD, Carver JD, Kirby RS. Infantile Hypertrophic Pyloric Stenosis: Epidemiology, Genetics, and Clinical Update. Advances in Pediatrics 2011; 58: 195–206.

9 Everett KV, Ataliotis P, Chioza BA, Shaw-Smith C, Chung EMK. A novel missense mutation in the transcription factor FOXF1 cosegregating with infantile hypertrophic pyloric stenosis in the extended pedigree linked to IHPS5 on chromosome 16q24. Pediatr Res 2016; 81: 632–8.

10 Peeters B, Benninga MA, Hennekam RCM. Infantile hypertrophic pyloric stenosis —genetics and syndromes. Nature Publishing Group 2012; 9: 646–60.

11 Svenningsson A, Svensson T, Akre O, Nordenskjöld A. Maternal and pregnancy characteristics and risk of infantile hypertrophic pyloric stenosis. Journal of Pediatric Surgery 2014; 49: 1226–31.

12 Zhu J, Zhu T, Lin Z, Qu Y, Mu D. Perinatal risk factors for infantile hypertrophic pyloric stenosis: A meta-analysis. Journal of Pediatric Surgery 2017; 52: 1389–97.

13 Tadesse A, Gadisa A. Infantile hypertrophic pyloric stenosis: A retrospective study from a tertiary hospital in Ethiopia. East Cent Afr J Surg 2014; 19: 120–4.

14 Wayne C, Hung J-HC, Chan E, Sedgwick I, Bass J, Nasr A. Formula-feeding and hypertrophic pyloric stenosis: is there an association? A case–control study. Journal of Pediatric Surgery 2016; 51: 779–82.

15 Krogh C, Biggar RJ, Fischer TK, Lindholm M, Wohlfahrt J, Melbye M. Bottle-feeding and the Risk of Pyloric Stenosis. Pediatrics 2012; 130: e943–9.

16 Sommerfield T, Chalmers J, Youngson G, Heeley C, Fleming M, Thomson G. The changing epidemiology of infantile hypertrophic pyloric stenosis in Scotland. Archives of Disease in Childhood 2008; 93: 1007–11.

17 Osifo DO, Evbuomwan I. Does Exclusive Breastfeeding Confer Protection Against Infantile Hypertrophic Pyloric Stenosis? A 30-year Experience in Benin City, Nigeria. Journal of Tropical Pediatrics 2008; 55: 132–4.
18 Murchison L, Coppi P, Eaton S. Post-natal erythromycin exposure and risk of infantile hypertrophic pyloric stenosis: a systematic review and meta-analysis. Pediatric Surgery International 2016; 32: 1147–52.
19 Smith C, Egunsola O, Choonara I, Kotecha S, Jacqz-Aigrain E, Sammons H. Use and safety of azithromycin in neonates: a systematic review. BMJ Open 2015; 5: e008194–8.
20 Ballard HO, Anstead MI, Shook LA. Azithromycin in the extremely low birth weight infant for the prevention of Bronchopulmonary Dysplasia: a pilot study. Respir Res 2007; 8: 1793–9.
21 Ballard HO, Shook LA, Bernard P, et al. Use of azithromycin for the prevention of bronchopulmonary dysplasia in preterm infants: a randomized, double-blind, placebo controlled trial. Pediatr Pulmonol 2010; 46: 111–8.
22 Gharehbaghi MM, Peirovifar A, Ghojazadeh M, Mahallei M. Efficacy of azithromycin for prevention of bronchopulmonary dysplasia (BPD). Turk J Med Sci 2012; 42: 1070–5.
23 Friedman DS, Robinette Curtis C, Schauer SL, et al. Surveillance for Transmission and antibiotic Adverse Events Among Neonates and Adults Exposed to a Healthcare Worker With Pertussis. Infect Control Hosp Epidemiol 2015; 25: 967–73.
24 Eberly MD, Eide MB, Thompson JL, Nylund CM. Azithromycin in Early Infancy and Pyloric Stenosis. Pediatrics 2015; 135: 483–8.
25 Said M, Shaul DB, Fujimoto M, Radner G, Sydorak RM, Applebaum H. Ultrasound Measurements in Hypertrophic Pyloric Stenosis: Don't Let the Numbers Fool You. The Permanente Journal 2012; 16: 25–7.

APPENDIX

Appendix 1: Study Forms
Appendix 2: SAP

SURVEY IDENTIFICATION INFORMATION QUESTIONNAIRE DESCRIPTION

Basic information

Title	1-GAMIN - Eligibilité

Survey data information

Study type	Statistical Info and Monitoring Prog.
Kind of data	Clinical data [cli]
Mode of Data Collection	Face-to-Face

Survey information

Country	Burkina Faso
Year	2019
Languages	English, French
Unit of analysis	Individual-level
Coverage	Neonates and children aged 8 days to 59 months
Primary Investigator	Catherine E Oldenburg
Funding	The Bill & Melinda Gates Foundation

Additional info

Notes	Eligibility form for GAMIN trial

ELIGIBILITY

Tap to record the time	DATE: CURRENT TIME — `startTime` ....
VARIABLE `startTime.Value.Year`	LONG — `startYear`
VARIABLE `"blue"`	STRING — `colorName`
What is the child's first name?	TEXT — `childFirstname` ....
What is %childFirstname%'s last name?	TEXT — `childLastname` ....
Is %childFirstname% under 1 month old? I Ask for birth document! E `false`	SINGLE-SELECT — `under1mo` 01 ○ Yes 02 ○ No
What is %childFirstname%'s birthdate? E `under1mo==1`	SINGLE-SELECT — `childDoBmode` 01 ○ Enter date 02 ○ Don't know
%childFirstname%'s birthdate E `childDoBmode==1` V1 `(ageInDays>0 && ageInDays<=35)` M1 Please check this!	DATE — `childDoB` ....
Birthdate - Year V1 `self.InRange(1900, startYear) || self==9999` M1 Must be between 1900 and %startYear%	NUMERIC: INTEGER — `yearN` _ _ _ _ _ _ _ _ SPECIAL VALUES 9999 Don't know
Birthdate - Month E `yearN.InRange(1900,startYear)`	SINGLE-SELECT — `monthN` 01 ○ January 02 ○ February 03 ○ March 04 ○ April 05 ○ May 06 ○ June 07 ○ July 08 ○ August 09 ○ September 10 ○ October 11 ○ November 12 ○ December 99 ○ Don't know

Birthdate - Day

SINGLE-SELECT — dayN

F (@optioncode==30 && monthN!=2) || (@optioncode==31 && monthN.InList(1,3,5,7,8,10,12)) || @optioncode.InRange(1,29) || @optioncode==99

E `monthN.InRange(1,12)`

- 01 ○ 1
- 02 ○ 2
- 03 ○ 3
- 04 ○ 4
- 05 ○ 5
- 06 ○ 6
- 07 ○ 7
- 08 ○ 8
- 09 ○ 9
- 10 ○ 10
- 11 ○ 11
- 12 ○ 12
- 13 ○ 13
- 14 ○ 14
- 15 ○ 15
- 16 ○ 16

And 16 other symbols [1]

Reported age in years or months?

SINGLE-SELECT — reportedAgeMetric

E `yearN==9999`

- 01 ○ Years
- 02 ○ Months

Age de %childFirstname%:

NUMERIC: INTEGER — reportedAge

E `yearN==9999`

_ _

VARIABLE

LONG — ageInMonths

```
IsAnswered(reportedAge) && reportedAgeMetric==1 ? (long)
reportedAge*12 : IsAnswered(reportedAge) && reportedAgeM
etric==2 ? (long) reportedAge : CenturyMonthCode(startTi
me.Value.Month, startTime.Value
```

And 205 other symbols [1]

VARIABLE

LONG — ageInDays

```
/* if day of month unknown use 15th -> max.error 2 weeks
*/ (IsAnswered(reportedAge) && reportedAgeMetric==1) ? (
(long)reportedAge * 365) : (IsAnswered(reportedAge) && r
eportedAgeMetric==2) ? ((
```

And 432 other symbols [2]

VARIABLE

DOUBLE — ageInYears

`ageInDays/365`

STATIC TEXT

E `ageInDays>365`

%childFirstname% is %ageInYears% years old

STATIC TEXT

E `ageInMonths>=1 && ageInMonths < 12`

%childFirstname% is %ageInMonths% months old

STATIC TEXT

E `ageInDays<31 && ageInDays>0`

%childFirstname% is %ageInDays% days old

STATIC TEXT

E
```
((IsAnswered(childDoB) || (yearN.InRange(1900,startYear)) || /* && IsAnswered(monthN) && IsAnswered(dayN)*/ IsAnswer
ed(reportedAge)) && (ageInDays<8 || ageInDays>=1825 || ageInMonths>=60))
```

%childFirstname% is not eligible for the study
because %childFirstname% is not between 8 days and 59 months old.

Question	Response
How did you verify date of birth?	SINGLE-SELECT docDoB<br>01 ○ Carnet de sante<br>02 ○ Birth certificate<br>03 ○ CNIB/Passport<br>04 ○ Someone told me<br>05 ○ Other document
Who told you?<br>E docDoB==4	TEXT docDoB4<br>..
What other document?<br>E docDoB==5	TEXT docDoB5<br>..
What is %childFirstname%'s sex?	SINGLE-SELECT childSex<br>01 ○ Male<br>02 ○ Female
What is %childFirstname%'s mother's/guardian's name?<br>I Enter first and last name	TEXT motherFullname<br>..
Does mother/guardian have nickname?	SINGLE-SELECT motherNicknameQ<br>01 ○ Yes<br>02 ○ No
Enter nickname<br>E motherNicknameQ==1	TEXT motherNickname<br>..
What is %childFirstname%'s father's name?<br>I Enter first and last name	TEXT fatherFullname<br>..
Does father have nickname?	SINGLE-SELECT fatherNicknameQ<br>01 ○ Yes<br>02 ○ No
Enter nickname<br>E fatherNicknameQ==1	TEXT fatherNickname<br>..
Is household's phone number available?	SINGLE-SELECT hhPhoneyn<br>01 ○ Yes<br>02 ○ No
Household's phone number<br>E hhPhoneyn==1	TEXT hhPhone<br>..
Is neighbor's phone available<br>E hhPhoneyn==2	SINGLE-SELECT nbPhoneyn<br>01 ○ Yes<br>02 ○ No
Neighbor's phone number<br>E nbPhoneyn==1	TEXT nbPhone<br>..

Please describe how to find your house	TEXT houseLocation<br>..

ELIGIBILITY
CHILD INFORMATION

Primary residence within Nouna?	SINGLE-SELECT primaryResidence<br>01 ○ Yes<br>02 ○ No
Available for the next 6 months?	SINGLE-SELECT available6months<br>01 ○ Yes<br>02 ○ No
Known allergy to macrolides / azalides?	SINGLE-SELECT allergy<br>01 ○ Yes<br>02 ○ No
Has your child been enrolled in NAITRE or supplementary rectal swab study?	SINGLE-SELECT otherStudy<br>01 ○ Yes<br>02 ○ No
Ability to feed orally?	SINGLE-SELECT feedOrally<br>01 ○ Yes<br>02 ○ No

STATIC TEXT

E `$notEligible`

Child is not eligible

Do you consent to participate in study?<br>E `$Eligible && IsAnswered(feedOrally)`<br>W1 `self == 2`<br>M1 Give copy of consent to mother!	SINGLE-SELECT consent<br>01 ○ Yes<br>02 ○ No
Please give the child a study ID, give the mom the study card, and scan the code<br>E `consent == 1`	SINGLE-SELECT childIDmode<br>01 ○ Scan the code<br>02 ○ Code can't be scanned
Scan %childFirstname%'s ID<br>E `childIDmode==1`<br>V1 `self.ToUpper().ToCharArray()[0]=='L'`<br>M1 Invalid ID !<br>V2 `TL!=null`<br>M2 Invalid ID !	BARCODE childIDbarcode
Enter %childFirstname%'s ID<br>E `childIDmode==2`<br>V1 `self.ToUpper().ToCharArray()[0]=='L'`<br>M1 Invalid ID !<br>V2 `TL!=null`<br>M2 Invalid ID!	TEXT childIDmanual<br>..

ELIGIBILITY
VERIFICATION

E `IsAnswered(childIDmanual) && TL!=null`

Enter the letter in the code

TEXT childIDverification

V1 `self.ToUpper().Substring(0,1) == childIDmanual.ToUpper().Substring(0,1)`

M1 Not the same code ! - Please verify !

Enter the numbers in the code

TEXT childIDverification1

V1 `self.ToUpper().Substring(0,6) == childIDmanual.ToUpper().Substring(1,6)`

M1 Not the same code ! - Please verify !

VARIABLE — STRING childID

```
IsAnswered(childIDbarcode) ? childIDbarcode : childIDmanual
```

VARIABLE — BOOLEAN IDok

```
IsAnswered(childIDmanual) ? childIDverification1.ToUpper().Substring(0,6) == childIDmanual.ToUpper().Substring(1,6) : true /* put existing ID check here */
```

VARIABLE — LONG s

```
(childID.ToCharArray()[1] - 48)*10 + (childID.ToCharArray()[2] - 48)
```

VARIABLE — LONG f

```
(childID.ToCharArray()[4] - 48)*10 + (childID.ToCharArray()[5] - 48)
```

VARIABLE — LONG l

```
new int[]{0,1,2,3,4,5,999,6,999,7,8,999,9,10,999,11,999,12,999,999,13,999,14,999,15,999}[(childID.ToUpper().ToCharArray()[3] - 65)]
```

VARIABLE — LONG rc

```
(f<=30)? /* B01A01-B99Y30 */ l*10+(f-1)*160+(s-1)/10 +1
: /* B01A31-B97F42 */ l*10+(f-30-1)*160+(s-1)/10 +1
```

VARIABLE — LONG c

```
(s-1)%10 +1
```

VARIABLE — DOUBLE tlix

```
0 /* (f<=30) ? new int[]{ (int)lut[(int)rc].d1, (int)lut[(int)rc].d2, (int)lut[(int)rc].d3, (int)lut[(int)rc].d4, (int)lut[(int)rc].d5, (int)lut[(int)rc].d6, (int)lut[(int
```

And 400 other symbols [3]

VARIABLE — LONG idcode

```
(childID.ToCharArray()[1] - 48)*100000 + (childID.ToCharArray()[2] - 48)*10000 + (childID.ToCharArray()[3] - 48)*1000 + (childID.ToCharArray()[4] - 48)*100 + (childID.ToCharArray()[5] - 48)*10 + (chil
```

And 141 other symbols [4]

VARIABLE — STRING TL

```
lut[(int)idcode].letter == 0 ? "VV" : lut[(int)idcode].letter == 1 ? "II" : null
```

STATIC TEXT

E `TL == null && (IsAnswered(childIDbarcode) || IsAnswered(childIDmanual))`

Invalid ID! Please verify!

Take picture of the ID card

PICTURE photoID

E `consent == 1`

STATIC TEXT

SURVEY IDENTIFICATION INFORMATION QUESTIONNAIRE DESCRIPTION

Basic information

Title 2-GAMIN - Visite de Base

BASELINE INFORMATION

Tap to record the date	DATE: CURRENT TIME `startTime`
VARIABLE<br>`startTime.Value.Year`	LONG `startYear`
VARIABLE<br>`"blue"`	STRING `colorName`
What is the child's first name?	TEXT `childFirstname`
What is %childFirstname%'s last name?	TEXT `childLastname`
Birthdate - Year<br>I Ask for birth document!<br>V1 `self.InRange(1900, startYear) || self==9999`<br>M1 Must be between 1900 and %startYear%	NUMERIC: INTEGER `yearN`<br>SPECIAL VALUES<br>9999 Don't know
Birthdate - Month<br>E `yearN.InRange(1900,startYear)`	SINGLE-SELECT `monthN`<br>01 ○ January<br>02 ○ February<br>03 ○ March<br>04 ○ April<br>05 ○ May<br>06 ○ June<br>07 ○ July<br>08 ○ August<br>09 ○ September<br>10 ○ October<br>11 ○ November<br>12 ○ December<br>99 ○ Don't know

**Birthdate - Day**<br><br>F (@optioncode==30 && monthN!=2) | | (@optioncode==31 && monthN.InList(1,3,5,7,8,10,12)) | | @optioncode.InRange(1,29) | | @optioncode ==99<br>E `monthN.InRange(1,12)`	SINGLE-SELECT dayN<br>01 ○ 1<br>02 ○ 2<br>03 ○ 3<br>04 ○ 4<br>05 ○ 5<br>06 ○ 6<br>07 ○ 7<br>08 ○ 8<br>09 ○ 9<br>10 ○ 10<br>11 ○ 11<br>12 ○ 12<br>13 ○ 13<br>14 ○ 14<br>15 ○ 15<br>16 ○ 16<br><br>And 16 other symbols [1]
**Reported age in years or months?**<br><br>E `yearN==9999`	SINGLE-SELECT reportedAgeMetric<br>01 ○ Years<br>02 ○ Months
**Age de %childFirstname%:**<br><br>E `yearN==9999`<br>V1 `reportedAgeMetric==1 ? self<5 : true`<br>M1 Please check this!<br>V2 `reportedAgeMetric==2 ? self<60 : true`<br>M2 Please check this!	NUMERIC: INTEGER reportedAge<br><br>_ _
VARIABLE<br>`/* if day of month unknown use 15th -> max.error 2 weeks */ (IsAnswered(reportedAge) && reportedAgeMetric==1) ? ((long)reportedAge * 365) : (IsAnswered(reportedAge) && reportedAgeMetric==2) ? ((`<br><br>And 433 other symbols [1]	LONG ageInDays
VARIABLE<br>`IsAnswered(reportedAge) && reportedAgeMetric==1 ? (long) reportedAge*12 : IsAnswered(reportedAge) && reportedAgeMetric==2 ? (long) reportedAge : CenturyMonthCode(startTime.Value.Month, startTime.Value`<br><br>And 205 other symbols [2]	LONG ageInMonths
VARIABLE<br>`ageInDays/365`	DOUBLE ageInYears

STATIC TEXT

E `ageInDays<30 && ageInDays>0`

%childFirstname% is %ageInDays% days old

STATIC TEXT

E `ageInMonths>=1 && ageInMonths < 12`

%childFirstname% is %ageInMonths% months old

STATIC TEXT

E `ageInDays>365`

%childFirstname% is %ageInYears% years old

%childFirstname%'s ID number	SINGLE-SELECT childIDmode<br>01 ○ Scan the code<br>02 ○ Code can't be scanned
Scan %childFirstname%'s ID<br>E `childIDmode==1`<br>V1 `self.ToUpper().ToCharArray()[0]=='L'`<br>M1 Invalid ID !<br>V2 `TL!=null`<br>M2 Invalid ID !	BARCODE childIDbarcode
Enter %childFirstname%'s ID<br>E `childIDmode==2`<br>V1 `self.ToUpper().ToCharArray()[0]=='L'`<br>M1 Invalid ID !<br>V2 `TL!=null`<br>M2 Invalid ID!	TEXT childIDmanual<br>..

BASELINE INFORMATION

VERIFICATION

E `IsAnswered(childIDmanual) && TL!=null`

Enter the letter in the code<br>V1 `self.ToUpper().Substring(0,1) == childIDmanual.ToUpper().Substring(0,1)`<br>M1 Not the same code ! - Please verify !	TEXT childIDverification<br>..
Enter the numbers in the code<br>V1 `self.ToUpper().Substring(0,6) == childIDmanual.ToUpper().Substring(1,6)`<br>M1 Not the same code ! - Please verify !	TEXT childIDverification1<br>..
VARIABLE<br>`IsAnswered(childIDbarcode) ? childIDbarcode : childIDmanual`	STRING childID
VARIABLE<br>`IsAnswered(childIDmanual) ? childIDverification1.ToUpper().Substring(0,6) == childIDmanual.ToUpper().Substring(1,6) : true /* put existing ID check here */`	BOOLEAN IDok
VARIABLE<br>`(childID.ToCharArray()[1] - 48)*10 + (childID.ToCharArray()[2] - 48)`	LONG s
VARIABLE<br>`(childID.ToCharArray()[4] - 48)*10 + (childID.ToCharArray()[5] - 48)`	LONG f
VARIABLE<br>`new int[]{0,1,2,3,4,5,999,6,999,7,8,999,9,10,999,11,999,12,999,999,13,999,14,999,15,999}[(childID.ToUpper().ToCharArray()[3] - 65)]`	LONG l
VARIABLE<br>`(f<=30)? /* B01A01-B99Y30 */ l*10+(f-1)*160+(s-1)/10 +1 : /* B01A31-B97F42 */ l*10+(f-30-1)*160+(s-1)/10 +1`	LONG rc
VARIABLE<br>`(s-1)%10 +1`	LONG c

VARIABLE<br>`0 /* (f<=30) ? new int[]{ (int)lut[(int)rc].d1, (int)lut [(int)rc].d2, (int)lut[(int)rc].d3, (int)lut[(int)rc].d4 , (int)lut[(int)rc].d5, (int)lut[(int)rc].d6, (int)lut[( int`<br><br>And 400 other symbols [3]	DOUBLE `tlix`
VARIABLE<br>`(childID.ToCharArray()[1] - 48)*100000 + (childID.ToChar Array()[2] - 48)*10000 + (childID.ToCharArray()[3] - 48) *1000 + (childID.ToCharArray()[4] - 48)*100 + (childID.T oCharArray()[5] - 48)*10 + (chil`<br><br>And 141 other symbols [4]	LONG `idcode`
VARIABLE<br>`lut[(int)idcode].letter == 0 ? "VV" : lut[(int)idcode].l etter == 1 ? "II" : null`	STRING `TL`

STATIC TEXT

E `TL == null && (IsAnswered(childIDbarcode) || IsAnswered(childIDmanual))`

Invalid ID! Please verify!

Secteur of residence	SINGLE-SELECT `secteur`<br>01 ○ Secteur 1<br>02 ○ Secteur 2<br>03 ○ Secteur 3<br>04 ○ Secteur 4<br>05 ○ Secteur 5<br>06 ○ Secteur 6<br>07 ○ Secteur 7

BASELINE INFORMATION

FEEDING PRACTICES

E `IsAnswered(childIDmode)`

Is the child currently breastfeeding?	SINGLE-SELECT `breastFeeding`<br>01 ○ Yes<br>02 ○ No
Is the breastfeeding exclusive?<br>E `breastFeeding==1`	SINGLE-SELECT `breastFeedingExclusive`<br>01 ○ Yes<br>02 ○ No

BASELINE INFORMATION

MATERNAL SURVEY

E `IsAnswered(childIDmode)`

Mother’s age (years)<br>V1 `self.InRange(9,65)`<br>M1 This is not a likely age for a mother of a small child!	NUMERIC: INTEGER `motherAge`<br>_ _
Is the mother literate?	SINGLE-SELECT `motherLiterate`<br>01 ○ Yes<br>02 ○ No

What is the mother's level of education?	SINGLE-SELECT `motherEdu`<br>01 ○ None<br>02 ○ Primary<br>03 ○ Secondary<br>04 ○ Tertiary or above
How many times has the mother been pregnant?	SINGLE-SELECT `preg`<br>01 ○ Enter number<br>02 ○ Don't know
Number of times pregnant<br>I Do not include this child<br>E `preg == 1`<br>W1 `self.InRange(0,100)`<br>M1 Please check this number!	NUMERIC: INTEGER `pregNum`<br>_ _
How many live children does the mother have?<br>I Do not include this child!<br>E `preg==1`	NUMERIC: INTEGER `numberLiveChildren`<br>_ _

STATIC TEXT

E `IsAnswered(preg)`

Please write I on the %childFirstname%'s bracelet with the timepoint and send %childFirstname% to anthropometry station

Basic information

Title 3-GAMIN - Anthropometrie

ANTHROPOMETRY

Tap to record the time	DATE: CURRENT TIME `startTime`
VARIABLE<br>`startTime.Value.Year`	LONG `startYear`
Which visit?<br>W1 `false`<br>M1 You selected %self% timepoint. Are you sure?	SINGLE-SELECT `timepoint`<br>01 ○ Baseline<br>02 ○ 14-day<br>03 ○ 6-month
Please enter the child's first name	TEXT `childFirstname`
VARIABLE<br>`"blue"`	STRING `colorName`
Birthdate - Year<br>V1 `self.InRange(1900, startYear) || self==9999`<br>M1 Must be between 1900 and %startYear%	NUMERIC: INTEGER `yearN`<br>SPECIAL VALUES<br>9999 Don't know
Birthdate - Month<br>E `yearN.InRange(1900,startYear)`	SINGLE-SELECT `monthN`<br>01 ○ January<br>02 ○ February<br>03 ○ March<br>04 ○ April<br>05 ○ May<br>06 ○ June<br>07 ○ July<br>08 ○ August<br>09 ○ September<br>10 ○ October<br>11 ○ November<br>12 ○ December<br>99 ○ Don't know

**Birthdate - Day**<br>F (@optioncode==30 && monthN!=2) || (@optioncode==31 && monthN.InList(1,3,5,7,8,10,12)) || @optioncode.InRange(1,29) || @optioncode==99<br>E `monthN.InRange(1,12)`	SINGLE-SELECT — `dayN`<br>01 ○ 1<br>02 ○ 2<br>03 ○ 3<br>04 ○ 4<br>05 ○ 5<br>06 ○ 6<br>07 ○ 7<br>08 ○ 8<br>09 ○ 9<br>10 ○ 10<br>11 ○ 11<br>12 ○ 12<br>13 ○ 13<br>14 ○ 14<br>15 ○ 15<br>16 ○ 16<br>And 16 other symbols [1]
**Reported age in years or months?**<br>E `yearN==9999`	SINGLE-SELECT — `reportedAgeMetric`<br>01 ○ Years<br>02 ○ Months
**Age de %childFirstname%:**<br>E `yearN==9999`<br>V1 `reportedAgeMetric==1 ? self < 6 : true`<br>M1 Child must be younger than 6 years old!<br>V2 `reportedAgeMetric==2 ? self < 60 : true`<br>M2 Child must be younger than 60 months old!	NUMERIC: INTEGER — `reportedAge`<br>_ _
VARIABLE<br>`/* if day of month unknown use 15th -> max.error 2 weeks */ (IsAnswered(reportedAge) && reportedAgeMetric==1) ? ((long)reportedAge * 365) : (IsAnswered(reportedAge) && reportedAgeMetric==2) ? ((`<br>And 433 other symbols [1]	LONG — `ageInDays`
VARIABLE<br>`IsAnswered(reportedAge) && reportedAgeMetric==1 ? (long) reportedAge*12 : IsAnswered(reportedAge) && reportedAgeMetric==2 ? (long) reportedAge : CenturyMonthCode(startTime.Value.Month, startTime.Value`<br>And 205 other symbols [2]	LONG — `ageInMonths`
VARIABLE<br>`ageInDays/365`	DOUBLE — `ageInYears`

STATIC TEXT

E `ageInDays<31 && ageInDays>0`

%childFirstname% is %ageInDays% days old

STATIC TEXT

E `ageInDays>=31 && ageInDays<=365`

%childFirstname% is %ageInMonths% months old

STATIC TEXT

E `ageInDays>365`

%childFirstname% is %ageInYears% years old

STATIC TEXT

E `ageInDays >= 2190`

Are you sure? This child cannot be 6 years or older!

STATIC TEXT

E `(ageInDays>0 && ageInDays<180) && timepoint==4`

Cannot be younger than 6 months at 6-month follow up!

What is %childFirstname%'s sex?	SINGLE-SELECT<br>01 ○ Male<br>02 ○ Female	childSex
%childFirstname%'s ID number	SINGLE-SELECT<br>01 ○ Scan barcode<br>02 ○ Enter manually	childIDmode
Scan %childFirstname%'s ID<br>E `childIDmode==1`<br>V1 `self.ToUpper().ToCharArray()[0]=='L'`<br>M1 Invalid ID !<br>V2 `TL!=null`<br>M2 Invalid ID !	BARCODE	childIDbarcode
Enter %childFirstname%'s ID<br>E `childIDmode==2`<br>V1 `self.ToUpper().ToCharArray()[0]=='L'`<br>M1 Invalid ID !<br>V2 `TL!=null`<br>M2 Invalid ID!	TEXT<br>..	childIDmanual

ANTHROPOMETRY

VERIFICATION

E `IsAnswered(childIDmanual) && TL!=null`

Enter the letter in the code<br>V1 `self.ToUpper().Substring(0,1) == childIDmanual.ToUpper().Substring(0,1)`<br>M1 Not the same code ! - Please verify !	TEXT<br>..	childIDverification
Enter the numbers in the code<br>V1 `self.ToUpper().Substring(0,6) == childIDmanual.ToUpper().Substring(1,6)`<br>M1 Not the same code ! - Please verify !	TEXT<br>..	childIDverification1
VARIABLE<br>`IsAnswered(childIDbarcode) ? childIDbarcode : childIDmanual`	STRING	childID
VARIABLE<br>`IsAnswered(childIDmanual) ? childIDverification1.ToUpper().Substring(0,6) == childIDmanual.ToUpper().Substring(1,6) : true /* put existing ID check here */`	BOOLEAN	IDok
VARIABLE<br>`(childID.ToCharArray()[1] - 48)*10 + (childID.ToCharArray()[2] - 48)`	LONG	s
VARIABLE<br>`(childID.ToCharArray()[4] - 48)*10 + (childID.ToCharArray()[5] - 48)`	LONG	f

VARIABLE<br>`new int[]{0,1,2,3,4,5,999,6,999,7,8,999,9,10,999,11,999,12,999,999,13,999,14,999,15,999}[(childID.ToUpper().ToCharArray()[3] - 65)]`	LONG l
VARIABLE<br>`(f<=30)? /* B01A01-B99Y30 */ l*10+(f-1)*160+(s-1)/10 +1 : /* B01A31-B97F42 */ l*10+(f-30-1)*160+(s-1)/10 +1`	LONG rc
VARIABLE<br>`(s-1)%10 +1`	LONG c
VARIABLE<br>`0 /* (f<=30) ? new int[]{ (int)lut[(int)rc].d1, (int)lut[(int)rc].d2, (int)lut[(int)rc].d3, (int)lut[(int)rc].d4, (int)lut[(int)rc].d5, (int)lut[(int)rc].d6, (int)lut[(int` <br>And 400 other symbols [3]	DOUBLE tlix
VARIABLE<br>`(childID.ToCharArray()[1] - 48)*100000 + (childID.ToCharArray()[2] - 48)*10000 + (childID.ToCharArray()[3] - 48)*1000 + (childID.ToCharArray()[4] - 48)*100 + (childID.ToCharArray()[5] - 48)*10 + (chil`<br><br>And 141 other symbols [4]	LONG idcode
VARIABLE<br>`lut[(int)idcode].letter == 0 ? "VV" : lut[(int)idcode].letter == 1 ? "II" : null`	STRING TL

STATIC TEXT

E `TL == null && (IsAnswered(childIDbarcode) || IsAnswered(childIDmanual))`

Invalid ID! Please verify!

Height or length measured?	SINGLE-SELECT metric_height<br>01 ○ Height<br>02 ○ Length

ANTHROPOMETRY

Roster: %METRIC_HEIGHT% MEASUREMENT

generated by fixed list R_height

01 1

02 2

03 3

%childFirstname%'s %metric_height% (cm)<br>V1 `@rowcode==2?self.InRange(R_height[1].height_m*0.9,R_height[1].height_m*1.1):true`<br>M1 The two measurements are very different! Please verify!<br>V2 `@rowcode==3?self.InRange(R_height[1].height_m*0.9,R_height[1].height_m*1.1) && self.InRange(R_height[2].height_m*0.9,R_height[2].height_m*1.1):true`<br>M2 The 3 measurements differ too much. Please verify!<br>V3 `self.InRange(0,160)`<br>M3 This cannot be the height in cm!<br>V4 `@rowcode==3 ? self!=(R_height[2].height_m + R_height[1].height_m)/2:true`<br>M4 All 3 heights should not be exact same!	NUMERIC: DECIMAL height_m<br>_ _
VARIABLE<br>`R_height.Sum(x => x.height_m) / 3`	DOUBLE height

STATIC TEXT

%metric_height% (average) = %height%

%childFirstname%'s weight<br>V1 `self.InRange(1,20)`<br>M1 This can't be the weight in lbs or kg!	NUMERIC: DECIMAL `weight`<br>_ .
Which units?<br>E `IsAnswered(weight)`	SINGLE-SELECT `weightUnit`<br>01 ○ kg<br>02 ○ lbs
MUAC in cm<br>V1 `self>0 && self<20`<br>M1 This cannot be %childFirstname%'s MUAC in centimeters!	NUMERIC: DECIMAL `MUAC`<br>_ .
Select signs of Malnutrition	MULTI-SELECT: YES/NO `malnutritionSigns`<br>01 ☐ / ☐ Pedal oedema<br>02 ☐ / ☐ Kwashiorkor<br>03 ☐ / ☐ Marasmus

STATIC TEXT

E `(MUAC < 11.5 && ageInDays > 182) || (malnutritionSigns.Yes.ContainsAny(1,2,3))`

Refer %childFirstname% to the clinic!

STATIC TEXT

E `IsAnswered(MUAC)`

Please write %childFirstname%'s weight on the bracelet and the letter A and make sure %childFirstname% is sent to blood smear station!

Basic information

Title 4-GAMIN - Ecouvillons

TYPE OF SWAB

Tap to record the time	DATE: CURRENT TIME<br>..	startTime
Is this an air swab	SINGLE-SELECT<br>01 ○ yes<br>02 ○ no	airswab

VARIABLE<br>(childID.ToCharArray()[1] - 48)*10 + (childID.ToCharArra y()[2] - 48)	LONG	s
VARIABLE<br>(childID.ToCharArray()[4] - 48)*10 + (childID.ToCharArra y()[5] - 48)	LONG	f
VARIABLE<br>new int[]{0,1,2,3,4,5,999,6,999,7,8,999,9,10,999,11,999, 12,999,999,13,999,14,999,15,999}[(childID.ToUpper().ToCh arArray()[3] - 65)]	LONG	l
VARIABLE<br>(f<=30)? /* B01A01-B99Y30 */ l*10+(f-1)*160+(s-1)/10 +1 : /* B01A31-B97F42 */ l*10+(f-30-1)*160+(s-1)/10 +1	LONG	rc
VARIABLE<br>(s-1)%10 +1	LONG	c
VARIABLE<br>0 /* (f<=30) ? new int[]{ (int)lut[(int)rc].d1, (int)lut [(int)rc].d2, (int)lut[(int)rc].d3, (int)lut[(int)rc].d4 , (int)lut[(int)rc].d5, (int)lut[(int)rc].d6, (int)lut[(int<br>And 400 other symbols [1]	DOUBLE	tlix
VARIABLE<br>(childID.ToCharArray()[1] - 48)*100000 + (childID.ToChar Array()[2] - 48)*10000 + (childID.ToCharArray()[3] - 48) *1000 + (childID.ToCharArray()[4] - 48)*100 + (childID.T oCharArray()[5] - 48)*10 + (chil<br>And 141 other symbols [2]	LONG	idcode
VARIABLE<br>lut[(int)idcode].letter == 0 ? "VV" : lut[(int)idcode].l etter == 1 ? "II" : null	STRING	TL

STATIC TEXT

E TL == null && (IsAnswered(childIDbarcode) \|\| IsAnswered(childIDmanual))

Invalid ID! Please verify!

Record Nasopharyngeal swab 1 - ID number	SINGLE-SELECT<br>01 ○ Scan barcode<br>02 ○ Enter manually<br>03 ○ Sample cannot be collected	NPswab1IDmode
Please specify why the sample cannot be collected<br>E NPswab1IDmode==3	TEXT<br>..	whynpswab1
Scan the NP sample's ID number<br>E NPswab1IDmode==1<br>V1 in1np1==true||in2np1==true||in3np1==true||in4np1==true||in5np1==true||in6np1==true||in7np1==true||in8np1==true||in9np1==true||in10np1==true||in11np1==true||in12np1==true||in13np1==true||in14np1==true| And 14 other symbols [1]<br>M1 This is not a valid ID for NP swab!	BARCODE	NPswab1IDscanned
Enter the NP sample's ID number<br>E NPswab1IDmode==2<br>V1 in1np1==true||in2np1==true||in3np1==true||in4np1==true||in5np1==true||in6np1==true||in7np1==true||in8np1==true||in9np1==true||in10np1==true||in11np1==true||in12np1==true||in13np1==true||in14np1==true| And 14 other symbols [2]<br>M1 This is not a valid ID for NP swab!	TEXT<br>..	NPswab1IDmanual

NASOPHARYNGEAL + RECTAL SWABS

VERIFICATION

E `IsAnswered(NPswab1IDmanual)`

**Enter the letters in the code**<br><br>V1 `self.ToUpper().Substring(0,2) == NPswab1IDmanual.ToUpper().Substring(0,2)`<br>M1 Not the same code ! - Please verify !	TEXT<br>..	`npswab1IDverification`
**Enter the numbers in the code**<br><br>V1 `self.ToUpper().Substring(0,5) == NPswab1IDmanual.ToUpper().Substring(2,5)`<br>M1 Not the same code ! - Please verify !	TEXT<br>..	`npswab1IDverification1`
VARIABLE<br>`IsAnswered(NPswab1IDscanned) ? NPswab1IDscanned : NPswab1IDmanual`	STRING	`NPswab1ID`

NASOPHARYNGEAL + RECTAL SWABS

ID %NPSWAB1ID% PROCESSED

VARIABLE<br>`(NPswab1ID.ToCharArray()[2] - 48)*10000 + (NPswab1ID.ToCharArray()[3] - 48)*1000 + (NPswab1ID.ToCharArray()[4] - 48)*100 + (NPswab1ID.ToCharArray()[5] - 48)*10 + (NPswab1ID.ToCharArray()[6] - 48)`	LONG	`numsidnp1`
VARIABLE<br>`Array.IndexOf( new long[]{ 23195, 79946, 58474, 81583, 59277, 76440, 46404, 50259, 52854, 23109, 27398, 56455, 30793, 9710, 53464, 52062, 49471, 71029, 13090, 9955, 15787, 14966, 73376, 3547, 64755, 7`<br><br>And 6719 other symbols [3]	BOOLEAN	`in1np1`
VARIABLE<br>`Array.IndexOf( new long[]{ 51235, 44393, 28865, 31710, 16494, 17237, 13607, 269, 69458, 49587, 23826, 23990, 84499, 66954, 36547, 24272, 55520, 2940, 45945, 83807, 80588, 41186, 4658, 79496, 69256, 55`<br><br>And 6689 other symbols [4]	BOOLEAN	`in2np1`
VARIABLE<br>`Array.IndexOf( new long[]{ 3165, 63325, 74969, 13081, 68988, 22549, 5328, 71176, 47262, 36591, 43495, 42984, 66738, 86561, 67504, 35607, 56203, 74293, 73675, 24036, 78320, 16480, 34148, 18592, 21747,`<br><br>And 6737 other symbols [5]	BOOLEAN	`in3np1`
VARIABLE<br>`Array.IndexOf( new long[]{ 21343, 49101, 26119, 44793, 32063, 57969, 31249, 64495, 21704, 88802, 57142, 72327, 14821, 47773, 33809, 9567, 41689, 88379, 40275, 10356, 83, 29933, 73221, 9339, 42328, 176`<br><br>And 6706 other symbols [6]	BOOLEAN	`in4np1`
VARIABLE<br>`Array.IndexOf( new long[]{ 11451, 74106, 65878, 1418, 37605, 59178, 66925, 69116, 9308, 62690, 69580, 27903, 24814, 55910, 28666, 59172, 6977, 2814, 72963, 37714, 37396, 25906, 40810, 8049, 18155, 317`<br><br>And 6729 other symbols [7]	BOOLEAN	`in5np1`

VARIABLE<br>`Array.IndexOf( new long[]{ 33193, 77693, 26, 76569, 63642, 62218, 82016, 8067, 67108, 27316, 23784, 64167, 66242, 54657, 60254, 4716, 58435, 34133, 35615, 40784, 64608, 25239, 7663, 43524, 13849, 6256`<br><br>And 6738 other symbols [17]	BOOLEAN `in15np1`
Record Nasopharyngeal swab 2 - ID number	SINGLE-SELECT `NPswab2IDmode`<br>01 ○ Scan barcode<br>02 ○ Enter manually<br>03 ○ Sample cannot be collected
Please specify why the sample cannot be collected<br><br>E `NPswab2IDmode==3`	TEXT `whynpswab2`<br>..
Scan the second NP sample's ID number<br><br>E `NPswab2IDmode==1`<br>V1 `NPswab1ID!=NPswab2ID`<br>M1 The second sample cannot have the same ID as the first one!<br>V2 `in1np2==true||in2np2==true||in3np2==true||in4np2==true||in5np2==true||in6np2==true||in7np2==true||in8np2==true||in9np2==true||in10np2==true||in11np2==true||in12np2==true||in13np2==true||in14np2==true|` And 14 other symbols [3]<br>M2 This is not a valid NP swab sample ID!	BARCODE `NPswab2IDscanned`
Enter the second NP sample's ID number<br><br>E `NPswab2IDmode==2`<br>V1 `NPswab1ID!=NPswab2ID`<br>M1 The second sample cannot have the same ID as the first one!<br>V2 `in1np2==true||in2np2==true||in3np2==true||in4np2==true||in5np2==true||in6np2==true||in7np2==true||in8np2==true||in9np2==true||in10np2==true||in11np2==true||in12np2==true||in13np2==true||in14np2==true|` And 14 other symbols [4]<br>M2 This is not a valid NP sample ID!	TEXT `NPswab2IDmanual`<br>..

NASOPHARYNGEAL + RECTAL SWABS

VERIFICATION

E `IsAnswered(NPswab2IDmanual)`

Enter the letters in the code<br><br>V1 `self.ToUpper().Substring(0,2) == NPswab2IDmanual.ToUpper().Substring(0,2)`<br>M1 Not the same code ! - Please verify !	TEXT `npswab2IDverification`<br>..
Enter the numbers in the code<br><br>V1 `self.ToUpper().Substring(0,5) == NPswab2IDmanual.ToUpper().Substring(2,5)`<br>M1 Not the same code ! - Please verify !	TEXT `npswab2IDverification1`<br>..
VARIABLE<br>`IsAnswered(NPswab2IDscanned) ? NPswab2IDscanned : NPswab2IDmanual`	STRING `NPswab2ID`

NASOPHARYNGEAL + RECTAL SWABS

ID %NPSWAB2ID% PROCESSED

VARIABLE<br>`Array.IndexOf( new long[]{ 50077, 31828, 61990, 3458, 51786, 83142, 30192, 85989, 58932, 10371, 56181, 20885, 14417, 22639, 8167, 33254, 26719, 20704, 87801, 76378, 34188, 25548, 56187, 56010, 80102,`<br><br>And 6693 other symbols [27]	BOOLEAN	in10np2
VARIABLE<br>`Array.IndexOf( new long[]{ 68190, 75951, 59725, 13823, 85694, 62486, 63690, 69932, 49017, 4730, 84014, 32101, 74479, 13928, 31744, 48491, 79683, 74958, 55364, 58976, 18179, 54025, 73038, 16246, 11230,`<br><br>And 6728 other symbols [28]	BOOLEAN	in11np2
VARIABLE<br>`Array.IndexOf( new long[]{ 30332, 12132, 17673, 22187, 29150, 12182, 80874, 87886, 30829, 76608, 73316, 82758, 72274, 76274, 42300, 68221, 31766, 72123, 18153, 47368, 39193, 19423, 7887, 31936, 17706,`<br><br>And 6735 other symbols [29]	BOOLEAN	in12np2
VARIABLE<br>`Array.IndexOf( new long[]{ 51105, 14380, 16610, 29489, 70642, 1662, 50501, 2951, 55693, 81628, 13982, 49750, 31934, 14891, 4724, 61442, 38523, 32423, 83496, 84155, 60598, 72571, 82625, 41582, 62338, 4`<br><br>And 6714 other symbols [30]	BOOLEAN	in13np2
VARIABLE<br>`Array.IndexOf( new long[]{ 59095, 50322, 83827, 52210, 11718, 40118, 54068, 77342, 83728, 5387, 30670, 4293, 44828, 63204, 79315, 48204, 16795, 48717, 71088, 36017, 37207, 69983, 75080, 19425, 75351,`<br><br>And 6745 other symbols [31]	BOOLEAN	in14np2
VARIABLE<br>`Array.IndexOf( new long[]{ 33193, 77693, 26, 76569, 63642, 62218, 82016, 8067, 67108, 27316, 23784, 64167, 66242, 54657, 60254, 4716, 58435, 34133, 35615, 40784, 64608, 25239, 7663, 43524, 13849, 6256`<br><br>And 6738 other symbols [32]	BOOLEAN	in15np2

STATIC TEXT

E `IsAnswered(NPswab2ID) && NPswab1ID==NPswab2ID && false`

The second sample cannot have the same ID as the first one!

Record rectal swab 1 - ID number	SINGLE-SELECT<br>01 ○ Scan barcode<br>02 ○ Enter manually<br>03 ○ Sample cannot be collected	rectalSwab1IDmode
Please specify why the sample cannot be collected<br><br>E `rectalSwab1IDmode==3`	TEXT<br>..	whyrectalswab1
Scan the rectal sample's ID number<br><br>E `rectalSwab1IDmode==1`<br>V1 `in1r1==true||in2r1==true||in3r1==true||in4r1==true||in5r1==true||in6r1==true||in7r1==true||in8r1==true||in9r1==true||in10r1==true||in11r1==true||in12r1==true||in13r1==true||in14r1==true||in15r1==true`<br>M1 This is not a valid rectal sample ID!<br>V2 `rectalSwab1ID!=NPswab2ID`<br>M2 The rectal sample cannot have the same ID as the NP samples!	BARCODE	rectalSwab1IDscanned

Enter the rectal sample's ID number	TEXT rectalSwab1IDmanual
E `rectalSwab1IDmode==2`<br>V1 `in1r1==true||in2r1==true||in3r1==true||in4r1==true||in5r1==true||in6r1==true||in7r1==true||in8r1==true||in9r1==true||in10r1==true||in11r1==true||in12r1==true||in13r1==true||in14r1==true||in15r1==true`<br>M1 This is not a valid rectal sample ID!<br>V2 `rectalSwab1ID!=NPswab2ID`<br>M2 The rectal sample cannot have the same ID as the NP samples!	....

NASOPHARYNGEAL + RECTAL SWABS

VERIFICATION

E `IsAnswered(rectalSwab1IDmanual)`

Enter the letters in the code	TEXT rectalSwab1IDverification
V1 `self.ToUpper().Substring(0,2) == rectalSwab1IDmanual.ToUpper().Substring(0,2)`<br>M1 Not the same code ! - Please verify !	....
**Enter the numbers in the code**	**TEXT rectalSwab1IDverification1**
V1 `self.ToUpper().Substring(0,5) == rectalSwab1IDmanual.ToUpper().Substring(2,5)`<br>M1 Not the same code ! - Please verify !	....
VARIABLE<br>`IsAnswered(rectalSwab1IDscanned) ? rectalSwab1IDscanned : rectalSwab1IDmanual`	STRING rectalSwab1ID

NASOPHARYNGEAL + RECTAL SWABS

ID %RECTALSWAB1ID% PROCESSED

VARIABLE<br>`(rectalSwab1ID.ToCharArray()[2] - 48)*10000 + (rectalSwab1ID.ToCharArray()[3] - 48)*1000 + (rectalSwab1ID.ToCharArray()[4] - 48)*100 + (rectalSwab1ID.ToCharArray()[5] - 48)*10 + (rectalSwab1ID.ToC`<br>And 20 other symbols [33]	LONG numsidr1
VARIABLE<br>`Array.IndexOf( new long[]{ 23195, 79946, 58474, 81583, 59277, 76440, 46404, 50259, 52854, 23109, 27398, 56455, 30793, 9710, 53464, 52062, 49471, 71029, 13090, 9955, 15787, 14966, 73376, 3547, 64755, 7`<br>And 6718 other symbols [34]	BOOLEAN in1r1
VARIABLE<br>`Array.IndexOf( new long[]{ 51235, 44393, 28865, 31710, 16494, 17237, 13607, 269, 69458, 49587, 23826, 23990, 84499, 66954, 36547, 24272, 55520, 2940, 45945, 83807, 80588, 41186, 4658, 79496, 69256, 55`<br>And 6688 other symbols [35]	BOOLEAN in2r1
VARIABLE<br>`Array.IndexOf( new long[]{ 3165, 63325, 74969, 13081, 68988, 22549, 5328, 71176, 47262, 36591, 43495, 42984, 66738, 86561, 67504, 35607, 56203, 74293, 73675, 24036, 78320, 16480, 34148, 18592, 21747,`<br>And 6736 other symbols [36]	BOOLEAN in3r1

VARIABLE<br>`Array.IndexOf( new long[]{ 51105, 14380, 16610, 29489, 70642, 1662, 50501, 2951, 55693, 81628, 13982, 49750, 31934, 14891, 4724, 61442, 38523, 32423, 83496, 84155, 60598, 72571, 82625, 41582, 62338, 4`<br><br>And 6713 other symbols [46]	BOOLEAN	in13r1
VARIABLE<br>`Array.IndexOf( new long[]{ 59095, 50322, 83827, 52210, 11718, 40118, 54068, 77342, 83728, 5387, 30670, 4293, 44828, 63204, 79315, 48204, 16795, 48717, 71088, 36017, 37207, 69983, 75080, 19425, 75351,`<br><br>And 6744 other symbols [47]	BOOLEAN	in14r1
VARIABLE<br>`Array.IndexOf( new long[]{ 33193, 77693, 26, 76569, 63642, 62218, 82016, 8067, 67108, 27316, 23784, 64167, 66242, 54657, 60254, 4716, 58435, 34133, 35615, 40784, 64608, 25239, 7663, 43524, 13849, 6256`<br><br>And 6737 other symbols [48]	BOOLEAN	in15r1

STATIC TEXT

E `IsAnswered(rectalSwab1IDmode)`

Please write E on %childFirstname%'s bracelet and continue to Venipuncture station!

AIR SWAB

E `airswab==1`

Record Nasopharyngeal swab 1 - ID number	SINGLE-SELECT NPswab1IDmodea<br>01 ○ Scan barcode<br>02 ○ Enter manually<br>03 ○ Sample cannot be collected
Please specify why the sample cannot be collected<br>E `NPswab1IDmodea==3`	TEXT whynpa1<br>..
Scan the NP sample's ID number<br>E `NPswab1IDmodea==1`<br>V1 `in1np1a==true||in2np1a==true||in3np1a==true||in4np1a==true||in5np1a==true||in6np1a==true||in7np1a==true||in8np1a==true||in9np1a==true||in10np1a==true||in11np1a==true||in12np1a==true||in13np1a==true||i` And 29 other symbols [5]<br>M1 This is not a NP sample ID!	BARCODE NPswab1IDscanneda
Enter the NP sample's ID number<br>E `NPswab1IDmodea==2`<br>V1 `in1np1a==true||in2np1a==true||in3np1a==true||in4np1a==true||in5np1a==true||in6np1a==true||in7np1a==true||in8np1a==true||in9np1a==true||in10np1a==true||in11np1a==true||in12np1a==true||in13np1a==true||i` And 29 other symbols [6]<br>M1 This is not a valid NP sample ID!	TEXT NPswab1IDmanuala<br>..

AIR SWAB

VERIFICATION

E `IsAnswered(NPswab1IDmanuala)`

Enter the letters in the code<br>W1 `self.ToUpper().Substring(0,2) == NPswab1IDmanuala.ToUpper().Substring(0,2)`<br>M1 Not the same code ! - Please verify !	TEXT npswab1IDverificationa<br>..
Enter the numbers in the code<br>V1 `self.ToUpper().Substring(0,5) == NPswab1IDmanuala.ToUpper().Substring(2,5)`<br>M1 Not the same code ! - Please verify !	TEXT npswab1IDverification1a<br>..
VARIABLE<br>`IsAnswered(NPswab1IDscanneda) ? NPswab1IDscanneda : NPswab1IDmanuala`	STRING NPswab1IDa

AIR SWAB

ID %NPSWAB1IDA% PROCESSED

VARIABLE<br>`(NPswab1IDa.ToCharArray()[2] - 48)*10000 + (NPswab1IDa.ToCharArray()[3] - 48)*1000 + (NPswab1IDa.ToCharArray()[4] - 48)*100 + (NPswab1IDa.ToCharArray()[5] - 48)*10 + (NPswab1IDa.ToCharArray()[6] -`<br>And 5 other symbols [49]	LONG numsidnp1a

VARIABLE<br>`Array.IndexOf( new long[]{ 50077, 31828, 61990, 3458, 51786, 83142, 30192, 85989, 58932, 10371, 56181, 20885, 14417, 22639, 8167, 33254, 26719, 20704, 87801, 76378, 34188, 25548, 56187, 56010, 80102,`<br><br>And 6694 other symbols [59]	BOOLEAN `in10np1a`
VARIABLE<br>`Array.IndexOf( new long[]{ 68190, 75951, 59725, 13823, 85694, 62486, 63690, 69932, 49017, 4730, 84014, 32101, 74479, 13928, 31744, 48491, 79683, 74958, 55364, 58976, 18179, 54025, 73038, 16246, 11230,`<br><br>And 6729 other symbols [60]	BOOLEAN `in11np1a`
VARIABLE<br>`Array.IndexOf( new long[]{ 30332, 12132, 17673, 22187, 29150, 12182, 80874, 87886, 30829, 76608, 73316, 82758, 72274, 76274, 42300, 68221, 31766, 72123, 18153, 47368, 39193, 19423, 7887, 31936, 17706,`<br><br>And 6736 other symbols [61]	BOOLEAN `in12np1a`
VARIABLE<br>`Array.IndexOf( new long[]{ 51105, 14380, 16610, 29489, 70642, 1662, 50501, 2951, 55693, 81628, 13982, 49750, 31934, 14891, 4724, 61442, 38523, 32423, 83496, 84155, 60598, 72571, 82625, 41582, 62338, 4`<br><br>And 6715 other symbols [62]	BOOLEAN `in13np1a`
VARIABLE<br>`Array.IndexOf( new long[]{ 59095, 50322, 83827, 52210, 11718, 40118, 54068, 77342, 83728, 5387, 30670, 4293, 44828, 63204, 79315, 48204, 16795, 48717, 71088, 36017, 37207, 69983, 75080, 19425, 75351,`<br><br>And 6746 other symbols [63]	BOOLEAN `in14np1a`
VARIABLE<br>`Array.IndexOf( new long[]{ 33193, 77693, 26, 76569, 63642, 62218, 82016, 8067, 67108, 27316, 23784, 64167, 66242, 54657, 60254, 4716, 58435, 34133, 35615, 40784, 64608, 25239, 7663, 43524, 13849, 6256`<br><br>And 6739 other symbols [64]	BOOLEAN `in15np1a`
Record Nasopharyngeal swab 2 - ID number	SINGLE-SELECT `NPswab2IDmodea`<br>01 ○ Scan barcode<br>02 ○ Enter manually<br>03 ○ Sample cannot be collected
Please specify why the sample cannot be collected<br><br>E `NPswab2IDmodea==3`	TEXT `whynpswab2a`<br>..
Scan the second NP sample's ID number<br><br>E `NPswab2IDmodea==1`<br>V1 `NPswab1IDa!=NPswab2IDa`<br>M1 The second sample cannot have the same ID as the first one!<br>V2 `in1np2a==true||in2np2a==true||in3np2a==true||in4np2a==true||in5np2a==true||in6np2a==true||in7np2a==true||in8np2a==true||in9np2a==true||in10np2a==true||in11np2a==true||in12np2a==true||in13np2a==true||i` And 29 other symbols [7]<br>M2 This is not a valid NP swab sample ID!	BARCODE `NPswab2IDscanneda`

**Enter the second NP sample's ID number**<br>E `NPswab2IDmodea==2`<br>V1 `NPswab1IDa!=NPswab2IDa`<br>M1 The second sample cannot have the same ID as the first one!<br>V2 `in1np2a==true||in2np2a==true||in3np2a==true||in4np2a==true||in5np2a==true||in6np2a==true||in7np2a==true||in8np2a==true||in9np2a==true||in10np2a==true||in11np2a==true||in12np2a==true||in13np2a==true||i` And 29 other symbols [8]<br>M2 This is not a valid NP sample ID!	TEXT `NPswab2IDmanuala`

AIR SWAB

VERIFICATION

E `IsAnswered(NPswab2IDmanuala)`

**Enter the letters in the code**<br>V1 `self.ToUpper().Substring(0,2) == NPswab2IDmanuala.ToUpper().Substring(0,2)`<br>M1 Not the same code ! - Please verify !	TEXT `npswab2IDverificationa`
**Enter the numbers in the code**<br>V1 `self.ToUpper().Substring(0,5) == NPswab2IDmanuala.ToUpper().Substring(2,5)`<br>M1 Not the same code ! - Please verify !	TEXT `npswab2IDverification1a`
VARIABLE<br>`IsAnswered(NPswab2IDscanneda) ? NPswab2IDscanneda : NPswab2IDmanuala`	STRING `NPswab2IDa`

AIR SWAB

ID %NPSWAB2IDA% PROCESSED

VARIABLE<br>`(NPswab2IDa.ToCharArray()[2] - 48)*10000 + (NPswab2IDa.ToCharArray()[3] - 48)*1000 + (NPswab2IDa.ToCharArray()[4] - 48)*100 + (NPswab2IDa.ToCharArray()[5] - 48)*10 + (NPswab2IDa.ToCharArray()[6] -`<br>And 5 other symbols [65]	LONG `numsidnp2a`
VARIABLE<br>`Array.IndexOf( new long[]{ 23195, 79946, 58474, 81583, 59277, 76440, 46404, 50259, 52854, 23109, 27398, 56455, 30793, 9710, 53464, 52062, 49471, 71029, 13090, 9955, 15787, 14966, 73376, 3547, 64755, 7`<br>And 6720 other symbols [66]	BOOLEAN `in1np2a`
VARIABLE<br>`Array.IndexOf( new long[]{ 51235, 44393, 28865, 31710, 16494, 17237, 13607, 269, 69458, 49587, 23826, 23990, 84499, 66954, 36547, 24272, 55520, 2940, 45945, 83807, 80588, 41186, 4658, 79496, 69256, 55`<br>And 6690 other symbols [67]	BOOLEAN `in2np2a`
VARIABLE<br>`Array.IndexOf( new long[]{ 3165, 63325, 74969, 13081, 68988, 22549, 5328, 71176, 47262, 36591, 43495, 42984, 66738, 86561, 67504, 35607, 56203, 74293, 73675, 24036, 78320, 16480, 34148, 18592, 21747,`<br>And 6738 other symbols [68]	BOOLEAN `in3np2a`

VARIABLE<br>`Array.IndexOf( new long[]{ 51105, 14380, 16610, 29489, 70642, 1662, 50501, 2951, 55693, 81628, 13982, 49750, 31934, 14891, 4724, 61442, 38523, 32423, 83496, 84155, 60598, 72571, 82625, 41582, 62338, 4`<br><br>And 6715 other symbols [78]	BOOLEAN	`in13np2a`
VARIABLE<br>`Array.IndexOf( new long[]{ 59095, 50322, 83827, 52210, 11718, 40118, 54068, 77342, 83728, 5387, 30670, 4293, 44828, 63204, 79315, 48204, 16795, 48717, 71088, 36017, 37207, 69983, 75080, 19425, 75351,`<br><br>And 6746 other symbols [79]	BOOLEAN	`in14np2a`
VARIABLE<br>`Array.IndexOf( new long[]{ 33193, 77693, 26, 76569, 63642, 62218, 82016, 8067, 67108, 27316, 23784, 64167, 66242, 54657, 60254, 4716, 58435, 34133, 35615, 40784, 64608, 25239, 7663, 43524, 13849, 6256`<br><br>And 6739 other symbols [80]	BOOLEAN	`in15np2a`

STATIC TEXT

E `IsAnswered(NPswab2IDa) && NPswab1IDa==NPswab2IDa && false`

The second sample cannot have the same ID as the first one!

Record rectal swab 1 - ID number	SINGLE-SELECT<br>01 ○ Scan barcode<br>02 ○ Enter manually<br>03 ○ Sample cannot be collected	`rectalSwab1IDmodea`
Please specify why the sample cannot be collected<br><br>E `rectalSwab1IDmodea==3`	TEXT<br>..	`whyrectala1`
Scan the rectal sample's ID number<br><br>E `rectalSwab1IDmodea==1`<br>V1 `in1r1a==true||in2r1a==true||in3r1a==true||in4r1a==true||in5r1a==true||in6r1a==true||in7r1a==true||in8r1a==true||in9r1a==true||in10r1a==true||in11r1a==true||in12r1a==true||in13r1a==true||in14r1a==true|` And 14 other symbols [9]<br>M1 This is not a valid rectal sample ID!<br>V2 `rectalSwab1IDa!=NPswab2IDa`<br>M2 The rectal sample cannot have the same ID as the NP samples!	BARCODE	`rectalSwab1IDscanneda`
Enter the rectal sample's ID number<br><br>E `rectalSwab1IDmodea==2`<br>V1 `in1r1a==true||in2r1a==true||in3r1a==true||in4r1a==true||in5r1a==true||in6r1a==true||in7r1a==true||in8r1a==true||in9r1a==true||in10r1a==true||in11r1a==true||in12r1a==true||in13r1a==true||in14r1a==true|` And 14 other symbols [10]<br>M1 This is not a valid rectal sample ID!<br>V2 `rectalSwab1IDa!=NPswab2IDa`<br>M2 The rectal sample cannot have the same ID as the NP samples!	TEXT<br>..	`rectalSwab1IDmanuala`

AIR SWAB

VERIFICATION

E `IsAnswered(rectalSwab1IDmanuala)`

Basic information

Title 5-GAMIN - Sang

BLOOD

Tap to record the time	DATE: CURRENT TIME `startTime`
VARIABLE<br>`startTime.Value.Year`	LONG `startYear`
Which visit?<br>W1 `false`<br>M1 You selected %self% timepoint. Are you sure?	SINGLE-SELECT `timepoint`<br>01 ○ Baseline<br>02 ○ 14-day<br>03 ○ 6-month
Please enter the child's first name	TEXT `childFirstname`
VARIABLE<br>`"blue"`	STRING `colorName`
Birthdate - Year<br>V1 `self.InRange(1900, startYear) || self==9999`<br>M1 Must be between 1900 and %startYear%	NUMERIC: INTEGER `yearN`<br>SPECIAL VALUES<br>9999 Don't know
Birthdate - Month<br>E `yearN.InRange(1900,startYear)`	SINGLE-SELECT `monthN`<br>01 ○ January<br>02 ○ February<br>03 ○ March<br>04 ○ April<br>05 ○ May<br>06 ○ June<br>07 ○ July<br>08 ○ August<br>09 ○ September<br>10 ○ October<br>11 ○ November<br>12 ○ December<br>99 ○ Don't know

**Birthdate - Day**<br>F (@optioncode==30 && monthN!=2) || (@optioncode==31 && monthN.InList(1,3,5,7,8,10,12)) || @optioncode.InRange(1,29) || @optioncode==99<br>E `monthN.InRange(1,12)`	SINGLE-SELECT — `dayN`<br>01 ○ 1<br>02 ○ 2<br>03 ○ 3<br>04 ○ 4<br>05 ○ 5<br>06 ○ 6<br>07 ○ 7<br>08 ○ 8<br>09 ○ 9<br>10 ○ 10<br>11 ○ 11<br>12 ○ 12<br>13 ○ 13<br>14 ○ 14<br>15 ○ 15<br>16 ○ 16<br>And 16 other symbols [1]
**Reported age in years or months?**<br>E `yearN==9999`	SINGLE-SELECT — `reportedAgeMetric`<br>01 ○ Years<br>02 ○ Months
**Age de %childFirstname%:**<br>E `yearN==9999`<br>V1 `reportedAgeMetric==1 ? self < 6 : true`<br>M1 Child must be younger than 6 years old!<br>V2 `reportedAgeMetric==2 ? self < 60 : true`<br>M2 Child must be younger than 60 months old!	NUMERIC: INTEGER — `reportedAge`<br>_ _
VARIABLE<br>`/* if day of month unknown use 15th -> max.error 2 weeks */ (IsAnswered(reportedAge) && reportedAgeMetric==1) ? ((long)reportedAge * 365) : (IsAnswered(reportedAge) && reportedAgeMetric==2) ? ((`<br>And 433 other symbols [1]	LONG — `ageInDays`
VARIABLE<br>`IsAnswered(reportedAge) && reportedAgeMetric==1 ? (long) reportedAge*12 : IsAnswered(reportedAge) && reportedAgeMetric==2 ? (long) reportedAge : CenturyMonthCode(startTime.Value.Month, startTime.Value`<br>And 205 other symbols [2]	LONG — `ageInMonths`
VARIABLE<br>`ageInDays/365`	DOUBLE — `ageInYears`

STATIC TEXT

E `ageInDays<31 && ageInDays>0`

%childFirstname% is %ageInDays% days old

STATIC TEXT

E `ageInDays>=31 && ageInDays<=365`

%childFirstname% is %ageInMonths% months old

STATIC TEXT

E `ageInDays>365`

%childFirstname% is %ageInYears% years old

STATIC TEXT

E `(timepoint==1 && ageInDays >= 2190) || (timepoint.InList(2,3) && ageInDays >=2370)`

Are you sure? This child cannot be 6 years or older!

STATIC TEXT

E `(ageInDays>0 && ageInDays<180) && timepoint==3`

Cannot be younger than 6 months at 6-month follow up!

What is %childFirstname%'s sex?	SINGLE-SELECT — childSex<br>01 ◯ Male<br>02 ◯ Female
%childFirstname%'s ID number	SINGLE-SELECT — childIDmode<br>01 ◯ Scan barcode<br>02 ◯ Enter manually
Scan %childFirstname%'s ID<br>E `childIDmode==1`<br>V1 `self.ToUpper().ToCharArray()[0]=='L'`<br>M1 Invalid ID !<br>V2 `TL!=null`<br>M2 Invalid ID !	BARCODE — childIDbarcode
Enter %childFirstname%'s ID<br>E `childIDmode==2`<br>V1 `self.ToUpper().ToCharArray()[0]=='L'`<br>M1 Invalid ID !<br>V2 `TL!=null`<br>M2 Invalid ID!	TEXT — childIDmanual<br>..

BLOOD

VERIFICATION

E `IsAnswered(childIDmanual) && TL!=null`

Enter the letter in the code<br>V1 `self.ToUpper().Substring(0,1) == childIDmanual.ToUpper().Substring(0,1)`<br>M1 Not the same code ! - Please verify !	TEXT — childIDverification<br>..
Enter the numbers in the code<br>V1 `self.ToUpper().Substring(0,6) == childIDmanual.ToUpper().Substring(1,6)`<br>M1 Not the same code ! - Please verify !	TEXT — childIDverification1<br>..
VARIABLE<br>`IsAnswered(childIDbarcode) ? childIDbarcode : childIDmanual`	STRING — childID
VARIABLE<br>`IsAnswered(childIDmanual) ? childIDverification1.ToUpper().Substring(0,6) == childIDmanual.ToUpper().Substring(1,6) : true /* put existing ID check here */`	BOOLEAN — IDok
VARIABLE<br>`(childID.ToCharArray()[1] - 48)*10 + (childID.ToCharArray()[2] - 48)`	LONG — s
VARIABLE<br>`(childID.ToCharArray()[4] - 48)*10 + (childID.ToCharArray()[5] - 48)`	LONG — f

VARIABLE<br>`new int[]{0,1,2,3,4,5,999,6,999,7,8,999,9,10,999,11,999,12,999,999,13,999,14,999,15,999}[(childID.ToUpper().ToCharArray()[3] - 65)]`	LONG — `l`
VARIABLE<br>`(f<=30)? /* B01A01-B99Y30 */ l*10+(f-1)*160+(s-1)/10 +1 : /* B01A31-B97F42 */ l*10+(f-30-1)*160+(s-1)/10 +1`	LONG — `rc`
VARIABLE<br>`(s-1)%10 +1`	LONG — `c`
VARIABLE<br>`0 /* (f<=30) ? new int[]{ (int)lut[(int)rc].d1, (int)lut[(int)rc].d2, (int)lut[(int)rc].d3, (int)lut[(int)rc].d4, (int)lut[(int)rc].d5, (int)lut[(int)rc].d6, (int)lut[(int` <br>And 400 other symbols [3]	DOUBLE — `tlix`
VARIABLE<br>`(childID.ToCharArray()[1] - 48)*100000 + (childID.ToCharArray()[2] - 48)*10000 + (childID.ToCharArray()[3] - 48)*1000 + (childID.ToCharArray()[4] - 48)*100 + (childID.ToCharArray()[5] - 48)*10 + (chil` <br>And 141 other symbols [4]	LONG — `idcode`
VARIABLE<br>`lut[(int)idcode].letter == 0 ? "VV" : lut[(int)idcode].letter == 1 ? "II" : null`	STRING — `TL`

STATIC TEXT

E `TL == null && (IsAnswered(childIDbarcode) || IsAnswered(childIDmanual))`

Invalid ID! Please verify!

Temperature metric<br><br>E `TL=="II" || TL=="VV"`	SINGLE-SELECT — `tempMetric`<br>01 ○ Celsius<br>02 ○ Fahrenheit
Please enter %childFirstname%'s temperature reading<br><br>E `IsAnswered(tempMetric)`<br>V1 `tempMetric==1 ? self>34 && self<44 : true`<br>M1 Are you sure?<br>V2 `tempMetric==2 ? self>93 && self<108 : true`<br>M2 Are you sure?	NUMERIC: DECIMAL — `childTemp`<br>_ _ _ _ _ _ _ _ _ _ _ _ _ _ _ _ _ _ _ _

STATIC TEXT

E `$feverTemp`

Give child an RDT

Do you want to give an RDT?<br><br>E `childTemp<=37.5`	SINGLE-SELECT — `giveRDT`<br>01 ○ Yes<br>02 ○ No
Was RDT administered?<br><br>E `$feverTemp || giveRDT==1`	SINGLE-SELECT — `rdtAdmin`<br>01 ○ Yes<br>02 ○ No
Why not?<br><br>E `rdtAdmin == 2`	TEXT — `rdtNoAdmin`<br>..

Results of RDT test<br>E `rdtAdmin == 1`	SINGLE-SELECT `rdtResults`<br>01 ○ Positive<br>02 ○ Negative<br>03 ○ Don't know

STATIC TEXT

E `rdtResults==1`

Please refer the kid to the nearest CSPS!

Record smear ID<br>E `TL=="II" || TL=="VV"`	SINGLE-SELECT `smearMode`<br>01 ○ Scan barcode<br>02 ○ Enter manually<br>03 ○ Smear cannot be collected
Please specify why the sample cannot be collected?<br>E `smearMode==3`	TEXT `whysmear`<br>..
Please scan the smear's ID number<br>E `smearMode==1`<br>V1 `in1s==true||in2s==true||in3s==true||in4s==true||in5s==true||in6s==true||in7s==true||in8s==true||in9s==true||in10s==true||in11s==true||in12s==true||in13s==true||in14s==true||in15s==true`<br>M1 This is not a valid smear ID!	BARCODE `smearIDscanned`
Please enter the smear's ID number<br>E `smearMode==2`<br>V1 `in1s==true||in2s==true||in3s==true||in4s==true||in5s==true||in6s==true||in7s==true||in8s==true||in9s==true||in10s==true||in11s==true||in12s==true||in13s==true||in14s==true||in15s==true`<br>M1 This is not a valid smear ID!	TEXT `smearIDmanual`<br>..
VARIABLE<br>`IsAnswered(smearIDscanned) ? smearIDscanned : smearIDmanual`	STRING `smearID`

BLOOD

VERIFICATION

E `IsAnswered(smearIDmanual)`

Enter the letters in the code<br>V1 `self.ToUpper().Substring(0,2) == smearIDmanual.ToUpper().Substring(0,2)`<br>M1 Not the same code ! - Please verify !	TEXT `smearIDverification`<br>..
Enter the numbers in the code<br>V1 `self.ToUpper().Substring(0,5) == smearIDmanual.ToUpper().Substring(2,5)`<br>M1 Not the same code ! - Please verify !	TEXT `smearIDverification1`<br>..

BLOOD

ID %SMEARID% PROCESSED

E `smearMode!=3`

VENIPUNCTURE

Streck tube ID number	SINGLE-SELECT `streckIDmodeV`<br>01 ○ Scan the code<br>02 ○ Code can't be scanned<br>03 ○ Sample cannot be collected
Scan streck tube ID<br>E `streckIDmodeV==1`<br>V1 `child_ID2[(int)numPartV].exists!=null`<br>M1 Invalid ID !	BARCODE `streckIDbarcodeV`
Enter streck tube ID<br>E `streckIDmodeV==2`<br>V1 `self.ToUpper().ToCharArray()[0]=='S' && self.ToUpper().ToCharArray()[1]=='A'`<br>M1 Invalid ID !<br>V2 `child_ID2[(int)numPartV].exists!=null`<br>M2 Invalid ID!	TEXT `streckIDmanualV`<br>..

VENIPUNCTURE

VERIFICATION

E `IsAnswered(streckIDmanualV) && child_ID2[(int)numPartV].exists!=null`

Enter the letters in the code<br>V1 `self.ToUpper().Substring(0,2) == streckIDmanualV.ToUpper().Substring(0,2)`<br>M1 Not the same code ! - Please verify !	TEXT `streckIDverificationV`<br>..
Enter the numbers in the code<br>V1 `self.ToUpper().Substring(0,5) == streckIDmanualV.ToUpper().Substring(2,5)`<br>M1 Not the same code ! - Please verify !	TEXT `streckIDverification1V`<br>..
VARIABLE<br>`IsAnswered(streckIDbarcodeV) ? streckIDbarcodeV : streckIDmanualV`	STRING `streckIDV`
VARIABLE<br>`(streckIDV.ToCharArray()[2] - 48)*10000 + (streckIDV.ToCharArray()[3] - 48)*1000 + (streckIDV.ToCharArray()[4] - 48)*100 + (streckIDV.ToCharArray()[5] - 48)*10 + (streckIDV.ToCharArray()[6] - 48)`	LONG `numPartV`
VARIABLE<br>`IsAnswered(streckIDmanualV) ? streckIDverification1V.ToUpper().Substring(0,6) == streckIDmanualV.ToUpper().Substring(1,6) : true /* put existing ID check here */`	BOOLEAN `IDokV`
VARIABLE<br>`(streckIDV.ToCharArray()[1] - 48)*10 + (streckIDV.ToCharArray()[2] - 48)`	LONG `sV`
VARIABLE<br>`(streckIDV.ToCharArray()[4] - 48)*10 + (streckIDV.ToCharArray()[5] - 48)`	LONG `fV`

VARIABLE<br>`new int[]{0,1,2,3,4,5,999,6,999,7,8,999,9,10,999,11,999,12,999,999,13,999,14,999,15,999}[(streckIDV.ToUpper().ToCharArray()[3] - 65)]`	LONG	lv
VARIABLE<br>`(fV<=30)? /* B01A01-B99Y30 */ lV*10+(fV-1)*160+(sV-1)/10 +1 : /* B01A31-B97F42 */ lV*10+(fV-30-1)*160+(sV-1)/10 + 1`	LONG	rcV
VARIABLE<br>`(sV-1)%10 +1`	LONG	cV
VARIABLE<br>`0 /* (f<=30) ? new int[]{ (int)lut[(int)rc].d1, (int)lut[(int)rc].d2, (int)lut[(int)rc].d3, (int)lut[(int)rc].d4, (int)lut[(int)rc].d5, (int)lut[(int)rc].d6, (int)lut[(int` <br>And 400 other symbols [20]	DOUBLE	tlixV
VARIABLE<br>`(streckIDV.ToCharArray()[1] - 48)*100000 + (streckIDV.ToCharArray()[2] - 48)*10000 + (streckIDV.ToCharArray()[3] - 48)*1000 + (streckIDV.ToCharArray()[4] - 48)*100 + (streckIDV.ToCharArray()[5] - 48)*`<br><br>And 153 other symbols [21]	LONG	idcodeV
VARIABLE<br>`lut[(int)idcodeV].letter == 0 ? "VV" : lut[(int)idcodeV].letter == 1 ? "II" : null`	STRING	TLV

STATIC TEXT

E `/*(child_ID[(int)numPart].exists == 1)?false:true*/ false`

Invalid ID! Please verify!

VARIABLE<br>`child_ID2[(int)numPartV].exists == 1`	BOOLEAN	dummy

STATIC TEXT

E `false`

%dummy%

How much blood did you collect?<br><br>E `streckIDmodeV.InList(1,2)`	SINGLE-SELECT<br>01 ○ Insufficient<br>02 ○ Less than half<br>03 ○ Half<br>04 ○ More than half<br>05 ○ Full	amountBlood
Why?<br><br>E `amountBlood == 1`	TEXT<br>..	amountBloodwhy
Did you collect at least 2 mL?<br><br>E `amountBlood==1 || amountBlood==2`	SINGLE-SELECT<br>01 ○ Yes<br>02 ○ No	amountBlood2ml

VENIPUNCTURE

Roster: CRYOTUBES

generated by fixed list

cryotubeN

01 Cryotube 1

02 Cryotube 2

03 Cryotube 3
04 Cryotube 4
05 Cryotube 5
06 Cryotube 6

Record cryotube - ID number<br>E `streckIDmodeV.InList(1,2)`	SINGLE-SELECT `tubeIDmode`<br>01 ○ Scan barcode<br>02 ○ Enter manually
Scan the cryotube sample's ID number<br>E `tubeIDmode==1`<br>V1 `self==streckIDV`<br>M1 Doesn't match streck tube ID. Please fix!<br>V2 `child_ID2[(int)numPartV].exists!=null`<br>M2 Invalid ID!	BARCODE `tubeIDscanned`
Enter the cryotube sample's ID number<br>E `tubeIDmode==2`<br>V1 `child_ID2[(int)numPartV].exists!=null`<br>M1 This is not a valid sample ID!<br>V2 `self==streckIDV`<br>M2 Doesn't match the streck tube ID. Please fix!	TEXT `tubeIDmanual`<br>..

VENIPUNCTURE / CRYOTUBES

VERIFICATION

E `IsAnswered(tubeIDmanual)`

Enter the letters in the code<br>V1 `self.ToUpper().Substring(0,2) == tubeIDmanual.ToUpper().Substring(0,2)`<br>M1 Not the same code ! - Please verify !	TEXT `tubeIDverification`<br>..
Enter the numbers in the code<br>V1 `self.ToUpper().Substring(0,5) == tubeIDmanual.ToUpper().Substring(2,5)`<br>M1 Not the same code ! - Please verify !	TEXT `tubeIDverification1`<br>..
VARIABLE<br>`IsAnswered(tubeIDscanned) ? tubeIDscanned : tubeIDmanual`	STRING `tubeID`

VENIPUNCTURE / CRYOTUBES

ID %TUBEID% PROCESSED

E `tubeID!=null`

STATIC TEXT

Please write S on %childFirstname%'s bracelet and continue to stool station

Basic information

Title 6-GAMIN - Selles Fraiches

FRESH FROZEN STOOL

Tap to record the time	DATE: CURRENT TIME `startTime`
VARIABLE<br>`startTime.Value.Year`	LONG `startYear`
Which visit?<br>W1 `false`<br>M1 You selected %self% timepoint. Are you sure?	SINGLE-SELECT `timepoint`<br>01 ○ Baseline<br>02 ○ 14-day<br>03 ○ 6-month
Please enter the child's first name	TEXT `childFirstname`
VARIABLE<br>`"blue"`	STRING `colorName`
Birthdate - Year<br>V1 `self.InRange(1900, startYear) || self==9999`<br>M1 Must be between 1900 and %startYear%	NUMERIC: INTEGER `yearN`<br>SPECIAL VALUES<br>9999 Don't know
Birthdate - Month<br>E `yearN.InRange(1900,startYear)`	SINGLE-SELECT `monthN`<br>01 ○ January<br>02 ○ February<br>03 ○ March<br>04 ○ April<br>05 ○ May<br>06 ○ June<br>07 ○ July<br>08 ○ August<br>09 ○ September<br>10 ○ October<br>11 ○ November<br>12 ○ December<br>99 ○ Don't know

**Birthdate - Day**<br>F (@optioncode==30 && monthN!=2) || (@optioncode==31 && monthN.InList(1,3,5,7,8,10,12)) || @optioncode.InRange(1,29) || @optioncode==99<br>E `monthN.InRange(1,12)`	SINGLE-SELECT `dayN`<br>01 ○ 1<br>02 ○ 2<br>03 ○ 3<br>04 ○ 4<br>05 ○ 5<br>06 ○ 6<br>07 ○ 7<br>08 ○ 8<br>09 ○ 9<br>10 ○ 10<br>11 ○ 11<br>12 ○ 12<br>13 ○ 13<br>14 ○ 14<br>15 ○ 15<br>16 ○ 16<br>And 16 other symbols [1]
**Reported age in years or months?**<br>E `yearN==9999`	SINGLE-SELECT `reportedAgeMetric`<br>01 ○ Years<br>02 ○ Months
**Age de %childFirstname%:**<br>E `yearN==9999`<br>V1 `reportedAgeMetric==1 ? self < 6 : true`<br>M1 Child must be younger than 6 years old!<br>V2 `reportedAgeMetric==2 ? self < 60 : true`<br>M2 Child must be younger than 60 months old!	NUMERIC: INTEGER `reportedAge`<br>_ _ _ _ _ _ _ _ _ _ _ _ _ _ _ _ _ _ _ _
VARIABLE<br>`/* if day of month unknown use 15th -> max.error 2 weeks */ (IsAnswered(reportedAge) && reportedAgeMetric==1) ? ((long)reportedAge * 365) : (IsAnswered(reportedAge) && reportedAgeMetric==2) ? ((`<br>And 433 other symbols [1]	LONG `ageInDays`
VARIABLE<br>`IsAnswered(reportedAge) && reportedAgeMetric==1 ? (long) reportedAge*12 : IsAnswered(reportedAge) && reportedAgeMetric==2 ? (long) reportedAge : CenturyMonthCode(startTime.Value.Month, startTime.Value`<br>And 205 other symbols [2]	LONG `ageInMonths`
VARIABLE<br>`ageInDays/365`	DOUBLE `ageInYears`

STATIC TEXT

E `ageInDays<31 && ageInDays>0`

%childFirstname% is %ageInDays% days old

STATIC TEXT

E `ageInDays>=31 && ageInDays<=365`

%childFirstname% is %ageInMonths% months old

STATIC TEXT

E `ageInDays>365`

%childFirstname% is %ageInYears% years old

STATIC TEXT

E `ageInDays >= 2190`

Are you sure? This child cannot be 6 years or older!

STATIC TEXT

E `(ageInDays>0 && ageInDays<180) && timepoint==4`

Cannot be younger than 6 months at 6-month follow up!

What is %childFirstname%'s sex?	SINGLE-SELECT<br>01 ○ Male<br>02 ○ Female	childSex
%childFirstname%'s ID number	SINGLE-SELECT<br>01 ○ Scan barcode<br>02 ○ Enter manually	childIDmode
Scan %childFirstname%'s ID<br>E `childIDmode==1`<br>V1 `self.ToUpper().ToCharArray()[0]=='L'`<br>M1 Invalid ID !<br>V2 `TL!=null`<br>M2 Invalid ID !	BARCODE	childIDbarcode
Enter %childFirstname%'s ID<br>E `childIDmode==2`<br>V1 `self.ToUpper().ToCharArray()[0]=='L'`<br>M1 Invalid ID !<br>V2 `TL!=null`<br>M2 Invalid ID!	TEXT<br>..	childIDmanual

FRESH FROZEN STOOL

VERIFICATION

E `IsAnswered(childIDmanual) && TL!=null`

Enter the letter in the code<br>V1 `self.ToUpper().Substring(0,1) == childIDmanual.ToUpper().Substring(0,1)`<br>M1 Not the same code ! - Please verify !	TEXT<br>..	childIDverification
Enter the numbers in the code<br>V1 `self.ToUpper().Substring(0,6) == childIDmanual.ToUpper().Substring(1,6)`<br>M1 Not the same code ! - Please verify !	TEXT<br>..	childIDverification1
VARIABLE<br>`IsAnswered(childIDbarcode) ? childIDbarcode : childIDmanual`	STRING	childID
VARIABLE<br>`IsAnswered(childIDmanual) ? childIDverification1.ToUpper().Substring(0,6) == childIDmanual.ToUpper().Substring(1,6) : true /* put existing ID check here */`	BOOLEAN	IDok
VARIABLE<br>`(childID.ToCharArray()[1] - 48)*10 + (childID.ToCharArray()[2] - 48)`	LONG	s
VARIABLE<br>`(childID.ToCharArray()[4] - 48)*10 + (childID.ToCharArray()[5] - 48)`	LONG	f

VARIABLE	LONG	l
`new int[]{0,1,2,3,4,5,999,6,999,7,8,999,9,10,999,11,999,12,999,999,13,999,14,999,15,999}[(childID.ToUpper().ToCharArray()[3] - 65)]`		

VARIABLE	LONG	rc
`(f<=30)? /* B01A01-B99Y30 */ l*10+(f-1)*160+(s-1)/10 +1 : /* B01A31-B97F42 */ l*10+(f-30-1)*160+(s-1)/10 +1`		

VARIABLE	LONG	c
`(s-1)%10 +1`		

VARIABLE	DOUBLE	tlix
`0 /* (f<=30) ? new int[]{ (int)lut[(int)rc].d1, (int)lut[(int)rc].d2, (int)lut[(int)rc].d3, (int)lut[(int)rc].d4, (int)lut[(int)rc].d5, (int)lut[(int)rc].d6, (int)lut[(int` And 400 other symbols [3]		

VARIABLE	LONG	idcode
`(childID.ToCharArray()[1] - 48)*100000 + (childID.ToCharArray()[2] - 48)*10000 + (childID.ToCharArray()[3] - 48)*1000 + (childID.ToCharArray()[4] - 48)*100 + (childID.ToCharArray()[5] - 48)*10 + (chil` And 141 other symbols [4]		

VARIABLE	STRING	TL
`lut[(int)idcode].letter == 0 ? "VV" : lut[(int)idcode].letter == 1 ? "II" : null`		

STATIC TEXT

E `TL == null && (IsAnswered(childIDbarcode) || IsAnswered(childIDmanual))`

Invalid ID! Please verify!

Question	Type / Options	Variable
When was the last time your child defecated?	SINGLE-SELECT<br>01 ○ Less than 6 hours ago<br>02 ○ More than 6 hours ago	defecTime
Were you able to collect stool sample?	SINGLE-SELECT<br>01 ○ Yes<br>02 ○ No	stoolTaken
Was the stool sample kept cold?<br>E `stoolTaken==1`	SINGLE-SELECT<br>01 ○ Yes<br>02 ○ No	stoolCold
Was the stool sample in enough quantity?<br>E `stoolTaken==1`	SINGLE-SELECT<br>01 ○ Yes<br>02 ○ No	stoolQuantity
Was the stool collected within 20 minutes of defecation?<br>E `stoolTaken==1`	SINGLE-SELECT<br>01 ○ Yes<br>02 ○ No	stoolTime
Record stool - ID number<br>E `stoolTaken==1`	SINGLE-SELECT<br>01 ○ Scan barcode<br>02 ○ Enter manually<br>03 ○ Sample cannot be collected	stoolIDmode
Please specify why the sample cannot be collected<br>E `stoolIDmode==3`	TEXT<br>..<br>44	whystool

**Scan the stool sample's ID number**<br>E `stoolIDmode==1`<br>V1 `in1r1==true||in2r1==true||in3r1==true||in4r1==true||in5r1==true||in6r1==true||in7r1==true||in8r1==true||in9r1==true||in10r1==true||in11r1==true||in12r1==true||in13r1==true||in14r1==true||in15r1==true`<br>M1 This is not a valid sample ID!	BARCODE `stoolIDscanned`
**Enter the stool sample's ID number**<br>E `stoolIDmode==2`<br>V1 `in1r1==true||in2r1==true||in3r1==true||in4r1==true||in5r1==true||in6r1==true||in7r1==true||in8r1==true||in9r1==true||in10r1==true||in11r1==true||in12r1==true||in13r1==true||in14r1==true||in15r1==true`<br>M1 This is not a valid sample ID!	TEXT `stoolIDmanual`<br>..

FRESH FROZEN STOOL

VERIFICATION

E `IsAnswered(stoolIDmanual)`

**Enter the letters in the code**<br>V1 `self.ToUpper().Substring(0,2) == stoolIDmanual.ToUpper().Substring(0,2)`<br>M1 Not the same code ! - Please verify !	TEXT `stoolIDverification`<br>..
**Enter the numbers in the code**<br>V1 `self.ToUpper().Substring(0,5) == stoolIDmanual.ToUpper().Substring(2,5)`<br>M1 Not the same code ! - Please verify !	TEXT `stoolIDverification1`<br>..
VARIABLE<br>`IsAnswered(stoolIDscanned) ? stoolIDscanned : stoolIDmanual`	STRING `stoolID`

FRESH FROZEN STOOL

ID %STOOLID% PROCESSED

E `stoolTaken==1`

VARIABLE<br>`(stoolID.ToCharArray()[2] - 48)*10000 + (stoolID.ToCharArray()[3] - 48)*1000 + (stoolID.ToCharArray()[4] - 48)*100 + (stoolID.ToCharArray()[5] - 48)*10 + (stoolID.ToCharArray()[6] - 48)`	LONG `numsidr1`
VARIABLE<br>`Array.IndexOf( new long[]{ 23195, 79946, 58474, 81583, 59277, 76440, 46404, 50259, 52854, 23109, 27398, 56455, 30793, 9710, 53464, 52062, 49471, 71029, 13090, 9955, 15787, 14966, 73376, 3547, 64755, 7`<br>And 6718 other symbols [5]	BOOLEAN `in1r1`
VARIABLE<br>`Array.IndexOf( new long[]{ 51235, 44393, 28865, 31710, 16494, 17237, 13607, 269, 69458, 49587, 23826, 23990, 84499, 66954, 36547, 24272, 55520, 2940, 45945, 83807, 80588, 41186, 4658, 79496, 69256, 55`<br>And 6688 other symbols [6]	BOOLEAN `in2r1`

Basic information

Title 7-GAMIN - Traitement

IDENTIFICATION

Tap to record the time	DATE: CURRENT TIME `startTime`
VARIABLE<br>`startTime.Value.Year`	LONG `startYear`
VARIABLE<br>`"blue"`	STRING `colorName`
What is the child's first name?	TEXT `childFirstname`
Birthdate - Year<br>V1 `self.InRange(1900, startYear) || self==9999`<br>M1 Must be between 1900 and %startYear%	NUMERIC: INTEGER `yearN`<br>SPECIAL VALUES<br>9999 Don't know
Birthdate - Month<br>E `yearN!=9999`	SINGLE-SELECT `monthN`<br>01 ○ January<br>02 ○ February<br>03 ○ March<br>04 ○ April<br>05 ○ May<br>06 ○ June<br>07 ○ July<br>08 ○ August<br>09 ○ September<br>10 ○ October<br>11 ○ November<br>12 ○ December<br>99 ○ Don't know
Birthdate - Day<br>F (@optioncode==30 && monthN!=2) || (@optioncode==31 && monthN.InList(1,3,5,7,8,10,12)) || @optioncode.InRange(1,29) || @optioncode ==99<br>E `monthN.InRange(1,12)`	SINGLE-SELECT `dayN`<br>01 ○ 1<br>02 ○ 2<br>03 ○ 3<br>04 ○ 4<br>05 ○ 5<br>06 ○ 6<br>07 ○ 7<br>08 ○ 8<br>09 ○ 9<br>10 ○ 10<br>11 ○ 11<br>12 ○ 12<br>13 ○ 13<br>14 ○ 14<br>15 ○ 15<br>16 ○ 16<br><br>And 16 other symbols [1] 47

Reported age in years or months?<br>E `yearN==9999`	SINGLE-SELECT — `reportedAgeMetric`<br>01 ○ Years<br>02 ○ Months
Age de %childFirstname%:<br>E `yearN==9999`<br>V1 `reportedAgeMetric==1 ? self < 5 : true`<br>M1 Child must be younger than 5 years old!<br>V2 `reportedAgeMetric==2 ? self < 60 : true`<br>M2 Child must be younger than 60 months old!	NUMERIC: INTEGER — `reportedAge`<br>_ _
VARIABLE<br>`/* if day of month unknown use 15th -> max.error 2 weeks */ (IsAnswered(reportedAge) && reportedAgeMetric==1) ? ((long)reportedAge * 365) : (IsAnswered(reportedAge) && reportedAgeMetric==2) ? ((`<br>And 433 other symbols [1]	LONG — `ageInDays`
VARIABLE<br>`IsAnswered(reportedAge) && reportedAgeMetric==1 ? (long) reportedAge*12 : IsAnswered(reportedAge) && reportedAgeMetric==2 ? (long) reportedAge : CenturyMonthCode(startTime.Value.Month, startTime.Value`<br>And 205 other symbols [2]	LONG — `ageInMonths`
VARIABLE<br>`ageInDays/365`	DOUBLE — `ageInYears`

STATIC TEXT

E `ageInDays<31 && ageInDays>0`

%childFirstname% is %ageInDays% days old

STATIC TEXT

E `ageInDays>=31 && ageInDays<=365`

%childFirstname% is %ageInMonths% months old

STATIC TEXT

E `ageInDays>365`

%childFirstname% is %ageInYears% years old

%childFirstname%'s ID number<br>E `(ageInDays>8 && ageInDays<=1825)`	SINGLE-SELECT — `childIDmode`<br>01 ○ Scan barcode<br>02 ○ Scan doesn't work
Scan %childFirstname%'s ID<br>E `childIDmode==1`<br>V1 `self.ToUpper().ToCharArray()[0]=='L'`<br>M1 Invalid ID !<br>V2 `TL!=null`<br>M2 Invalid ID!	BARCODE — `childIDbarcode`
Enter %childFirstname%'s ID<br>E `childIDmode==2`<br>V1 `self.ToUpper().ToCharArray()[0]=='L'`<br>M1 Invalid ID !<br>V2 `TL!=null`<br>M2 Invalid ID!	TEXT — `childIDmanual`<br>..

E `IsAnswered(childIDmanual) && TL!=null`

Question	Type / Variable
Enter the letter in the code<br><br>V1 `self.ToUpper().Substring(0,1) == childIDmanual.ToUpper().Substring(0,1)`<br>M1 Not the same code ! - Please verify !	TEXT `childIDverification`<br>..
Enter the numbers in the code<br><br>V1 `self.ToUpper().Substring(0,6) == childIDmanual.ToUpper().Substring(1,6)`<br>M1 Not the same code ! - Please verify !	TEXT `childIDverification1`<br>..
VARIABLE<br>`IsAnswered(childIDbarcode) ? childIDbarcode : childIDmanual`	STRING `childID`
VARIABLE<br>`IsAnswered(childIDmanual) ? childIDverification1.ToUpper().Substring(0,6) == childIDmanual.ToUpper().Substring(1,6) : true /* put existing ID check here */`	BOOLEAN `IDok`

STATIC TEXT

E `TL == null && (IsAnswered(childIDbarcode) || IsAnswered(childIDmanual))`

Invalid ID!

STATIC TEXT

E `!$eligible && (IsAnswered(reportedAge) || IsAnswered(yearN))`

%childFirstname% is not eligible for the study because %childFirstname% is not between 8 days and 59 months old.

Question	Type / Variable
What is %childFirstname%'s sex?<br><br>E `$eligible`	SINGLE-SELECT `childSex`<br>01 ○ Male<br>02 ○ Female
Consent to receive treatment?<br><br>E `TL != null && $eligible && IDok==true`	SINGLE-SELECT `consent`<br>01 ○ Yes<br>02 ○ No
VARIABLE<br>`(childID.ToCharArray()[1] - 48)*10 + (childID.ToCharArray()[2] - 48)`	LONG `s`
VARIABLE<br>`(childID.ToCharArray()[4] - 48)*10 + (childID.ToCharArray()[5] - 48)`	LONG `f`
VARIABLE<br>`new int[]{0,1,2,3,4,5,999,6,999,7,8,999,9,10,999,11,999,12,999,999,13,999,14,999,15,999}[(childID.ToUpper().ToCharArray()[3] - 65)]`	LONG `l`
VARIABLE<br>`(f<=30)? /* B01A01-B99Y30 */ l*10+(f-1)*160+(s-1)/10 +1 : /* B01A31-B97F42 */ l*10+(f-30-1)*160+(s-1)/10 +1`	LONG `rc`
VARIABLE<br>`(s-1)%10 +1`	LONG `c`
VARIABLE<br>`0 /* (f<=30) ? new int[]{ (int)lut[(int)rc].d1, (int)lut[(int)rc].d2, (int)lut[(int)rc].d3, (int)lut[(int)rc].d4, (int)lut[(int)rc].d5, (int)lut[(int)rc].d6, (int)lut[(int` <br>And 400 other symbols [3]	DOUBLE `tlix`

VARIABLE<br>`(childID.ToCharArray()[1] - 48)*100000 + (childID.ToCharArray()[2] - 48)*10000 + (childID.ToCharArray()[3] - 48)*1000 + (childID.ToCharArray()[4] - 48)*100 + (childID.ToCharArray()[5] - 48)*10 + (chil`<br><br>And 141 other symbols [4]	LONG idcode
VARIABLE<br>`lut[(int)idcode].letter == 0 ? "VV" : lut[(int)idcode].letter == 1 ? "II" : null`	STRING TL

IDENTIFICATION

TREATMENT

E `consent==1`

STATIC TEXT

E `TL!=null && IDok==true`

Treatment Letter: *%TL%*

STATIC TEXT

E `TL == null || IDok!=true`

No Treatment Letter
Invalid or missing ID!

Is child able to stand?<br><br>E `ageInDays>=365`	SINGLE-SELECT canStand<br>01 ○ Yes<br>02 ○ No
Enter %childFirstname%'s weight (kg)<br><br>E `canStand==2 || ageInDays<365`<br>V1 `self.InRange(1,10)`<br>M1 Are you sure %childFirstname% weighs %weight% kg?	NUMERIC: DECIMAL weight<br><br>_ _
Please, prepare the dose and enter here the dose you prepared (ml)<br><br>E `canStand==1`<br>V1 `dosageAdmin>0`<br>M1 Are you sure?	SINGLE-SELECT dosageAdmin<br>02 ○ 2<br>04 ○ 4<br>06 ○ 6<br>08 ○ 8<br>10 ○ 10<br>12 ○ 12<br>14 ○ 14<br>16 ○ 16<br>19 ○ 19<br>25 ○ 25<br>00 ○ 0
Why did you administer 0 ml?<br><br>E `dosageAdmin==0`	TEXT dosage0why<br><br>..
VARIABLE<br>`Math.Round(weight.Value*5)/10`	DOUBLE dose_ml

STATIC TEXT

E `IsAnswered(weight)`

Treatment dosage: %dose_ml% ml (40mg/ml Azithromycin solution)

Did the child receive the study treatment?<br>E `dosageAdmin>0 || weight>0`	SINGLE-SELECT `isAdministered`<br>01 ○ Yes<br>02 ○ No
Why not?<br>E `isAdministered==2`	SINGLE-SELECT `notAdministered`<br>01 ○ Spit up<br>02 ○ Vomited<br>09 ○ Other reason
Specify other reason<br>E `notAdministered == 9`	TEXT `notAdministeredOther`<br>..

STATIC TEXT

E `notAdministered.InList(3,4)`

Really?
After having given treatment consent and having been weighed
he suddenly moved away or died?

IDENTIFICATION / TREATMENT

IMPORTANT FOLLOW-UP DATES

E `ageInDays <= 28`

VARIABLE<br>`startTime.Value.AddDays(7).ToString("dd-MM-yyyy")`	STRING day7
VARIABLE<br>`startTime.Value.AddDays(14).ToString("dd-MM-yyyy")`	STRING day14
VARIABLE<br>`startTime.Value.AddDays(21).ToString("dd-MM-yyyy")`	STRING day21
VARIABLE<br>`startTime.Value.AddDays(180).ToString("dd-MM-yyyy")`	STRING day180

STATIC TEXT

Dates for follow up:
Day 7: %day7%
Day 14: %day14%
Day 21: %day21%
Day 180: %day180%

IDENTIFICATION / TREATMENT

IMPORTANT FOLLOW-UP DATES

E `ageInDays > 28`

VARIABLE<br>`startTime.Value.AddDays(7).ToString("dd-MM-yyyy")`	STRING day7x
VARIABLE<br>`startTime.Value.AddDays(14).ToString("dd-MM-yyyy")`	STRING day14x
VARIABLE<br>`startTime.Value.AddDays(180).ToString("dd-MM-yyyy")`	STRING day180x

STATIC TEXT

Dates for follow up:
Day 14: %day14x%
Day 180: %day180x%

Basic information

Title 8-GAMIN - Visite de Suivi

IDENTIFICATION

Tap to record the time	DATE: CURRENT TIME `startTime`
VARIABLE<br>`startTime.Value.Year`	LONG `startYear`
VARIABLE<br>`"blue"`	STRING `colorName`
What is the child's first name?	TEXT `childFirstname`
Birthdate - Year<br>V1 `self.InRange(1900, startYear) || self==9999`<br>M1 Must be between 1900 and %startYear%	NUMERIC: INTEGER `yearN`<br>SPECIAL VALUES<br>9999 Don't know
Birthdate - Month<br>E `yearN.InRange(1900,startYear)`	SINGLE-SELECT `monthN`<br>01 ○ January<br>02 ○ February<br>03 ○ March<br>04 ○ April<br>05 ○ May<br>06 ○ June<br>07 ○ July<br>08 ○ August<br>09 ○ September<br>10 ○ October<br>11 ○ November<br>12 ○ December<br>99 ○ Don't know
Birthdate - Day<br>F (@optioncode==30 && monthN!=2) || (@optioncode==31 && monthN.InList(1,3,5,7,8,10,12)) || @optioncode.InRange(1,29) || @optioncode==99<br>E `monthN.InRange(1,12)`	SINGLE-SELECT `dayN`<br>01 ○ 1<br>02 ○ 2<br>03 ○ 3<br>04 ○ 4<br>05 ○ 5<br>06 ○ 6<br>07 ○ 7<br>08 ○ 8<br>09 ○ 9<br>10 ○ 10<br>11 ○ 11<br>12 ○ 12<br>13 ○ 13<br>14 ○ 14<br>15 ○ 15<br>16 ○ 16<br>And 16 other symbols [1] 53

Reported age in years or months?<br><br>E `yearN==9999`	SINGLE-SELECT `reportedAgeMetric`<br>01 ○ Years<br>02 ○ Months
Age de %childFirstname%:<br><br>E `yearN==9999`<br>V1 `reportedAgeMetric==1 ? self < 6 : true`<br>M1 Child must be younger than 6 years old!<br>V2 `reportedAgeMetric==2 ? self < 60 : true`<br>M2 Child must be younger than 60 months old!	NUMERIC: INTEGER `reportedAge`<br><br>_ .
VARIABLE<br>`/* if day of month unknown use 15th -> max.error 2 weeks */ (IsAnswered(reportedAge) && reportedAgeMetric==1) ? ((long)reportedAge * 365) : (IsAnswered(reportedAge) && reportedAgeMetric==2) ? ((`<br><br>And 433 other symbols [1]	LONG `ageInDays`
VARIABLE<br>`IsAnswered(reportedAge) && reportedAgeMetric==1 ? (long) reportedAge*12 : IsAnswered(reportedAge) && reportedAgeMetric==2 ? (long) reportedAge : CenturyMonthCode(startTime.Value.Month, startTime.Value`<br><br>And 205 other symbols [2]	LONG `ageInMonths`
VARIABLE<br>`ageInDays/365`	DOUBLE `ageInYears`

STATIC TEXT

E `ageInDays<31 && ageInDays>0`

%childFirstname% is %ageInDays% days old

STATIC TEXT

E `ageInDays>=31 && ageInDays<=365`

%childFirstname% is %ageInMonths% months old

STATIC TEXT

E `ageInDays>365`

%childFirstname% is %ageInYears% years old

What is %childFirstname%'s sex?	SINGLE-SELECT `childSex`<br>01 ○ Male<br>02 ○ Female
%childFirstname%'s ID number	SINGLE-SELECT `childIDmode`<br>01 ○ Scan barcode<br>02 ○ Scan doesn't work
Scan %childFirstname%'s ID<br><br>E `childIDmode==1`<br>V1 `self.ToUpper().ToCharArray()[0]=='L'`<br>M1 Invalid ID !<br>V2 `TL!=null`<br>M2 Invalid ID !	BARCODE `childIDbarcode`

**Enter %childFirstname%'s ID**<br>E `childIDmode==2`<br>V1 `self.ToUpper().ToCharArray()[0]=='L'`<br>M1 Invalid ID !<br>V2 `TL!=null`<br>M2 Invalid ID!	TEXT `childIDmanual`

IDENTIFICATION

VERIFICATION

E `IsAnswered(childIDmanual) && TL!=null`

**Enter the letter in the code**<br>V1 `self.ToUpper().Substring(0,1) == childIDmanual.ToUpper().Substring(0,1)`<br>M1 Not the same code ! - Please verify !	TEXT `childIDverification`
**Enter the numbers in the code**<br>V1 `self.ToUpper().Substring(0,6) == childIDmanual.ToUpper().Substring(1,6)`<br>M1 Not the same code ! - Please verify !	TEXT `childIDverification1`
VARIABLE<br>`IsAnswered(childIDbarcode) ? childIDbarcode : childIDmanual`	STRING `childID`
VARIABLE<br>`IsAnswered(childIDmanual) ? childIDverification1.ToUpper().Substring(0,6) == childIDmanual.ToUpper().Substring(1,6) : true /* put existing ID check here */`	BOOLEAN `IDok`
VARIABLE<br>`(childID.ToCharArray()[1] - 48)*10 + (childID.ToCharArray()[2] - 48)`	LONG `s`
VARIABLE<br>`(childID.ToCharArray()[4] - 48)*10 + (childID.ToCharArray()[5] - 48)`	LONG `f`
VARIABLE<br>`new int[]{0,1,2,3,4,5,999,6,999,7,8,999,9,10,999,11,999,12,999,999,13,999,14,999,15,999}[(childID.ToUpper().ToCharArray()[3] - 65)]`	LONG `l`
VARIABLE<br>`(f<=30)? /* B01A01-B99Y30 */ l*10+(f-1)*160+(s-1)/10 +1 : /* B01A31-B97F42 */ l*10+(f-30-1)*160+(s-1)/10 +1`	LONG `rc`
VARIABLE<br>`(s-1)%10 +1`	LONG `c`
VARIABLE<br>`0 /* (f<=30) ? new int[]{ (int)lut[(int)rc].d1, (int)lut[(int)rc].d2, (int)lut[(int)rc].d3, (int)lut[(int)rc].d4, (int)lut[(int)rc].d5, (int)lut[(int)rc].d6, (int)lut[(int`<br>And 400 other symbols [3]	DOUBLE `tlix`
VARIABLE<br>`(childID.ToCharArray()[1] - 48)*100000 + (childID.ToCharArray()[2] - 48)*10000 + (childID.ToCharArray()[3] - 48)*1000 + (childID.ToCharArray()[4] - 48)*100 + (childID.ToCharArray()[5] - 48)*10 + (chil`<br>And 141 other symbols [4]	LONG `idcode`

VARIABLE	STRING	TL
`lut[(int)idcode].letter == 0 ? "VV" : lut[(int)idcode].letter == 1 ? "II" : null`		

STATIC TEXT

E `TL == null && (IsAnswered(childIDbarcode) || IsAnswered(childIDmanual))`

Invalid ID! Please verify!

VITAL STATUS

Question	Type / Options	Variable
Which follow-up visit?<br>F (@optioncode.InList(1,2,4) && ageInDays>56) | | (@optioncode.InRange(1,4) && ageInDays<=56)	SINGLE-SELECT<br>01 ○ Treatment day + 14<br>02 ○ Treatment day + 180	visitDay
How is interview conducted?<br>E `visitDay == 1 || visitDay == 3`	SINGLE-SELECT<br>01 ○ On Phone<br>02 ○ In person	interviewType
%childFirstname%'s status	SINGLE-SELECT<br>01 ○ Alive and present<br>05 ○ Alive but not present<br>06 ○ Alive but refuses to continue<br>02 ○ Died<br>03 ○ Moved<br>04 ○ Unknown	vitalStatus
Date of Death?<br>E `vitalStatus==2`	SINGLE-SELECT<br>01 ○ Enter date<br>02 ○ Unknown	deathEntry
Date of death<br>E `deathEntry==1`<br>V1 `self<=startTime`<br>M1 Must be in past	DATE<br>....................................	deathDate
When can we do verbal autopsy?<br>E `vitalStatus==2`<br>V1 `self > startTime`<br>M1 Should be in the future!	DATE<br>....................................	VAdate
Since the last time our team visited, has %childFirstname% presented any following symptoms?<br>E `visitDay==1 && vitalStatus==1`	MULTI-SELECT<br>01 ☐ Fever<br>02 ☐ Diarrhea<br>03 ☐ Vomiting<br>04 ☐ Abdominal pain<br>05 ☐ Rash<br>06 ☐ Constipation<br>07 ☐ None	q_5_6
Has %childFirstname% visited the health center since the last time we visited for a health problem?<br>E `visitDay==1 && vitalStatus==1`	SINGLE-SELECT<br>01 ○ Yes<br>02 ○ No	centerVisit
Why did %childFirstname% visit the health center?<br>E `centerVisit == 1`	MULTI-SELECT<br>01 ☐ Fever<br>02 ☐ Diarrhea<br>03 ☐ Pneumonia<br>04 ☐ Malaria<br>05 ☐ Other	reasonVisit

Please specify reason for visit<br>E `reasonVisit.Contains(5)`	TEXT `specifyVisit`<br>..
Has %childFirstname% been hospitalized since the last time we visited?<br>E `visitDay==1 && vitalStatus==1`	SINGLE-SELECT `hospitalVisit`<br>01 ○ Yes<br>02 ○ No
Why was %childFirstname% hospitalized?<br>E `hospitalVisit == 1`	MULTI-SELECT `reasonHosVisit`<br>01 ☐ Fever<br>02 ☐ Diarrhea<br>03 ☐ Pneumonia<br>04 ☐ Malaria<br>05 ☐ Other
Please specify reason for hospitalization<br>E `reasonHosVisit.Contains(5)`	TEXT `specifyHosVisit`<br>..

GAMIN

MORDOR Project

July 2, 2020

Version 1.6

Statistical Analysis Plan

UCSF Francis I. Proctor Foundation
Centre de Recherche en Santé de Nouna
University of Heidelberg

Introduction

This document (Statistical Analysis Plan, SAP) describes the planned analysis and reporting for the clinical trial, **GAMIN: Gut and Azithromycin Mechanisms in Infants and Neonates in Burkina Faso**. It includes specifications for the statistical analyses and tables to be prepared for the interim and final Clinical Study Report. This study is a Phase IV clinical trial to compare methods to reduce childhood mortality using mass administration of azithromycin (Pfizer, CAS 83905-01-5) compared to placebo. The content of this Statistical Analysis Plan meets the requirements stated by the US Food and Drug Administration and conforms to the American Statistical Association's Ethical Guidelines.

The following documents were reviewed in preparation of this Statistical Analysis Plan:

- Azithromycin for the reduction of child and infant mortality: Longitudinal Component, Manual of Operations and Procedures
- Azithromycin for the prevention of neonatal mortality in Burkina Faso: an individual-randomized trial, Statistical Analysis Plan
- Statistical Analysis Plan, MORDOR Trial
- ICH Guidance on Statistical Principles for Clinical Trials

The planned analyses described in this SAP will be included in future manuscripts. Exploratory analyses not necessarily identified in this Statistical Analysis Plan may be performed to support the analysis. Unplanned analyses not delineated in this Statistical Analysis Plan will be documented as such in the final Clinical Study Report and manuscripts. Note that unplanned analyses will be broadly grouped into two categories:

1. Sensitivity analyses for pre-specified outcomes
2. Other unspecified analyses

Sensitivity analyses are defined as alternate ways of modeling the primary outcome to ensure the finding was not dependent on the analytic plan, and these will not be subject to a multiple comparisons correction. Other unspecified analyses will be declared hypothesis generating or subject to an alpha level of 0.001.

This document will be reviewed prior to the enrollment of patients. All subsequent changes will be indicated by detailed change log in the Appendix.

Contents

1 Summary **5**

1.1 Longitudinal Trial 5

2 Statistical Analysis **5**

2.1 Planned Analyses 5

2.1.1 Longitudinal Trial 5

3 Sample Size Considerations **7**

4 Randomization **7**

5 Abbreviations **8**

6 Revision History **10**

1 Summary

1.1 Longitudinal Trial

The trial profile is given in the Manual of Operations. In the longitudinal trial, children are randomized to two arms: **Azithro** and **Control**. The trial is a placebo-controlled, double-masked (i.e., double-blind), individual-randomized clinical trial.

Objectives.

Determine the effect of a single dose of azithromycin for children aged 8 days-59 months on longitudinal changes in the intestinal microbiome over a 6-month period. Children under 5 years of age will be randomized to a single dose of azithromycin or placebo. *We hypothesize that children randomized to a single dose of azithromycin will exhibit a significant difference in the intestinal microbiome after a 6-month period, compared to those randomized to placebo.*

Determine the effect of a single dose of azithromycin for children aged 8 days-59 months on child growth over a 6-month period. *We hypothesize that a single dose of azithromycin will significantly increase child growth over a 6-month period, compared to single dose of placebo.*

2 Statistical Analysis

2.1 Planned Analyses

2.1.1 Longitudinal Trial

Four hundred fifty children between the ages of 8 days and 59 months may be enrolled in the trial and be offered placebo or azithromycin in a masked fashion. Relevant indicators will be collected using whole blood venipucture, nasopharyngeal swabs, fresh frozen stool, rectal swabs, and anthropometry measurements at days 0, 14, and 180. Children followed up within day 120 and day 240 will be included in primary day 180 analysis. Fifty randomly

selected children (25 per arm) will have an additional rectal swab collected at days 2, 4, 6, 8, 10, 12 post-treatment.

Primary Analyses. Pairwise comparison of *Campylobacter*, *C. upsaliensis* and *C. hominis* using Fisher's exact test, corrected for multiple comparisons using Holm-Bonferroni method.

We will compare profile of *Campylobacter*, *C. upsaliensis* and *C. hominis* based on L_1, L_2 distance, and PERMANOVA. We will compare diversity between arms at days 14 and 180 separately, using Shannon's Index and Simpson's Index.

Statistical considerations.

- Model adequacy will be checked by examination of residuals or other goodness of fit tests as needed. Inadequate model fit will prompt us to report alternative models.
- Multiple imputation will be used in case of missing baseline covariates (if applicable). Missing outcome variables will be handled by sensitivity analysis and reporting of conditional results.
- False Discovery Rate will be calculated for each estimated significant effect.

Secondary analyses.

Note that participants outside of WHO Child Growth Standards for WAZ (-6 to +5 SD), HAZ (-6 to +6 SD), or WHZ (-5 to +5 SD) will be excluded from all anthropometric analyses.

***S. pneumoniae* carriage and macrolide resistance** Separate Fisher's exact test at days 14 and 180.

lytA occurence Separate Fisher's exact test at days 14 and 180.

erm B, mef A, pp2A Separate Fisher's exact test at days 14 and 180.

S. pneumoniae, N. meningitides, H. influenzae, Moraxella, S. aureus Pairwise comparison of organisms using Fisher's exact test, corrected for multiple comparisons using Holm-Bonferroni method.

PERMANOVA of L_1 and L_2 distance of bacterial reads. Shannon's and Simpson's estimated gamma diversities will be compared by arm.

Malaria Fisher's exact test of parasitemia and clinical malaria at days 14 and 180.

weight gain Defined as grams per kilogram per day (g/kg/day) will be compared between arms separately at days 14 and 180 using **linear regression**. Baseline weight is accounted for in our metric of weight gain and will not be included in the regression analysis. We will use ordinary least squares method of estimatation and permutation tests to estimate coefficient p-values.

HAZ, WAZ, WHZ, MUAC As secondary analysis, we will perform separate ANCOVA of anthropometric indicators at days 14 and 180, adjusting for baseline.

3 Sample Size Considerations

We assume a mean (standard deviation) alpha Shannon's index of 16.47 (7.12) using results from Thuy et al. [DAR+17]. Assuming 80% power and detectable effect size of 1.02 standard deviations and no loss to follow-up, we propose a sample size of 225 per group, totaling 450 study participants.

4 Randomization

The randomization will be conducted using R. The function `sample` with option `replace=FALSE` will be used to conduct the random shuffling. Note that the choice of the random number seed completely determines the randomization. To ensure the integrity of the randomization, we will use the procedure we used for MORDOR/Malawi.

5 Abbreviations

ANCOVA Analysis of covariance

DSMC Data and Safety Monitoring Committee

HAZ height for age Z score

MUAC Mid upper arm circumference

SAP Statistical Analysis Plan

WAZ weight for age Z score

WHZ weight for height Z score

References

[DAR+17] T. Doan, A. M. Arzika, K. J. Ray, S. Y. Cotter, J. Kim, R. Maliki, and et al. Gut microbial diversity in antibiotic-naive children after systemic antibiotic exposure: A randomized controlled trial. *Clinical Infectious Disease*, 64, 2017.

6 Revision History

29 May 2019 Revised specification and wording of primary and secondary outcomes. Moved weight gain to secondary outcome. Clarified Bonferroni correction for certain species.

13 Jan 2020 Updated the pre-specified sample size for the sub-section of GAMIN sample (GAMIN QOD).

16 Jan 2020 Abbreviated the study name on title page.

20 Mar 2020 Added sample size considerations section and specification of day 180 time window.

08 May 2020 Specified WHO standard range for inclusion for anthropometric (child growth) analysis.

02 Jul 2020 Increased upper range of day 180 window from day 210 to day 240.
