# Supplementary material for: Single-dose azithromycin for child growth in Burkina Faso: a randomized controlled trial
Source: BMC Pediatr. 2021 Mar 17;21:130. doi: 10.1186/s12887-021-02601-7 (PMC7967941; doi:10.1186/s12887-021-02601-7)
Supplement: Supplementary file 2 — Additional file 2: Supplemental Table S1. Baseline demographic and anthropometric characteristics among children lost and not lost to follow-up at 14 days and 6 months. (PDF 2577 kb) [file 12887_2021_2601_MOESM2_ESM.docx]

**Supplemental Table 1.** Baseline demographic and anthropometric characteristics among children lost and not lost to follow-up at 14 days and 6 months

|  | **14 days** | | **6 months** | |
| --- | --- | --- | --- | --- |
|  | *Lost to Follow-up* | *Retained* | *Lost to Follow-up* | *Retained* |
| N | 29 | 421 | 60 | 390 |
| Age, months, median (IQR) | 19 (13 to 36) | 26 (16 to 38) | 21 (13 to 37) | 26 (17 to 38) |
| Female sex, % | 42.9% | 51.5% | 50.0% | 48.8% |
| Mid-upper arm circumference, cm, median (IQR) | 14 (13 to 15.5) | 14 (13.5 to 15) | 14 (13 to 15) | 15 (13.5 to 15) |
| Weight-for-height Z-score (mean, SD) | -0.5 (-1.2 to 0.2) | -0.3 (-1.0 to 0.3) | -0.6 (-1.2 to 0.3) | -0.5 (-1.2 to 0.2) |
| Height-for-age Z-score (mean, SD) | -0.9 (-1.7 to 0.4) | -1.0 (-1.6 to -0.2) | -1.0 (-1.7 to -0.3) | -1.0 (-1.6 to -0.2) |
| Weight-for-age Z-score (mean, SD) | -0.6 (-1.5 to -0.1) | -0.8 (-1.6 to -0.2) | -0.9 (-1.8 to -0.1) | -0.8 (-1.5 to -0.1) |
| Wasted (WHZ<-2), N (%) | 7.7% | 8.3% | 12.1% | 7.7% |
| Stunted (HAZ<-2), N (%) | 11.5% | 15.2% | 22.4% | 13.9% |
| Underweight (WAZ<-2), N (%) | 19.2% | 13.5% | 17.2% | 13.4% |
| Mid-upper arm circumference <12.5 cm | 11.5% | 3.6% | 5.2% | 3.9% |
